# Synthesis and *In Vitro/In silico* Evaluation of Novel 2‑Aryl-6-carboxamide-Substituted Benzoxazole Derivatives with Anticancer Effects and mTOR Inhibitory Potential

**DOI:** 10.1021/acsomega.5c12045

**Published:** 2026-05-22

**Authors:** Ceylan Hepokur, Okan Aykaç, Sema Misir, Şeyda Akin, Burak Kuzu, Naveen Kosar, Recep Kurnaz, Öztekin Algül

**Affiliations:** † Faculty of Pharmacy, Department of Biochemistry, 63986 Sivas Cumhuriyet University, 58140 Sivas, Turkey; ▲ Faculty of Pharmacy, Department of Pharmaceutical Chemistry, Sivas Cumhuriyet University, 58140 Sivas, Turkey; ▼ Faculty of Medicine, Department of Medical Biology, Sivas Cumhuriyet University, 58140, Sivas, Turkey; ‡ Pharmaceutical Chemistry Section, 53000 Van Yuzuncu Yil University, 65080 Van, Turkey; § Chemistry Department,King Fahd University of Petroleum & Minerals, Dhahran 31261, Saudi Arabia; ∥ Interdisciplinary Research Center for Refining and Advanced Chemicals, King Fahd University of Petroleum & Minerals, Dhahran 31261, Saudi Arabia; ⊥ Department of Orthopaedics and Traumatology, Acıbadem State Hospital, Eskişehir 34718,Turkey; # Department of Pharmaceutical Chemistry, Faculty of Pharmacy, 52983 Mersin University, Mersin 33343,Türkiye

## Abstract

In this study, we synthesized novel 2-aryl-6-carboxamide-substituted benzoxazole derivatives and evaluated their anticancer potential against breast cancer cell lines, with a focus on mTOR inhibitory activity. The compounds were synthesized via a three-step method and characterized by NMR analysis. *In silico* studies—including molecular docking, molecular dynamics simulations, and ADMET predictions—were conducted to predict interactions with the target protein and support the observed biological activity. IC_5_0 values were determined in MCF-7 and MDA-MB-231 cells, with MCF-7 cells exhibiting greater sensitivity to the compound. The most active compounds, COH-17 and COH-19, demonstrated cytotoxicity as indicated by LDH release assays. Apoptotic effects were investigated at both molecular and cellular levels: Western blot analysis assessed key apoptotic proteins (Bcl-2, Bax, caspase-3, and p53), while RT-qPCR quantified the expression of BRCA1, BRCA2, PTEN, TP53, BCL-2, BAX, Caspase-3, PI3K, AKT, and BRAD1 genes. Morphological changes associated with apoptosis were confirmed by DAPI staining and fluorescence microscopy, and early and late apoptosis were quantified using Annexin V-FITC/PI flow cytometry. Cell cycle analysis revealed phase-specific arrest, further supporting the antiproliferative activity of the compound. Overall, COH-17 and COH-19 demonstrated potent anticancer effects through mTOR inhibition, induction of apoptosis, and cell cycle arrest, highlighting their potential as targeted therapeutic agents for breast cancer.

## Graphical abstract

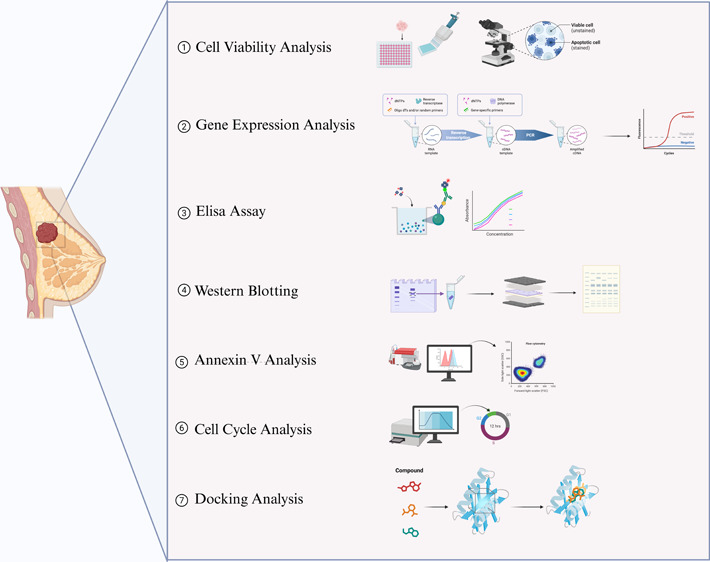

## 1 Introduction

Rapamycin (sirolimus) was first developed in 1975 from natural sources in Rapa Nui as an antifungal drug with immunosuppressive properties.^1^ Its mammalian target is called mTOR (mammalian Target of Rapamycin).^2^ mTOR is a serine/threonine kinase and a member of the Phosphoinositide-3-Kinase-Related Kinase family (PIKK).^3,4^ Genetic and biochemical studies in yeast and mammals led to the discovery of TOR as the target of rapamycin, a macrolide produced by a soil bacterium and used clinically as an immunosuppressant. mTOR mediates a broad-spectrum response to various environmental cues, including growth factors, changing nutritional conditions, and fluctuations in energy.^4^ It serves as the catalytic subunit of two functionally and structurally distinct multiprotein complexes: mTOR Complex 1 and mTOR Complex 2 (mTORC1 and mTORC2). mTORC1 and mTORC2 are distinguished by the auxiliary proteins they contain, specifically Raptor (mTOR-associated regulatory protein) and Rictor (rapamycin-insensitive companion of mTOR), respectively.^5–8^ Both proteins function as scaffold proteins, facilitating the integration of regulators and substrates (Figure 1).^9^ The mTOR complexes differ significantly in their sensitivity to rapamycin, their integration of upstream signals, their substrates, and the biological processes they regulate. Notably, differences in their impaired activity have been implicated in the regulation of growth and metabolism, as well as in diseases such as cancer, obesity, type 2 diabetes, neurodegenerative disorders, and inflammation.^10–12^

**Figure 1:**
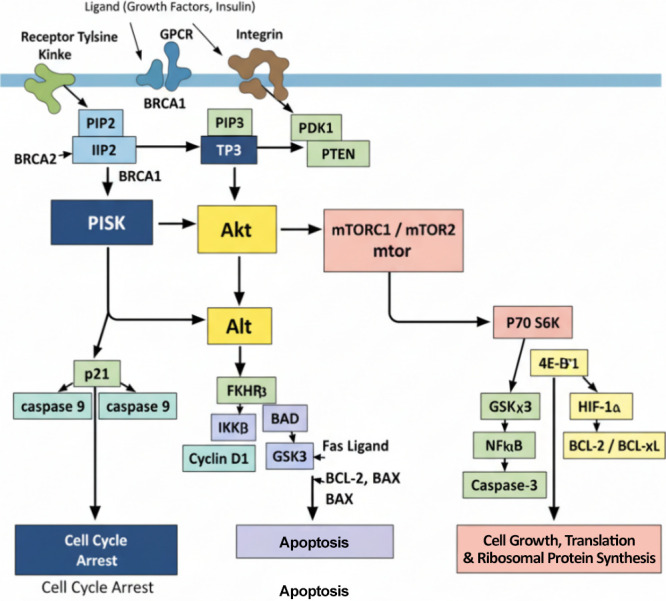
Cellular processes regulated by the PI3K/Akt/mTOR pathway.

mTOR integrates signals from nutrient intake, growth factors, and other cellular processes to regulate downstream signaling and protein synthesis.^13^ The proteins 4EBP1 and p70S6 kinase (S6K) initiate the ribosomal translation of mRNAs encoding proteins essential for cell growth, cell cycle progression, and metabolism. Activation of the mTOR pathway, along with other signaling cascades, influences cell growth and survival, contributing to increased metastatic potential, angiogenesis, and treatment resistance. Consequently, this signaling pathway is considered a critical target for the development of anticancer drugs.^14^

In healthy cells, mTOR activity is regulated by both positive and negative factors.^2^ Positive regulators include growth factors and their receptors, such as insulin-like growth factor 1 (IGF-1) and its receptor IGF-1R, the human epidermal growth factor receptor (HER) family and their associated ligands, as well as vascular endothelial growth factor receptors (VEGFR) and their ligands, which transmit signals to mTOR via the PI3K-Akt pathway. Negative regulators include phosphatase and tensin homologue (PTEN), which suppresses signaling through the PI3K-Akt pathway, and the tuberous sclerosis complex proteins TSC1 (hamartin) and TSC2 (tuberin). Phosphorylation of TSC2 by Akt inhibits its suppressive effect on mTOR.^11^ The mTORC1 complex comprises mTOR, Raptor, mLST8, and PRAS40. It is highly sensitive to rapamycin and is thus the target of first-generation mTOR inhibitors. mTORC1 activates S6 kinase (S6K) and inactivates 4E-binding protein 1 (4EBP1), promoting protein translation and cell growth.^2^ The mTORC2 complex consists of mTOR, Rictor, Sin1, and mLST8. It is less sensitive to rapamycin, and although its role in normal cell function and oncogenesis has not been fully elucidated, ongoing studies are investigating this aspect. It is known to increase cell proliferation and survival by activating the Akt pathway. While activation of the canonical mTOR pathway has been described as possible via the Akt-independent Ras/MEK/ERK pathway, it remains dependent on mitogen-driven signaling through PI3K/Akt.^12^ mTOR activation leads to increased synthesis of multiple proteins, the most well-known of which is Cyclin D1, a key player in the pathogenesis of many tumors that promotes cell growth.^15^ Hypoxia-inducible factor (HIF), in contrast, increases the expression of pro-angiogenic growth factors such as vascular endothelial growth factor (VEGF).^16^ Since its discovery in 1975, rapamycin has been extensively studied for its role in mTOR inhibition. The primary mechanism of action of rapamycin and related compounds involves forming a complex with FK506-binding protein 12 (FKBP12), which binds to the C-terminal region of mTORC1 and interferes with the kinase activity of the multimeric mTORC1 complex, but not mTORC2.^17^ However, the undesirable pharmacological properties of rapamycin necessitated the development of safer derivatives known as “rapalogs.” Three rapalogs—RAD001 (everolimus), CCI779 (temsirolimus), and AP-23573 (deforolimus)—have demonstrated cytostatic activity in preclinical models and exhibited antitumor effects when used in combination with chemotherapy (Figure 2).^17^

**Figure 2:**
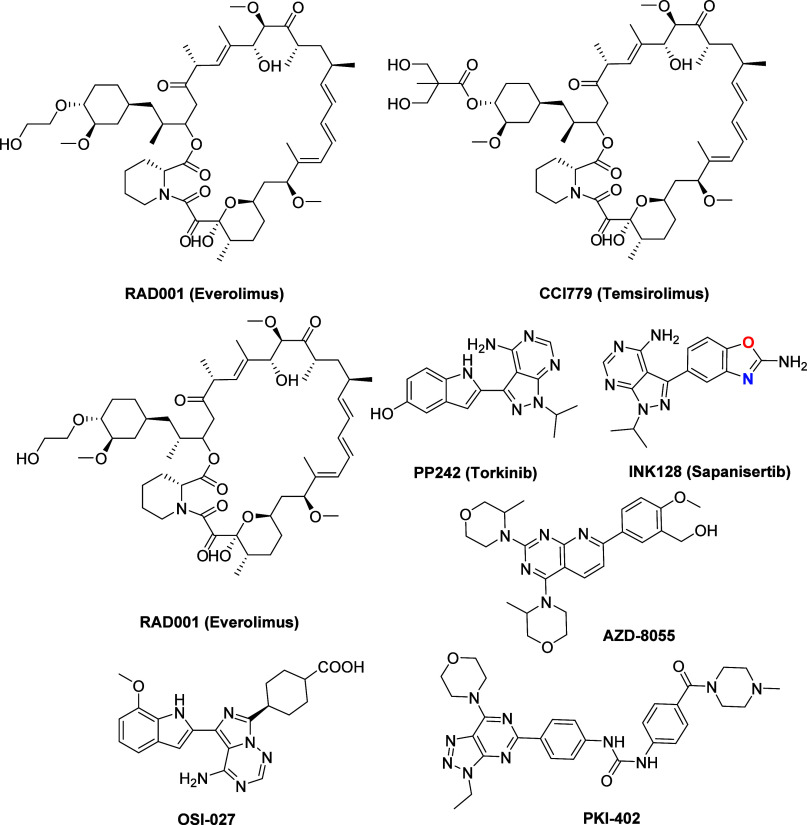
Important mTOR inhibitors.

In the BOLERO-2 study, it was demonstrated that the addition of everolimus to exemestane resulted in improved survival compared to treatment with exemestane alone, as everolimus overcame resistance to hormone therapy in patients with metastatic breast cancer. Despite the advantages of these mTORC1 allosteric inhibitors, the discovery that feedback-mediated activation of PI3K and Akt also affects mTORC2 activity has shifted focus toward ATP-competitive compounds that inhibit mTOR signaling entirely. The first molecule in this class is PP242. Currently, INK128 (Sapanisertib), a derivative of PP242, along with several other pan-mTOR inhibitors such as AZD-8055 and OSI-027, are in early phase clinical trials for solid malignancies, including breast cancer (Figure 2).^18^ PKI-402 is an example of a small molecule defined as a potent inhibitor of the PI3K/mTOR signaling pathway (Figure 2). PKI-402 is a reversible, ATP-competitive inhibitor of class I PI3Ks, including the E545 K and H1047R PI3K-α mutants, as well as mTOR (Mallon et al., 2010). PKI-402 has demonstrated in vitro growth inhibition of human tumor cell lines derived from various tissues, including breast, brain (glioma), pancreatic, and nonsmall cell lung cancer (NSCLC). In vitro, PKI-402 has been shown to inhibit the phosphorylation of PI3K and mTOR effector proteins involved in tumor cell growth, particularly the phosphorylation of Akt at T308 (p-Akt).^19^

When reviewing the studies conducted to date, one notable example of an mTOR inhibitor compound featuring a benzoxazole structure is the patent study carried out by Ren and his team (Figure 3).

**Figure 3:**
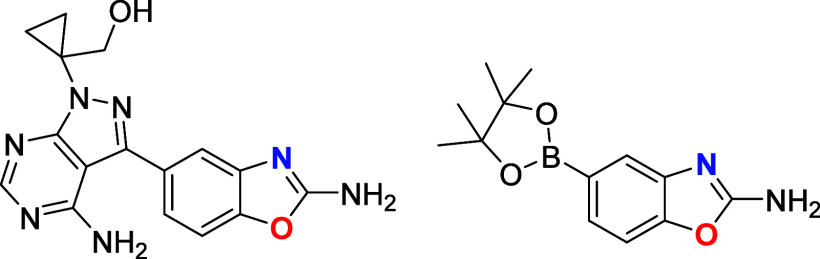
mTOR inhibitors bearing a benzoxazole structure.

The patent study conducted by Ren and his team includes several benzoxazole derivatives, as well as benzoxazole derivatives containing an alicyclic amine moiety. The study demonstrated that the molecular structures shown in the figure exhibit IC_50_ values of 100 nM and 10 nM for mTOR, respectively.^20^

Based on the reviewed literature, mTOR research continues to explore compounds with diverse structural frameworks. In studies conducted by our group, bioisosteres were designed by modifying the benzothiazole ring present in the structure of INK128 (Sapanisertib)-which also contains a benzoxazole ring—as well as other active compounds such as AZD-8055, PKI-402, RAD001, and CCI779, incorporating side groups like morpholine and piperidine. These modifications yielded significant antiproliferative activity. These findings highlight the therapeutic potential of mTOR inhibitors, motivating our research team to develop new drug candidates featuring a benzothiazole core structure that can effectively target this signaling pathway.

The primary aim of this study is to develop novel benzoxazole-derived compounds with potent anticancer properties targeting the mTOR signaling pathway. Structural insights from existing mTOR inhibitors (INK128, AZD-8055, PKI-402, RAD001, CCI-779) were utilized to design and synthesize 2-aryl-6-carboxamide benzoxazole derivatives with reduced lipophilicity and lower molecular weight. NMR analyses confirmed the structures of the synthesized compounds, their binding affinities to mTOR were assessed through molecular docking studies, and their antiproliferative activities were evaluated in breast cancer cell lines. This study aims to develop a new generation of effective and structurally unique anticancer candidate compounds that target the mTOR pathway.

## 2 Results and Discussion

### 2.1 Chemistry

The target compounds were synthesized via a concise four-step synthetic strategy. Initially, *p*-amino-3-hydroxybenzoic acid (compound **a**) was subjected to classical Fischer esterification under reflux in methanol to yield the corresponding methyl ester (compound **b**) with an excellent yield of 95%. Subsequently, compound **b** underwent condensation with various commercially available aromatic aldehyde derivatives to afford the corresponding Schiff bases (compound **c**). These intermediates were employed directly, without isolation, in a NaCN-catalyzed cyclization reaction carried out in DMF at 25 °C, leading to the formation of four distinct 2-aryl benzoxazole derivatives (compound **d**) in 70–90% yields. Finally, these benzoxazole intermediates were subjected to amidation reactions with a range of aromatic aniline derivatives in the presence of aluminum chloride (AlCl_3_) as a Lewis acid catalyst, affording the final target molecules (compounds 1–20) in high yields (90–98%). This synthetic route provides an efficient and reproducible methodology for generating structurally diverse benzoxazole derivatives suitable for subsequent biological evaluation (Figure 4).

**Figure 4:**
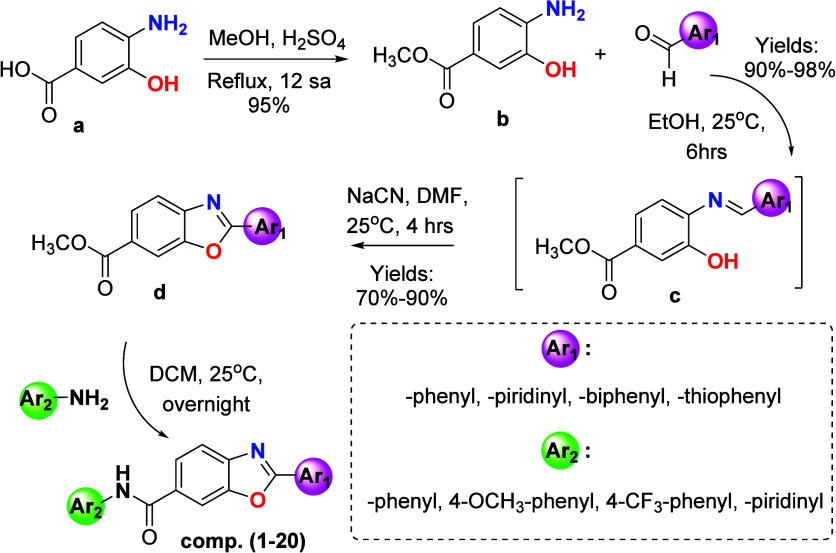
Synthetic route for the preparation of target 2-aryl substituted benzoxazole derivatives (compounds 1–20). (a) Esterification of *p*-amino-3-hydroxybenzoic acid (**a**) to methyl ester (**b**) using MeOH and H_2_SO_4_ under reflux conditions. (b) Condensation of methyl ester (**b**) with various aromatic aldehydes to form Schiff bases (**c**). (c) NaCN-catalyzed cyclization of Schiff bases (**c**) in DMF at 25 °C yielding 2-aryl benzoxazole intermediates (**d**). (d) Amidation of benzoxazole intermediates (**d**) with aromatic anilines in the presence of AlCl_3_ as Lewis acid catalyst, affording the final compounds^1–20^ in high yields.

The amidation reaction depicted in the final step of the overall synthetic pathway (Figure 5) is particularly noteworthy, as the direct reaction of esters with aromatic amines under mild conditions is generally challenging and often results in low yields. The use of aluminum chloride (AlCl_3_) as a Lewis acid catalyst facilitates this transformation by enhancing the electrophilicity of the ester carbonyl. In the proposed mechanism, AlCl_3_ coordinates to the carbonyl oxygen of the methyl ester, thereby increasing the susceptibility of the carbonyl carbon to nucleophilic attack by the aromatic amine (R–NH_2_). The nucleophilic attack generates a tetrahedral intermediate, which subsequently collapses with the release of AlCl_3_ to form the desired amide bond. This mechanism highlights the critical role of AlCl_3_ in promoting amidation under mild conditions, enabling the efficient synthesis of the target benzoxazole-based amide derivatives.

**Figure 5:**
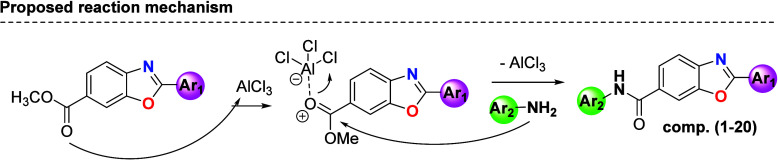
Proposed mechanism for the AlCl_3_-catalyzed amidation of 2-aryl benzoxazole methyl esters with aromatic amines. Coordination of AlCl_3_ to the ester carbonyl enhances electrophilicity, facilitating nucleophilic attack by the aromatic amine. The resulting tetrahedral intermediate collapses with the elimination of AlCl_3_ to yield the final amide product. This mechanism illustrates the crucial role of the Lewis acid in promoting efficient amidation under mild reaction conditions.

All synthesized benzoxazole-based amide derivatives^1–20^ were obtained as light-colored powders in moderate to high yields (70–94%) and were fully characterized to confirm their structures and purity. Melting points were consistent with the formation of crystalline products. ^1^H NMR spectra of the compounds displayed characteristic signals for the amide NH (δ 10.2–10.7 ppm) and aromatic protons corresponding to the benzoxazole core and various substituents, including phenyl, methoxyphenyl, trifluoromethylphenyl, pyridinyl, thiophenyl, naphthyl, and biphenyl groups. Multiplet patterns, doublets, and triplets were consistent with the expected chemical environments of these protons. ^13^C NMR spectra confirmed the presence of amide carbonyl carbons (δ 164–166 ppm), benzoxazole carbons, and aromatic carbons of the diverse substituents. Signals corresponding to methoxy carbons were observed around δ 55–56 ppm, while trifluoromethyl-substituted carbons displayed characteristic splitting patterns. High-resolution mass spectrometry (HRMS) further corroborated the molecular formulas of all derivatives, with observed *m*/*z* values in excellent agreement with calculated values. Collectively, these data demonstrate that the synthetic methodology reliably produced highly pure benzoxazole amide derivatives, with full structural integrity confirmed by complementary spectroscopic techniques.

### 2.2 Biological Evaluations

#### 2.2.1 Cell Culture

The cytotoxic effects of all compounds on breast cancer cells (MCF-7, MDA-MB-231) and healthy breast cells (MCF-10A) were evaluated using the MTT test.

The IC_50_ values were determined by plotting a percentage viability versus concentration graph.

According to the data in the Table 1, the synthesized compounds demonstrate effective cytotoxic activity against the MCF-7 and MDA-MB-231 cells, while exhibiting higher IC_5_0 values in MCF-10A cell. The selectivity index (SI) was used to assess the relative cytotoxic selectivity of the tested compounds toward cancer cells compared to normal cells. A higher SI value indicates greater selectivity for cancer cells.

$$\mathrm{SI}={\mathrm{IC}}_{50}\left(\mathrm{cancer cells}\right)/{\mathrm{IC}}_{50}\left(\mathrm{normal cells}\right)$$

**Table 1:** IC_50_ Values of All Compounds in the MCF-7, MDA-MB-231, and MCF-10A Cell Lines^a^

**IC**_**50**_	**MCF-7**	**MDA-MB-231**	**MCF-10A**	**MCF-7 (SI)**	**MDA (SI)**
1	26.87 ± 0.35	25.32 ± 0.12	35.62 ± 0.22	1.32	1.41
2	20.35 ± 0.14	26.32 ± 0.21	38.23 ± 0.17	1.88	1.45
3	21.74 ± 0.25	25.02 ± 0.13	39.20 ± 0.19	1.80	1.57
4	22.36 ± 0.42	19.58 ± 0.21	41.25 ± 0.19	1.84	2.10
5	20.35 ± 0.27	26.32 ± 0.18	37.36 ± 0.11	1.83	1.42
6	19.36 ± 0.18	27.36 ± 0.23	32.62 ± 0.13	1.69	1.19
7	18.93 ± 0.17	19.32 ± 0.36	30.25 ± 0.18	1.60	1.57
8	16.35 ± 0.54	20.12 ± 0.12	38.35 ± 0.17	2.34	1.91
9	17.36 ± 0.15	34.56 ± 0.31	39.32 ± 0.21	2.26	1.13
10	19.25 ± 0.52	31.23 ± 0.12	33.23 ± 0.19	1.72	1.06
11	17.85 ± 0.17	30.33 ± 0.14	31.02 ± 0.17	1.73	1.02
12	26.35 ± 0.54	23.65 ± 0.23	35.48 ± 0.20	1.34	1.50
13	28.45 ± 0.75	22.68 ± 0.16	41.02 ± 0.17	1.44	1.81
14	31.25 ± 0.19	21.45 ± 0.18	44.52 ± 0.22	1.42	2.08
15	22.35 ± 0.24	19.65 ± 0.17	41.03 ± 0.23	1.84	2.09
16	21.36 ± 0.24	24.36 ± 0.24	37.36 ± 0.17	1.75	1.53
17	15.56 ± 0.11	20.31 ± 0.26	66.32 ± 0.19	4.26	3.26
18	20.35 ± 0.24	24.25 ± 0.23	38.36 ± 0.24	1.89	1.58
19	17.02 ± 0.29	26.35 ± 0.14	40.39 ± 0.12	2.37	1.53
20	19.23 ± 0.34	28.65 ± 0.17	39.25 ± 0.13	2.04	1.37
cis-platin	6.17 ± 0.29	8.16 ± 0.19	98.25 ± 0.16	15.92	12.04

a The selectivity index (SI).

This suggests that the compounds have selective cytotoxic potential against cancer cells. Specifically, compounds 8, 17, and 19 showed the strongest antiproliferative effects in the MCF-7 cell, with low IC_5_0 values of 16.35, 15.56, and 17.02 μg/mL, respectively, and SI of approximately 2. In the MDA-MB-231 cells, compounds 4, 14, and 15 exhibited SI values above 2, indicating relatively greater selectivity against triple-negative breast cancer cells. COH-17 and 19, which had the highest SI values, were selected for further biological activity analyses. According to widely accepted criteria, SI values greater than 1 indicate selective toxicity toward cancer cells, while SI values equal to or greater than 2 are considered to demonstrate good or high selectivity, suggesting a favorable therapeutic approach.^21^

Since breast cancer frequently metastasizes to the brain, bone, and lungs, the C6 (glioma), MG-63 (osteosarcoma), and A549 (lung adenocarcinoma) cell lines, representing these metastatic sites, were used further to evaluate the cytotoxic potential of COH-17 and 19. The IC_5_0 values obtained from these assays confirmed that both compounds exhibited significant cytotoxicity, indicating their potential effectiveness against metastatic breast cancer cells.

As summarized in Table 2, COH-17 and COH-19 exhibited pronounced cytotoxic effects across all tested cancer cell lines, with IC_5_0 values falling within the low to moderate micromolar range. COH-17 exhibited IC_5_0 values of 15.88 ± 0.10, 17.35 ± 0.09, and 16.43 ± 0.24 μg/mL in C6, MG-63, and A549 cells, respectively, while COH-19 showed IC_5_0 values of 18.52 ± 0.02, 19.02 ± 0.07, and 29.02 ± 0.14 μg/mL in the same cell lines. Pairwise comparisons revealed that IC_5_0 values of COH-17 were significantly lower than those of COH-19 across all cell lines (P1 < 0.0001). In addition, comparisons between cancer cell lines and the nontumorigenic MCF-10A cell line demonstrated significantly lower cytotoxicity toward normal cells following treatment with both COH-17 and COH-19 (P2 < 0.0001 and P3 < 0.0001, respectively). One-way ANOVA/Kruskal–Wallis analyses confirmed statistically significant differences in IC_5_0 values among the six cell lines for both compounds (P4 < 0.0001). These findings indicate that COH-17 generally exhibits a stronger cytotoxic profile than COH-19.

**Table 2:** IC_50_ Values of COH-17 and 19 in Different Cancer Cell Lines^a^

**cell lines**	**17 (IC**_**50**_ **)**	**19 (IC**_**50**_ **)**	**P** ^**1**^ **-value**	**P** ^**2**^ **-value**	**P** ^**3**^ **-value**
**C6**	15.88 ± 0.10	18.52 ± 0.02	0.0001	P**^2^** <0.0001	P**^3^** <0.0001
**MG-63**	17.35 ± 0.09	19.02 ± 0.07	P^1^ < 0.0001	P**^2^** <0.0001	P**^3^** <0.0001
**A549**	16.43 ± 0.24	29.02 ± 0.14	P^1^ < 0.0001	P**^2^** <0.0001	P**^3^** <0.0001
**MCF-7**	15.56 ± 0.11	17.02 ± 0.29	0.00001	P**^2^** <0.0001	P**^3^** <0.0001
**MDA-MB-231**	20.31 ± 0.26	26.35 ± 0.14	0.00001	P**^2^** <0.0001	P**^3^** <0.0001
**MCF-10A**	66.32 ± 0.19	40.39 ± 0.12	P^1^ < 0.0001	ref.	ref.
**P** ^**4**^ **-value**	P**^2^** <0.0001	P**^2^** <0.0001			

a Normality of data distribution was assessed using the Shapiro–Wilk test. Independent samples *t* test was applied for normally distributed data, while the Mann–Whitney U test was used for non-normally distributed data. **P** ^**1**^ represents the comparison between COH-17 (IC_5_0) and COH-19 (IC_5_0); **P** ^**2**^ indicates the comparison of IC_5_0 values between the MCF-10A cell line and other cell lines following COH-17 treatment; and **P** ^**3**^ represents the statistical significance between the MCF-10A cell line and other cell lines in terms of COH-19 (IC_5_0). **P** ^**4**^ value represents the statistical significance obtained from one-way ANOVA or Kruskal–Wallis test comparing IC_5_0 values among six cell lines treated with COH-17 or COH-19. Data are presented as mean ± SD. A p value <0.05 was considered statistically significant.

Furthermore, compared to the results obtained in MCF-7 and MDA-MB-231 cells, COH-17 displayed comparable cytotoxic potency across both primary and metastatic models, whereas COH-19 exhibited slightly reduced activity, particularly in A549 cells. Importantly, both compounds demonstrated considerably lower cytotoxicity toward MCF-10A, with IC_5_0 values of 66.32 ± 0.19 μg/mL for COH-17 and 40.39 ± 0.12 μg/mL for COH-19, indicating a degree of selectivity toward malignant cells. Collectively, these findings suggest that COH-17 and COH-19 possess promising cytotoxic profiles not only against breast cancer cells but also against cell lines representing potential metastatic targets.

The viability values of two compounds, numbered 17 and 19, at two different concentrations (3.125 μg/mL and 12.50 μg/mL) were calculated and are presented in Figure 6.

**Figure 6:**
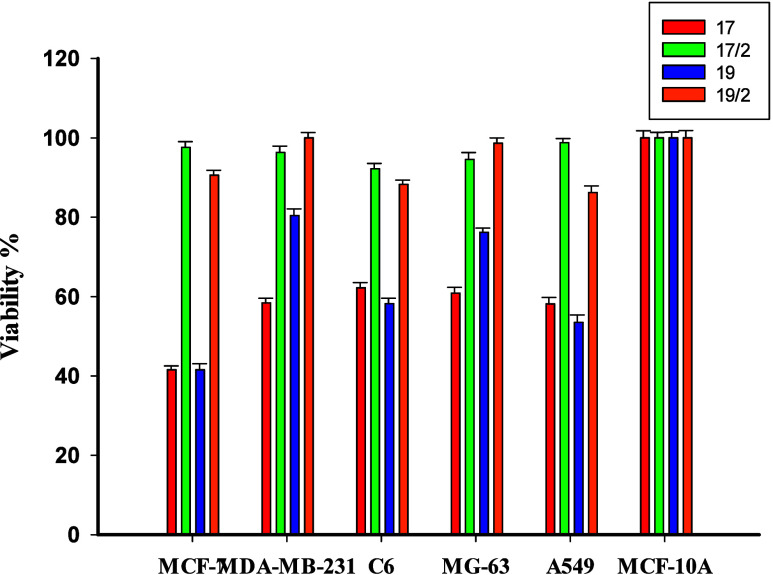
Cytotoxic effects of active COH-17 and COH-19 on various cancer cell lines, expressed as percentage of cell viability (17:12.50 μg/mL 17/2: 3.125 μg/mL 19:12.50 μg/mL 19/2: 3.125 μg/mL). Except for the MCF-10 cell line, a statistically significant difference in cell viability was observed between COH-17 and COH-17/2 treatments across all cancer cell lines (*p* < 0.0001). Similarly, except for MCF-10, a statistically significant difference in cell viability was detected between COH-19 and COH-19/2 treatments across all cancer cell lines (*p* < 0.0001). A p-value <0.05 was considered statistically significant.

#### 2.2.2 mTOR Analysis

The IC_5_0 doses of all compounds used in the study were applied to MCF-7 cells with high efficacy. mTOR protein levels were measured after treatment (Table 3).

**Table 3:** mTOR Protein Levels Measured after Treatment with All Compounds at Their IC_5_0 Concentrations

	**mTOR/ng mL** ^**–1**^		**mTOR/ng mL** ^**–1**^
**1**	0.3125 ± 0.0120	**11**	0.3429 ± 0.0261
**2**	0.3025 ± 0.0141	**12**	0.2894 ± 0.032
**3**	0.3128 ± 0.0120	**13**	0.2934 ± 0.025
**4**	0.3625 ± 0.0122	**14**	0.2960 ± 0.047
**5**	0.3865 ± 0.0141	**15**	0.2945 ± 0.024
**6**	0.2878 ± 0.0131	**16**	0.2914 ± 0.016
**7**	0.2775 ± 0.0111	**17**	**0.2259** ± 0.0125
**8**	0.2962 ± 0.0141	**18**	0.3124 ± 0.0112
**9**	0.3025 ± 0.0121	**19**	**0.2740** ± 0.0103
**10**	0.2854 ± 0.0114	**20**	0.2835 ± 0.0154

mTOR protein levels ranged from 0.226 to 0.387 ng·mL^–1^ across all samples, with each value expressed as mean ± SD. These results indicate that treatment with different compounds at their IC_5_0 concentrations induces significant changes in mTOR levels. The findings suggest that these compounds may exert a measurable effect on mTOR regulation within cellular response mechanisms.

COH-17 and COH-19, which were previously identified as compounds potentially associated with modulation of the mTOR signaling pathway, were reapplied to MCF-7 cells at three different doses. The purpose of this experiment was to determine whether the compounds inhibit the mTOR pathway in a dose-dependent manner. Accordingly, three doses of 5, 15, and 25 μg/mL (selected to be below and above the IC_50_ value)- were administered to MCF-7 cells, and mTOR protein levels were measured (Table 4).

**Table 4:** mTOR Protein Levels Obtained by Applying COH-17 and 19 to the MCF-7 Cell Line at Different Dose^a^

**treatment doses (μg mL** ^**–1**^ **)**	**for 17 compounds mTOR (ng mL** ^**–1**^ **)**	**for 19 compounds mTOR (ng mL** ^**–1**^ **)**	**P** ^**1**^ **–value**
5	1.6333 ± 0.0245	0.4341 ± 0.0141	*p* < 0.0001
15 (IC_50_ dose)	0.2259 ± 0.0125	0.2740 ± 0.0103	0.006
25	0.2052 ± 0.0111	0.1219 ± 0.0256	0.00002
**P** ^**2**^ **–value**	*p* < 0.00001	*p* < 0.00001	

a Differences between two independent groups were evaluated using the independent *t* test or the Mann–Whitney U test (P1 value). Differences among more than two independent groups were assessed using one-way ANOVA or the Kruskal–Wallis test (P2 value). A p-value <0.05 was considered statistically significant.

After the compounds were applied to the cells, the observed decrease in total mTOR protein levels suggests a potential suppression of mTOR-related signaling. Following treatment with 5, 15 (IC50 dose), and 25 μg/mL doses of COH-17, mTOR protein levels in the MCF-7 cell line were determined to be 1.6333 ± 0.0245 ng/mL, 0.2259 ± 0.0125 ng/mL, and 0.2052 ± 0.0111 ng/mL, respectively, demonstrating a statistically significant, dose-dependent decrease in mTOR protein expression (*p* < 0.00001). Similarly, treatment with 5, 15, and 25 μg/mL doses of COH-19 resulted in mTOR protein levels of 0.4341 ± 0.0141 ng/mL, 0.2740 ± 0.0103 ng/mL, and 0.1219 ± 0.0256 ng/mL, respectively, with a statistically significant difference observed among the three doses (*p* < 0.001). Comparative analysis of equivalent doses revealed that 5 and 25 μg/mL doses of COH-17 were associated with higher mTOR protein levels than the corresponding 5 and 25 μg/mL doses of COH-19 (*p* < 0.0001 and p = 0.00002, respectively). At the IC_5_0 dose (15 μg/mL), COH-17 suppressed mTOR levels more effectively than COH-19. These results suggest that the compounds have a potent inhibitory effect on mTOR signaling pathways and may possess effective regulatory potential in cellular proliferation, growth, and metabolic processes.^22^ Furthermore, the reduction in mTOR levels supports the hypothesis that this mechanism may underlie the compounds’ antiproliferative effects. Consequently, application of the IC_5_0 dose reduced mTOR expression by approximately 86%, highlighting the compounds’ biological efficacy and target specificity. Notably, COH-17 suppressed mTOR protein levels more effectively than COH-19, suggesting that COH-17 may be a more promising agent for therapeutic strategies aimed at inhibiting mTOR pathways.

#### 2.2.3 Lactate Dehydrogenase (LDH) Analysis

LDH leakage occurs not only during necrosis but also in the late stages of apoptosis.^23,24^ Cytotoxicity is generally classified based on LDH release as follows: below 20% is considered low, 20–80% is moderate, and above 80% is high cytotoxicity. COH-17 and COH-19 compounds were applied to MCF-7 cells at three different doses (5, 10, and 25 μg/mL), and the percentage of LDH cytotoxicity is shown in Figure 7.

**Figure 7:**
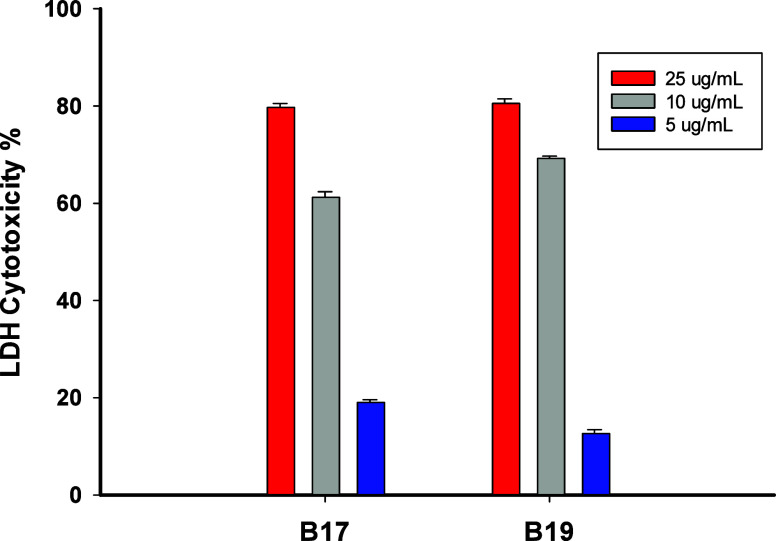
LDH cytotoxicity (%) induced by COH-17 and COH-19 compounds in MCF-7 cells. In MCF-7 cells treated with COH-17 (5–25 μg/mL), LDH cytotoxicity decreased in a dose-dependent manner, and the differences among doses were statistically significant (*p* < 0.0001). Similarly, treatment with COH-19 (5–25 μg/mL) resulted in a significant dose-dependent reduction in LDH cytotoxicity (*p* = 0.0003). A p-value <0.05 was considered statistically significant.

At the highest dose of COH-19 (25 μg/mL), approximately 80.6% cytotoxicity was observed, which corresponded with a high release of LDH. Cytotoxicity levels were 69% and 12% at medium and low doses, respectively, placing them within the medium-high and low cytotoxicity ranges. These results indicate that COH-19 disrupts membrane integrity in a dose-dependent manner, leading to severe cytotoxicity, particularly at higher concentrations. Similarly, COH-17 caused significant cell membrane damage at high doses, moderate damage at medium doses, and minimal damage at low doses. This strongly supports the conclusion that COH-17 exhibits a dose-dependent cytotoxic effect in the mammalian mammary cancer cell line. Given that COH-17 shows lower cytotoxicity at medium and low doses, it is generally considered less toxic.

#### 2.2.4 Analysis of BRCA1, BRCA2, PTEN, TP53, BCL-2, BAX, CASPASE-3, PI3K, AKT, and BRAD1 by RT-PCR

The expression levels of the BRCA1, BRCA2, PTEN, TP53, BCL-2, BAX, CASPASE-3, PI3K, AKT, and BRAD1 genes were analyzed in MCF-7 cells treated with the COH-17 and COH-19 compounds. The results are presented in Figure 8.

**Figure 8:**
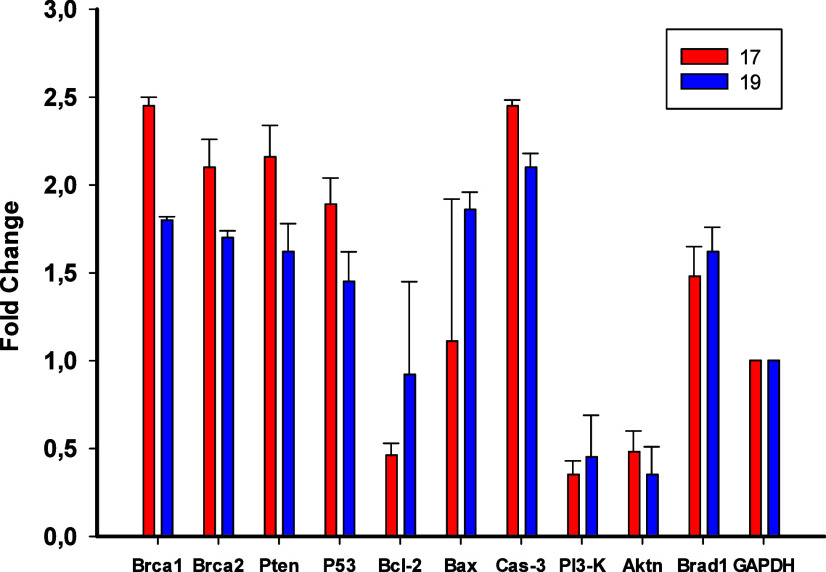
Effects of COH-17 and COH-19 compounds on apoptosis-related genes in MCF-7 cells.

After COH-17 administration, a significant increase was observed in the relative expression levels of BRCA1 (2.45 ± 0.05, *p* < 0.0001), BRCA2 (2.10 ± 0.15, *p* < 0.0001), PTEN (2.15 ± 0.20, *p* < 0.0001), P53 (1.90 ± 0.15, *p* < 0.0001), Caspase-3 (2.45 ± 0.05, *p* < 0.00001) and BRAD1 (1.50 ± 0.15, *p* < 0.0001) genes in MCF-7 cells. On the other hand, there was a decrease in the expression levels of BCL-2 (0.45 ± 0.10, p = 0.0002), PI3K (0.35 ± 0.10, p = 0.0004) and AKT (0.50 ± 0.10, p = 0.0001) genes (*p* < 0.001). BAX gene expression (1.10 ± 0.35, p = 0.28) showed high variance (p = 0.09).

After COH-19 administration, increased expression levels of BRCA1 (1.80 ± 0.05, *p* < 0.0001), BRCA2 (1.70 ± 0.05, *p* < 0.0001), PTEN (1.60 ± 0.20, p = 0.0004), P53 (1.45 ± 0.15, p = 0.002), BAX (1.85 ± 0.10, *p* < 0.0001), Caspase-3 (2.10 ± 0.05, *p* < 0.0001) and BRAD1 (1.65 ± 0.15, p = 0.0002) genes were observed in MCF-7 cells. On the other hand, decreased expression levels of PI3K (0.45 ± 0.20, p= 0.0009) and AKT (0.35 ± 0.15, p = 0.0001) genes were detected. BCL-2 gene expression (0.90 ± 0.20, p = 0.41) exhibited relatively high variance.

Treatment of MCF-7 cells with COH-17 and COH-19 compounds increases the expression of TP53 and PTEN, thereby suppressing the cell cycle and enhancing the DNA damage response. Concurrently, apoptosis is induced via the mitochondrial pathway, as evidenced by an increase in Bax and a decrease in BCL-2.^25^ Given the low expression of caspase-3 in MCF-7 cells, the observed apoptosis is suggested to be mediated independently of caspase-3. Inhibition of the PI3K/AKT pathway further contributes to the suppression of cell proliferation. These findings suggest that PTEN activation suppresses the mTOR pathway by inhibiting PI3K/AKT signaling, thereby halting cell growth and inducing apoptosis through TP53, Bax, and caspase-3.

#### 2.2.5 Determination of BCL-2, BAX, CASPASE-3, and p53 Protein Expression by Western Blot Method

Western blot analysis was performed to assess the expression levels of proteins involved in the disrupted apoptotic pathway in MCF-7 cells treated with COH-17 and COH-19 compounds (Figure 9). This analysis aimed to demonstrate how these compounds influence apoptosis, specifically by modulating the levels of BCL-2, p53, BAX, and caspase-3 proteins.

**Figure 9:**
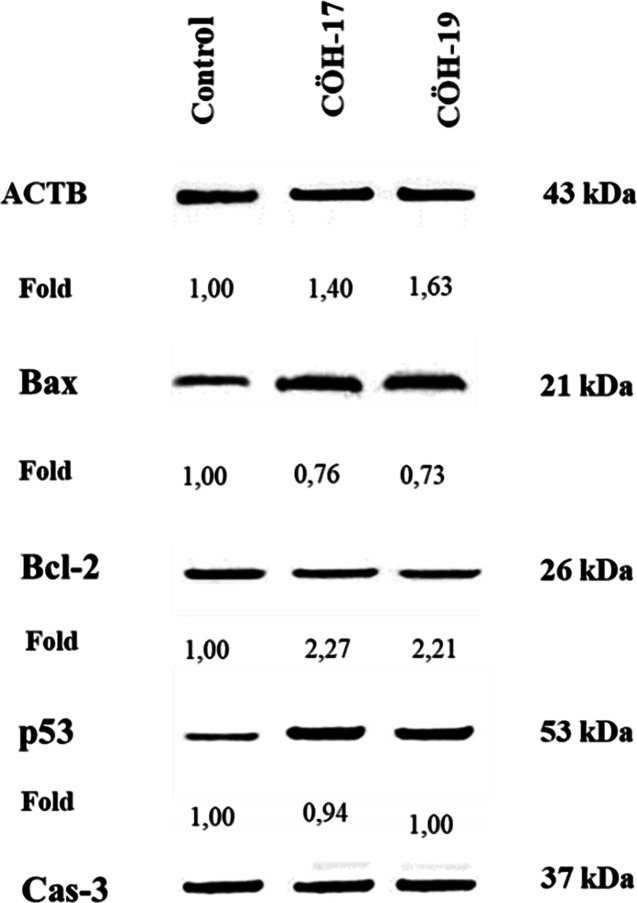
Effects of COH-17 and COH-19 on the expression levels of apoptosis-related proteins (p53, Bax, Bcl-2, and caspase-3) in MCF-7 cells, as determined by Western blot analysis. β-actin was used to normalize protein levels.

According to the Western blot data, treatment with COH-17 and COH-19 altered the levels of Bax, BCL-2, and p53 proteins compared to the control group. Bax protein levels increased following COH-17 and COH-19 treatments (1.40- and 1.63-fold, respectively), indicating enhanced cell death signaling. Conversely, the level of the antiapoptotic protein BCL-2 decreased (0.76- and 0.73-fold, respectively), suggesting a diminished cellular survival mechanism. Additionally, the tumor suppressor protein p53, which responds to cellular damage, was upregulated by both treatments (2.27- and 2.21-fold, respectively), indicating cellular stress and activation of the apoptotic pathway.^26^ No significant changes were observed in caspase-3 protein levels compared to the control. Overall, these findings demonstrate that COH-17 and COH-19 exert pro-apoptotic effects in cells. Cellular survival depends on the balance between Bax and BCL-2 proteins; these compounds activate cell death signaling pathways by increasing pro-apoptotic protein expression while decreasing antiapoptotic protein expression.

#### 2.2.6 DAPI Staining and Nuclear Morphology Analysis

4′,6-Diamidino-2-phenylindole (DAPI) is a blue-fluorescent dye that binds to DNA within the cell nucleus, particularly in AT-rich regions. It enables visualization of characteristic apoptotic changes, such as chromatin condensation and nuclear fragmentation. During apoptosis, nuclear structures are altered due to DNA fragmentation, resulting in increased accumulation of DAPI and a correspondingly brighter fluorescence signal in apoptotic cells.

DAPI is a nuclear dye that binds to DNA with high affinity and emits blue fluorescence, enabling the visualization of cell nuclei and chromatin structures. In the images, cell nuclei appear as distinct blue structures, while apoptotic nuclei exhibit more condensed and brightly stained chromatin fibers. The chromatin within the nucleus was observed to display a dispersed and widespread pattern, indicating a reduction in densely packed chromatin regions and a predominance of more loosely organized euchromatin. Such decondensed chromatin is typically associated with high transcriptional activity and increased gene expression. Decondensed chromatin facilitates access to genetic material, allowing RNA polymerase and other transcription factors to bind DNA and enhance gene transcription. In the present study, treatment with COH-17 and COH-19 compounds induced dynamic changes in chromatin structure in response to the cells. This widespread chromatin organization serves as a morphological indicator of significant adaptations in the cells’ gene expression profiles (Figure 10).

**Figure 10:**
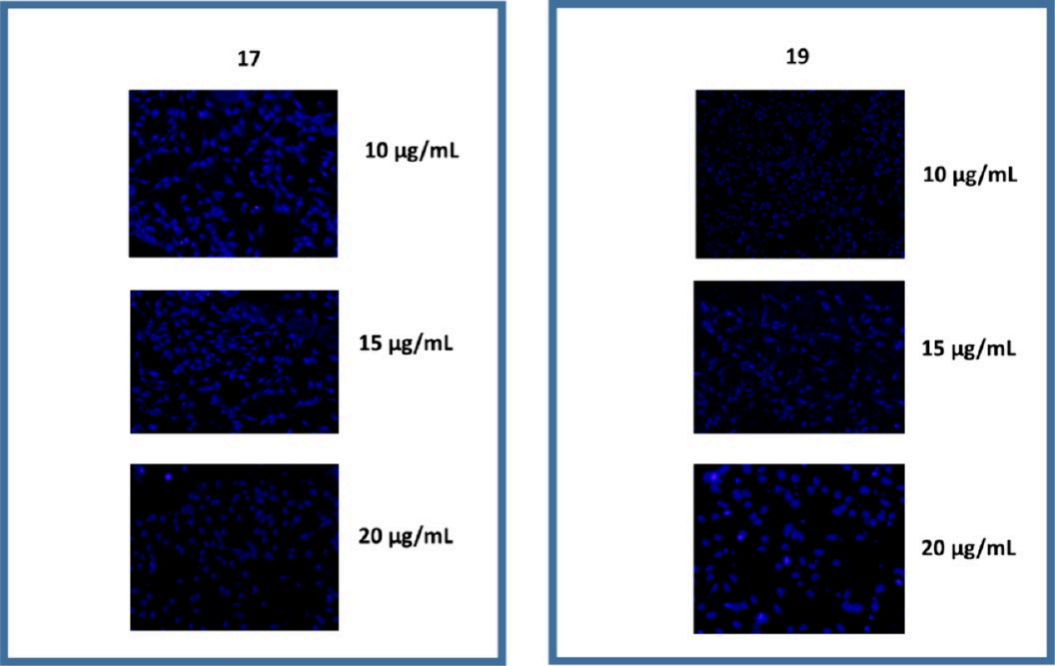
DAPI staining of MCF-7 cells treated with COH-17 and COH-19.

#### 2.2.7 Annexin V Staining for Detection of Apoptosis

The apoptotic activity of the COH-17 and COH-19 compounds in MCF-7 cells was assessed using Annexin V. The results for the untreated negative control group and the treated MCF-7 cells are presented in Figure 11a,b.

**Figure 11:**
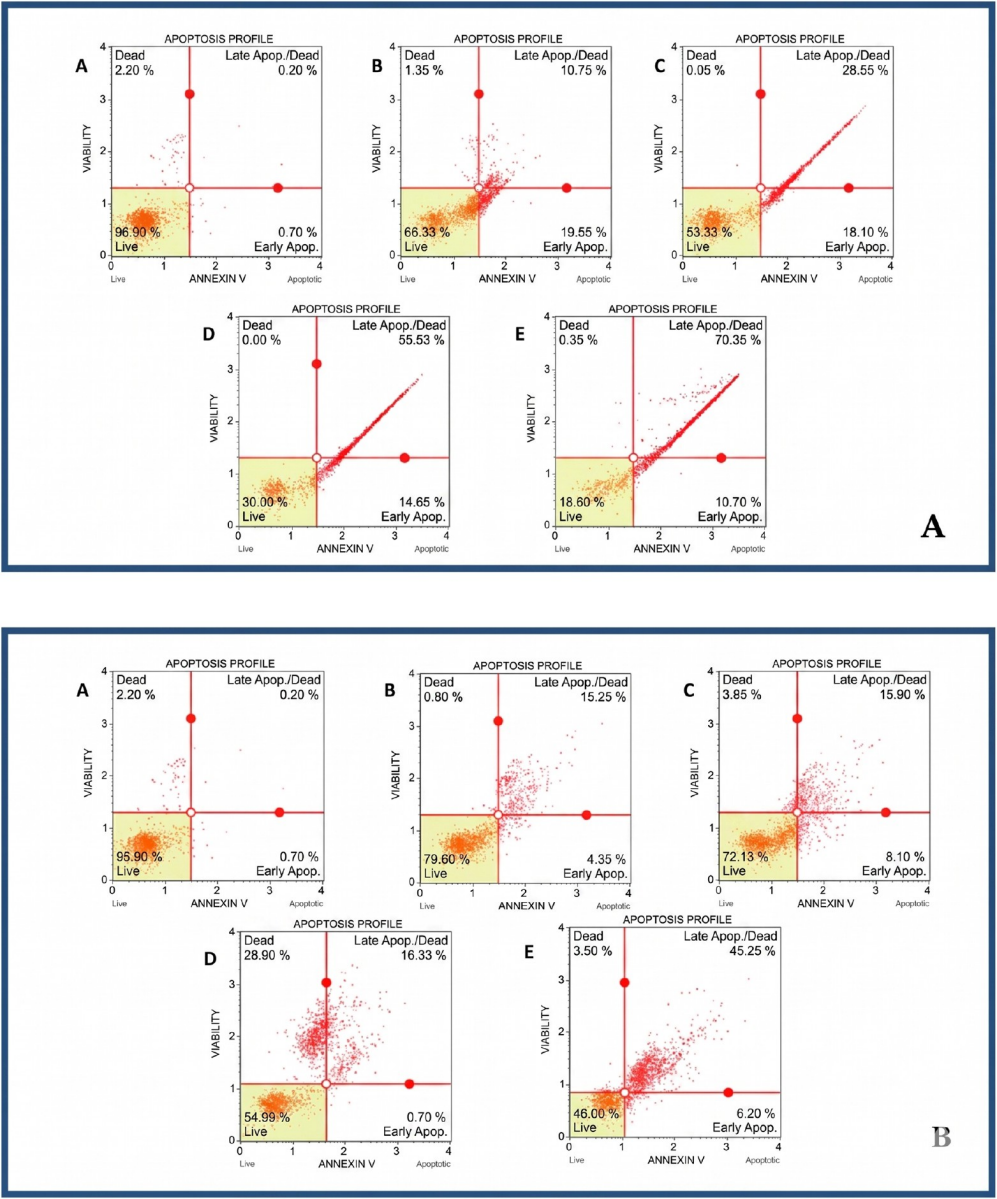
**A.** Annexin V staining flow cytometry results of COH-17 in MCF-7 cells (A: control; B: after treatment with 10 μg/mL COH-17; C: after treatment with 15 μg/mL COH-17; D: after treatment with 20 μg/mL COH-17; E: after treatment with 25 μg/mL COH-17). **B**. Annexin V staining flow cytometry results of COH-19 in MCF-7 cells (A: control; B: after treatment with 10 μg/mL COH-19; C: after treatment with 15 μg/mL COH-19; D: after treatment with 20 μg/mL COH-19; E: after treatment with 25 μg/mL COH-19).

According to the Annexin V analysis results, the compounds COH-17 and COH-19 significantly decreased the viability of MCF-7 cancer cells. It significantly increased the number of apoptotic cells in a dose-dependent manner.

#### 2.2.8 Cell Cycle Analysis

To investigate the effect of the COH-17 compound on the cell cycle of the MCF-7 cells, flow cytometry was performed using a DNA content kit. The cell cycle analysis results for the COH-17 are presented in Figure 12.

**Figure 12:**
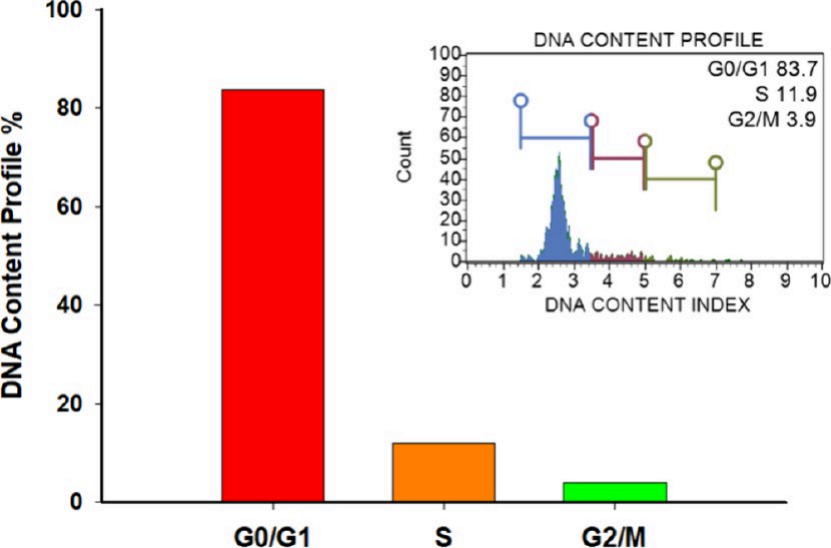
DNA content profile.

The B17 compound induces arrest of MCF-7 cells in the G0/G1 phase of the cell cycle. The G0/G1 phase represents the growth and preparatory stage preceding DNA synthesis (S phase). In chemotherapeutic strategies targeting rapidly dividing cells, such as cancer cells, arresting the cell cycle at the G0/G1 phase is desirable.^27^ The compound may inhibit signaling pathways responsible for receiving and processing proliferation signals, thereby preventing the cell from entering DNA replication and causing it to remain in the G0/G1 phase. mTOR is a protein kinase that regulates processes such as protein synthesis, cell growth, and proliferation by sensing external cues, including nutrients, growth factors, and cellular energy status.^27^ During the critical G1-to-S phase transition, mTOR activity is essential. Drugs targeting mTOR can arrest the cell cycle in the G1 phase and are employed in cancer therapy as well as immunosuppression in organ transplantation.^27^ By inhibiting mTOR, these drugs effectively stop cell cycle progression.

### 2.3 İn Silico Analysis

#### 2.3.1 Molecular Docking

To investigate the molecular mechanism underlying the interaction of our selected 20 compounds with the mammalian target of rapamycin (mTOR), we employed the mTOR complex with ATP-competitive inhibitor PP242 (PDB ID: 4JT5) as the reference structure.^28,29^ The 20 compounds were docked with 4JT5, and significant nonbonding interactions were observed, indicating the potential thermal stability of the resulting complexes. The binding energy values for these interactions ranged from – 6.11 to – 8.23 kcal/mol.

For each compound, ten docking configurations were analyzed. The most stable configuration, characterized by the lowest binding free energy and highest entropy value, is presented in Table 5 and Figure 13. Among the tested molecules, Compound 20 exhibited the greatest stability with a binding energy of – 8.23 kcal/mol. This enhanced stability is attributed to the hydrogen bond donor capability of the NH group, which interacts with the carboxylate moiety of Asp1632. The second most stable compound, Compound 16 (−7.78 kcal/mol), displayed hydrogen bond-mediated π-interactions (H−π interactions) between the proton donor His1541 and the aromatic π-electron cloud. A similar H−π interaction was also observed between Lys1635 and one of the compound’s aromatic rings.

**Table 5:** Comparative Docking Data of P2X with Other Compounds

**compounds**	**docking score**kcal/mol
P2X	–7.87
COH-1	–6.24
COH-2	–6.75
COH-3	–6.91
COH-4	–7.19
COH-5	–6.98
COH-6	–7.03
COH-7	–7.13
COH- 8	–7.88
COH-9	–6.11
COH-10	–6.73
COH-11	–7.04
COH-12	–7.26
COH-13	–6.94
COH-14	–7.18
COH-15	–7.24
COH-16	–7.78
COH-17	–7.23
COH-18	–7.34
COH-19	–7.65
COH-20	–8.23

**Figure 13:**
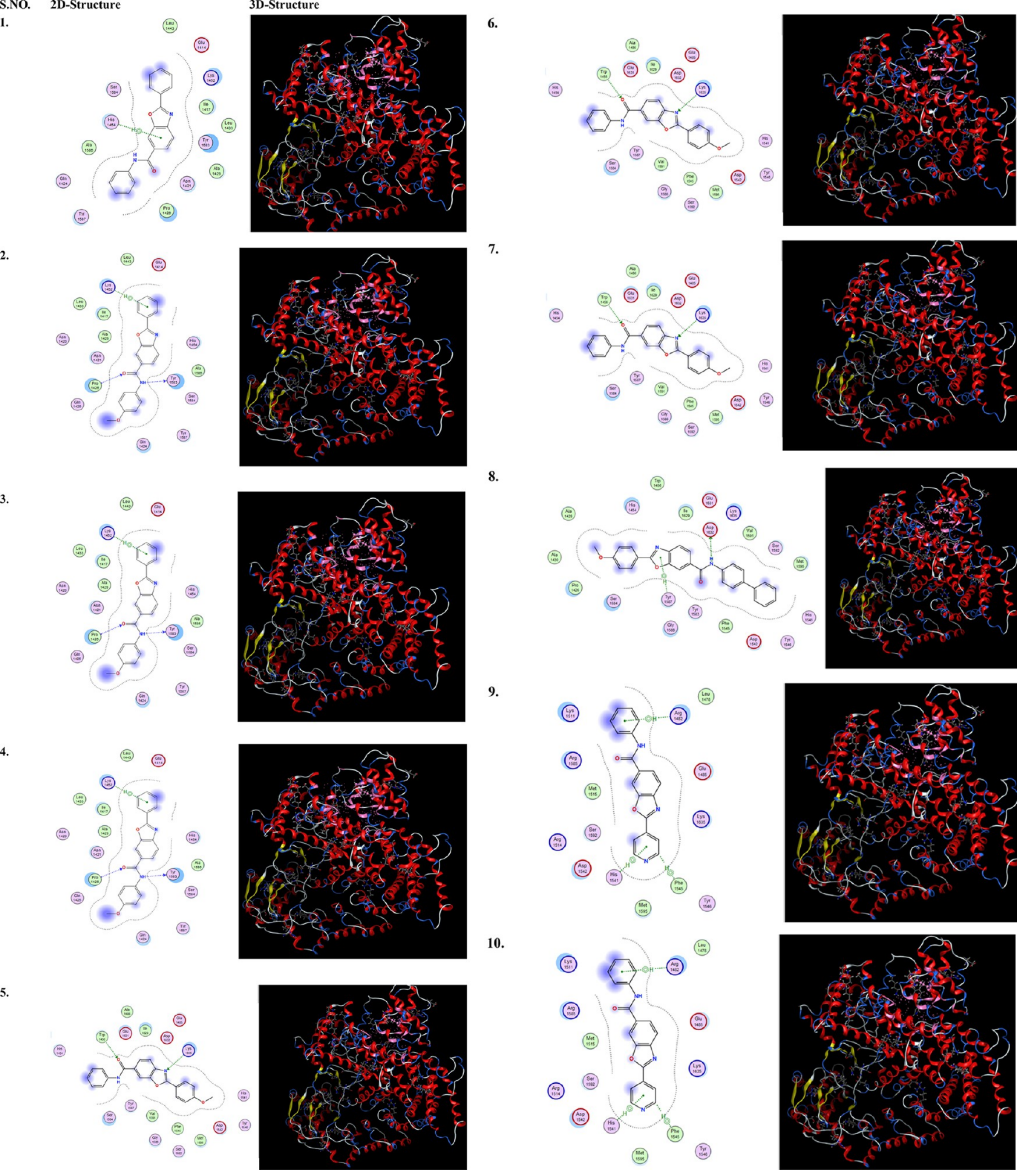

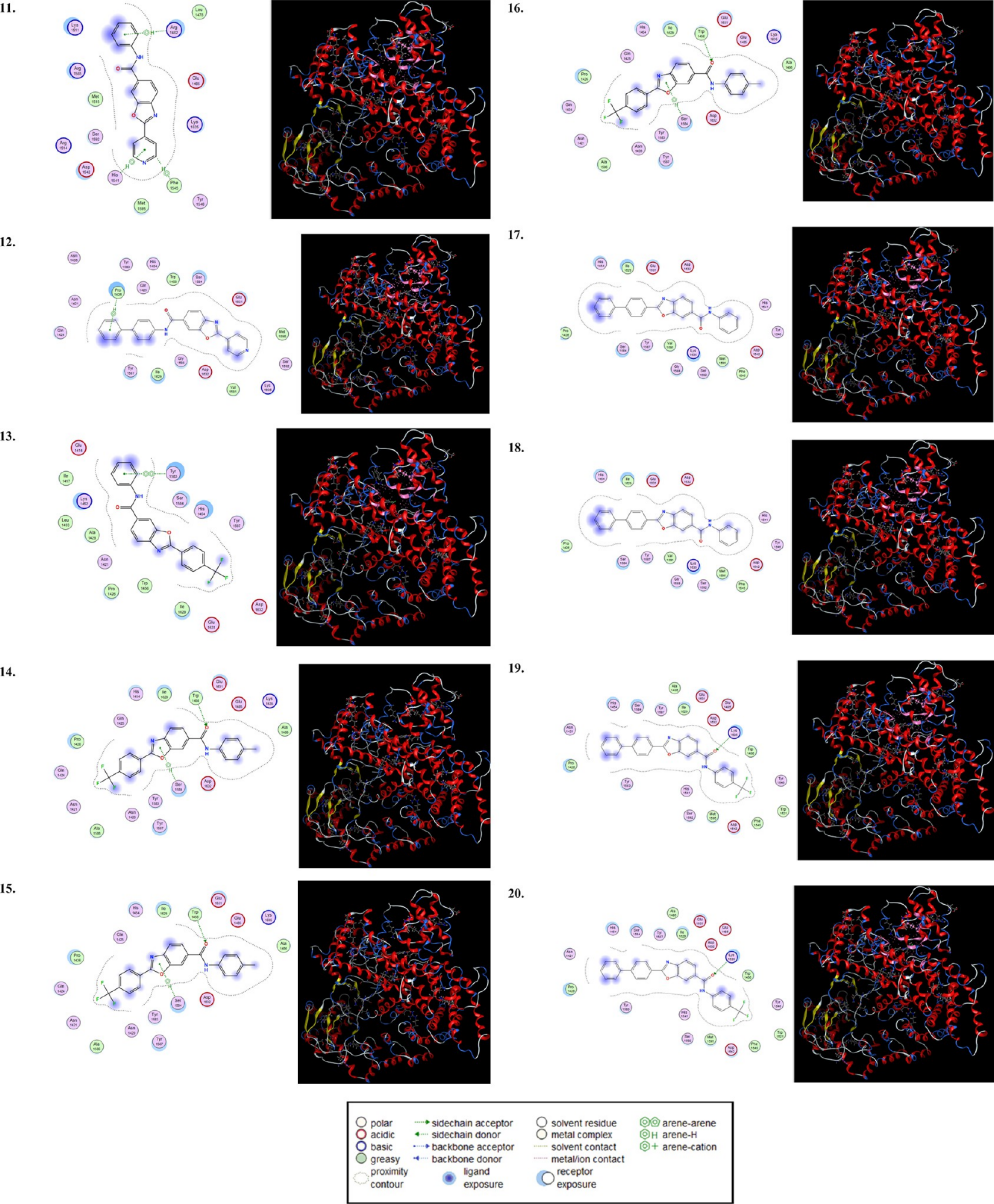
Molecular docking showing PDB ID: 4JT5 with 20 compounds (Left side: 2D and right side: 3D).

To further validate the docking protocol, 4JT5 was redocked with P2X and compared with the docking results of our compounds. Previous studies have reported that P2X forms hydrogen bonds with Asp2195, Val2240, and Gly2238 residues. In our redocking analysis, P2X interacted with IIe2237 and also demonstrated a hydrogen bond-mediated H−π interaction, where IIe2237 acted as a proton donor to the aromatic π-system. Similar pyrrole ring show arene interaction with proton of the Met2345 amino acid. Amino group show backbone donor capability toward the Gly2238 and nitrogen of the pyridine ring have interaction with the Val2240 where Val2240 act as backbone acceptor. Lys2187 interact with Pyrrole ring through cation-arene interaction. The P2X complex displayed a binding free energy of – 7.87 kcal/mol, indicating thermal stability. However, this value was less favorable than that observed for Compound 20, suggesting superior binding stability for our top compound (Figure 14).

**Figure 14:**
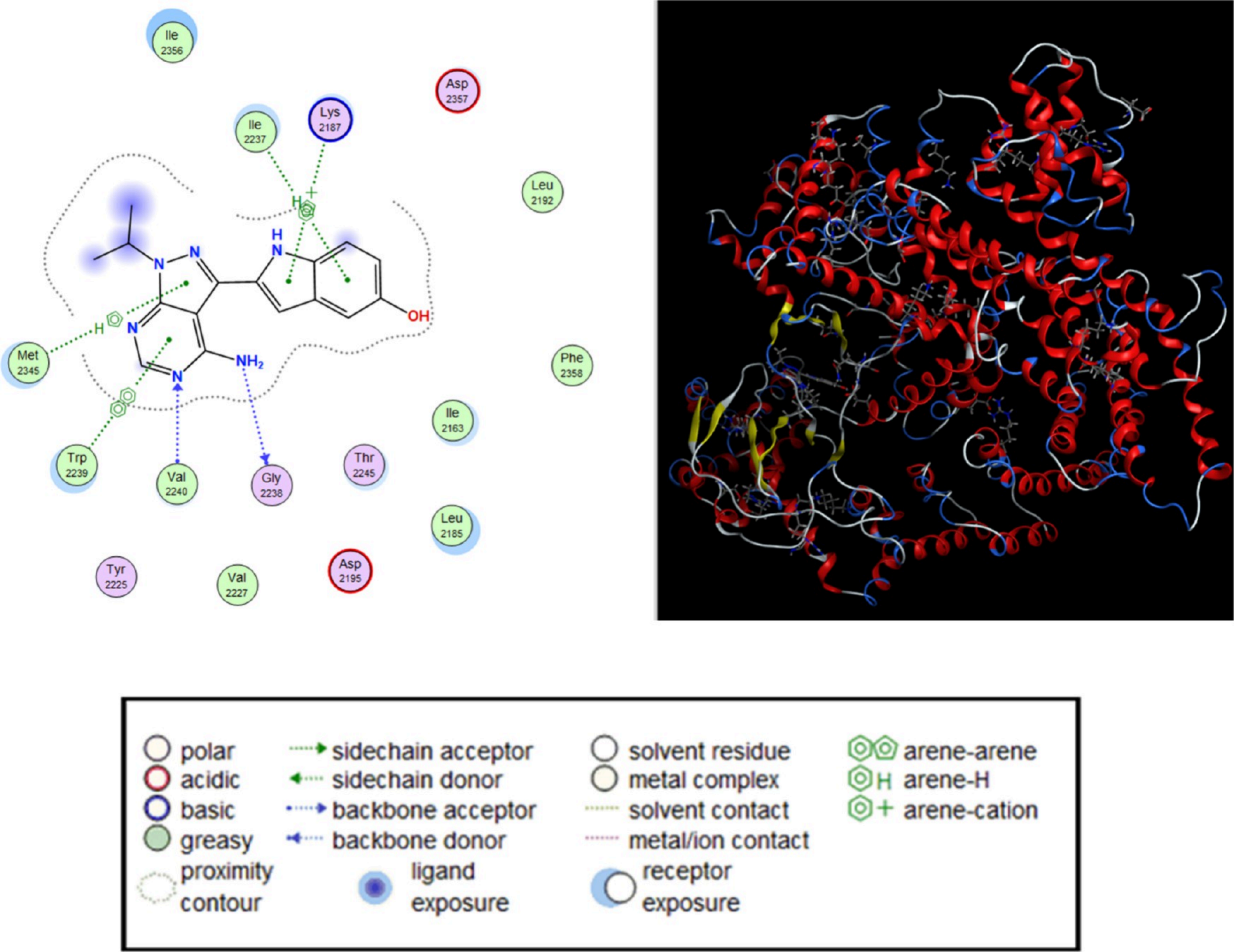
Molecular docking showing PDB ID: 4JT5 with P2X­(Left side: 2D and right side: 3D).

#### 2.3.2 Molecular Dynamic Simulation

GROMACS molecular dynamics (MD) simulations^30,31^ were employed to explore the dynamic behavior and conformational stability of COH-17 and 19 in complex with the mammalian target of rapamycin (mTOR; PDB ID: 4JT5). The respective enzyme–ligand complexes were constructed using relevant force field parameters, solvated in explicit water, and neutralized with counterions. Energy minimization and equilibration were followed by production runs of 100 ns under constant temperature and pressure. Root mean square deviation (RMSD) analyses demonstrated that both complexes exhibited stable trajectories throughout the simulation period, with root-mean-square fluctuation (RMSF) revealing limited flexibility among residues lining the active site. Moreover, the radius of gyration confirmed the persistent compactness of each complex, where COH-17 displayed greater compactness and lower deviation compared to COH-19. Structural fluctuations were broadly comparable between the two complexes (Figures 15–17). Collectively, these results offer detailed mechanistic insights into enzyme–ligand interactions, consistent with prior computational and experimental findings.^32,33^ Nonbonded interaction energies (E_int_) between each ligand and the mTOR protein were evaluated as the sum of Coulombic electrostatic and Lennard–Jones van der Waals components. For COH-17, short-range Coulombic and Lennard–Jones interaction energies were – 0.08 and – 0.31 kcal/mol, respectively, with a total of – 0.39 kcal/mol, reflecting strong ligand–protein binding. Long-range contributions were 180.04 kcal/mol (Coulombic) and 238.33 kcal/mol (Lennard–Jones), culminating in 418.37 kcal/mol total energy. For COH-19, long-range Coulombic and Lennard–Jones terms were 355.49 and 243.47 kcal/mol, yielding 598.98 kcal/mol in total, while the corresponding short-range energies were – 0.45 and – 0.06 kcal/mol, totaling – 0.51 kcal/mol. These energetic profiles further substantiate the robust binding of both compounds to mTOR, with COH-17 displaying a marginally superior structural compactness throughout the simulations.

**Figure 15:**
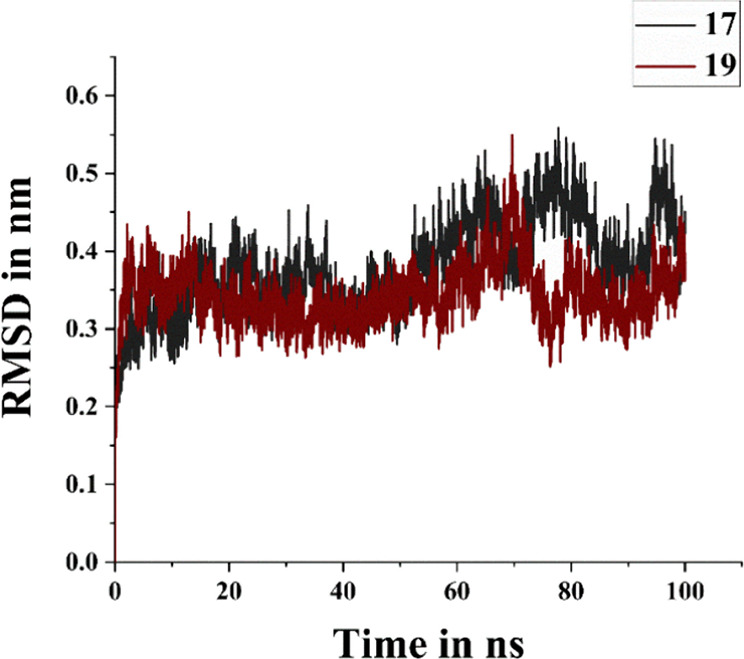
RMSD graph of COH-17 and 19 with 4JT5.

**Figure 16:**
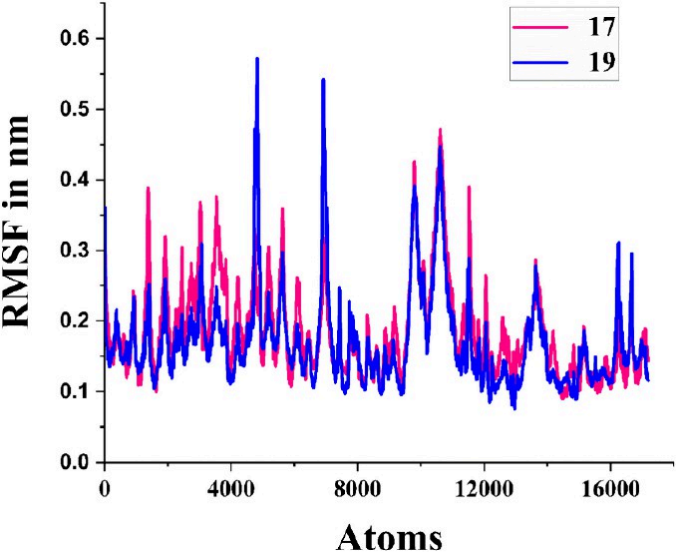
RMSF graph of COH-17 and 19 with 4JT5.

**Figure 17:**
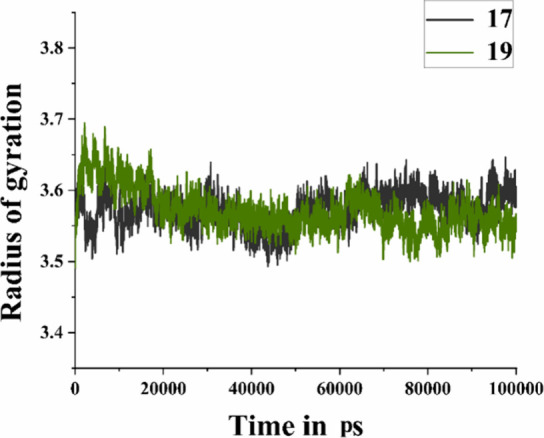
Radius of gyration graph for COH-17 and 19 with 4JT5.

##### 2.3.2.1 Molecular Dynamics Study of COH-17 and 19 with 4JTS

GROMACS molecular dynamics (MD) simulations^30,31^ were employed to explore the dynamic behavior and conformational stability of COH-17 and 19 in complex with the mammalian target of rapamycin (mTOR; PDB ID: 4JT5). The respective enzyme–ligand complexes were constructed using relevant force field parameters, solvated in explicit water, and neutralized with counterions. Energy minimization and equilibration were followed by production runs of 100 ns under constant temperature and pressure. Root mean square deviation (RMSD) analyses demonstrated that both complexes exhibited stable trajectories throughout the simulation period, with root-mean-square fluctuation (RMSF) revealing limited flexibility among residues lining the active site.

The radius of gyration confirmed the persistent compactness of each complex, where COH-17 displayed greater compactness and lower deviation compared to COH-19. Structural fluctuations were broadly comparable between the two complexes (Figure 15).

These results offer detailed mechanistic insights into enzyme–ligand interactions, consistent with prior computational and experimental findings.^34,33^ Nonbonded interaction energies (E_int_) between each ligand and the mTOR protein were evaluated as the sum of Coulombic electrostatic and Lennard–Jones van der Waals components. For COH-17, short-range Coulombic and Lennard–Jones interaction energies were – 0.08 and – 0.31 kcal/mol, respectively, with a total of – 0.39 kcal/mol, reflecting strong ligand–protein binding (Figure 16).

Long-range contributions were 180.04 kcal/mol (Coulombic) and 238.33 kcal/mol (Lennard–Jones), culminating in 418.37 kcal/mol total energy. For COH-19, long-range Coulombic and Lennard–Jones terms were 355.49 and 243.47 kcal/mol, yielding 598.98 kcal/mol in total, while the corresponding short-range energies were – 0.45 and – 0.06 kcal/mol, totaling – 0.51 kcal/mol. These energetic profiles further substantiate the robust binding of both compounds to mTOR, with COH-17 displaying a marginally superior structural compactness throughout the simulations (Figure 17).

#### 2.3.3 ADME Prediction

In the graph created for Brain-Blood Barrier (BBB) permeability, the lipophilicity (WLOGP) and polarity (TPSA) parameters of COH-17 and COH-19 are compared, with the yellow region representing high BBB permeability potential (Figures 18 and 19). With a TPSA value of approximately 50 and a WLOGP value close to the ideal range, COH-17 is located at the edge of this permeability region and exhibits a more balanced profile in terms of its structural properties. In contrast, COH-19’s very high WLOGP value suggests that the compound exhibits an excessively lipophilic character and therefore may have reduced BBB permeability. The fact that neither compound is labeled as a PGP substrate indicates a low risk of active efflux; however, the general pharmacokinetic evaluation shows that COH-17 has a more advantageous profile. The data presented in Table 6 also corroborate these observations from a medicinal chemistry perspective, indicating that although the study does not directly target an indication on the central nervous system, it provides valuable insights into the safety and permeability profile for different patient groups (such as pregnant women and breastfeeding mothers).^44^

**Figure 18:**
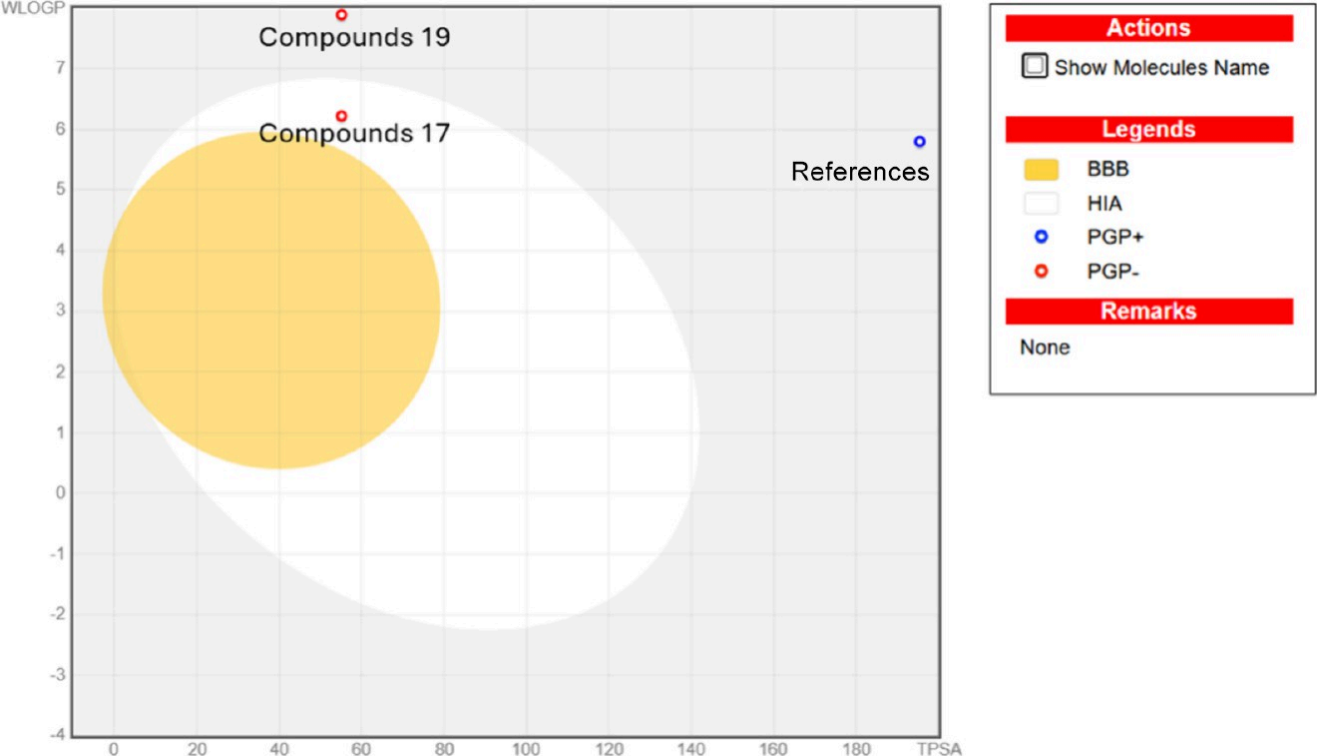
Candidate compounds and reference compound model image using the boiled egg method.

**Figure 19:**
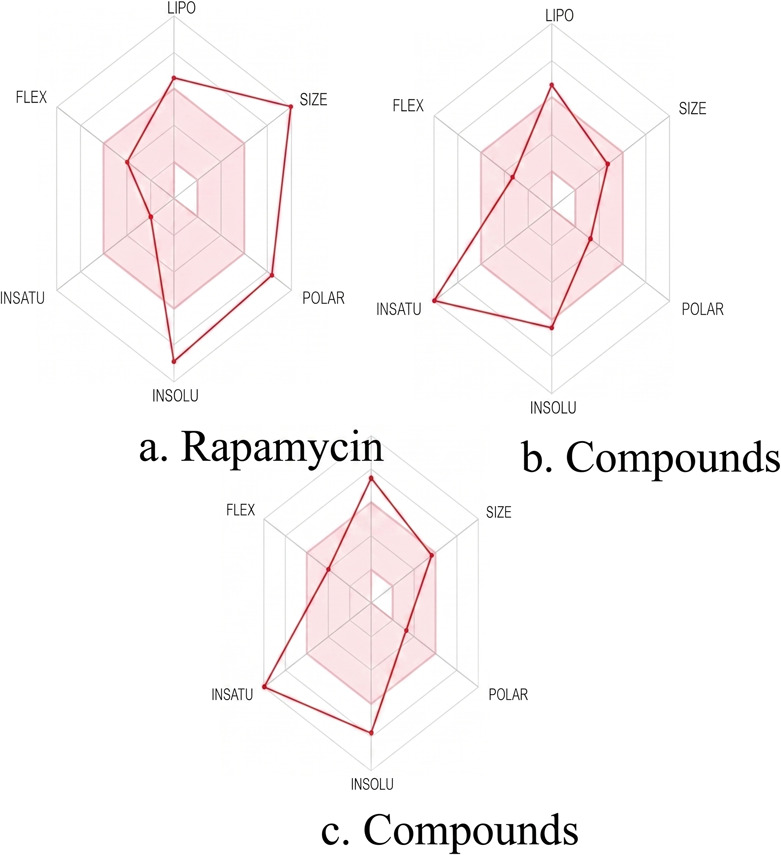
According to ADMET results, the spider web model.

**Table 6:** Some Physicochemical and Drug-Related Parameters for the ADMETox Properties of Compounds

	**TPSA**			**pharmacokinetics**								**druglikeness**				
**compounds name**	Å^2^	**Log*P***	**water solubility class**	**GI**	**BBB**	**Pgb**	**CYP1A2**	**CYP2C19**	**CYP2C9**	**CYP2D6**	**CYP3A4**	**Lipinski**	**Ghose**	**Veber**	**Egan**	**Muegge**
**17**	55.13	3.75	insoluble	high	**-**	**-**	**+**	**+**	**-**	**-**	**+**	**+**	**-**	**+**	**-**	**-**
**19**	55.13	4.02	insoluble	low	**-**	**-**	**-**	**+**	**-**	**-**	**+**	**+**	**-**	**+**	**-**	**-**
**rapamycin**	193.43	4.46	moderately	low	**-**	**+**	**-**	**-**	**-**	**-**	**+**	**-**	**-**	**-**	**-**	**-**

In this study, the anticancer potential of newly synthesized 2-aryl-6-carboxamide benzo­[d]­oxazole derivatives was evaluated, with a particular focus on their mTOR inhibitory activity. The findings demonstrate that these compounds suppress cancer cell proliferation by targeting the PI3K/Akt/mTOR signaling pathway. Notably, the compounds COH-17 and COH-19 exhibited pronounced antiproliferative effects in MCF-7 and MDA-MB-231cells, accompanied by reduced mTOR protein expression, induction of apoptosis, and cell cycle arrest.

These results support the notion that mTOR plays a central role in regulating cellular growth, metabolism, and survival. That inhibition of this pathway represents an effective strategy for cancer therapy. A recent review reported that classical allosteric inhibitors, such as rapamycin and its derivatives (temsirolimus, everolimus, deforolimus), selectively inhibit the mTORC1 complex. In contrast, next-generation ATP-competitive inhibitors can suppress the activities of both mTORC1 and mTORC2.^33^ In this context, the observation that COH-17 and COH-19 reduce mTOR protein levels by more than 80% suggests that these compounds may exert a broad and potent inhibitory effect.

Molecular docking analyses revealed that these compounds bind with high affinity to the ATP-binding pocket of mTOR.^35^ The calculated binding energies were – 7.23 kcal/mol for COH-17 and – 7.65 kcal/mol for COH-19, confirming their strong interactions. Moreover, apoptosis assays indicated that both compounds induce cell death via the mitochondrial (intrinsic) pathway. RT-qPCR and Western blot analyses further demonstrated an increased Bax/Bcl-2 ratio, upregulation of TP53 and PTEN, and downregulation of PI3K and AKT expression. These findings suggest that inhibition of the PI3K/Akt/mTOR pathway occurs through PTEN activation, leading to induction of apoptosis via cell-cycle arrest mediated by p53 activation.^36,37^

Additionally, LDH assays and Annexin V/PI staining confirmed the dose-dependent cytotoxic and apoptotic effects of both compounds. Notably, COH-17 induced high levels of apoptosis at moderate and high doses and caused G0/G1 phase arrest. This effect aligns with the mechanism of mTOR inhibitors, which block the G1 phase by reducing the phosphorylation of Cyclin D1 and S6*K*/4E-BP1.^38,39^ Furthermore, the absence of significant toxicity in MCF-10A supports the selective anticancer activity of these compounds.

## 3 Conclusions

The newly synthesized 2-aryl-6-carboxamide derivatives demonstrated significant anticancer potential against breast cancer cell lines, particularly MCF-7, by inhibiting the mTOR pathway. Computational analyses supported these experimental findings, revealing strong binding affinities and stable interactions between the lead compounds and the mTOR active site. Among the tested molecules, COH-17 and COH-19 emerged as the most potent candidates, exhibiting pronounced cytotoxic effects, disruption of cell membrane integrity, and induction of apoptosis.

Both phenotypic and molecular assays confirmed that these compounds induced apoptotic cell death, characterized by chromatin condensation, nuclear fragmentation, and the upregulation of pro-apoptotic genes such as Bax, Caspase-3, and TP53, accompanied by the downregulation of Bcl-2. Furthermore, cell cycle analysis revealed that treatment with COH-17 and COH-19 caused G0/G1 phase arrest, thereby suppressing cell proliferation.

Overall, these findings highlight COH-17 and COH-19 as promising lead compounds for the development of novel mTOR-targeted anticancer agents. Further in vivo studies and pharmacokinetic evaluations are warranted to confirm their therapeutic potential and optimize their drug-like properties.

## 4 Experimental Section

### 4.1 Chemistry

#### 4.1.1 Synthesis of the Compound (b)

4-Amino-3-hydroxybenzoic acid (1 mmol) was dissolved in 10 mL of methanol. A few drops of concentrated sulfuric acid were added as a catalyst, and the reaction mixture was refluxed for 12 h. The progress of the reaction was monitored by thin-layer chromatography (TLC). Upon completion, the mixture was allowed to cool to room temperature and subsequently neutralized to pH 7.5 with 50 mL of water and 1 N sodium bicarbonate (NaHCO_3_). The aqueous layer was extracted three times with 15 mL portions of ethyl acetate. The combined organic extracts were separated, and the solvent was removed under reduced pressure. The crude product was purified by column chromatography on silica gel using *n*-hexane/ethyl acetate (5:1, v/v) as the eluent to afford the pure target compound.^45^

#### 4.1.2 Synthesis of the Compounds (c and d)

In the first step, methyl 4-amino-3-hydroxybenzoate (5 mmol) was dissolved in 10 mL of ethanol. A solution of the corresponding aromatic aldehydes (benzaldehyde, 4-pyridinecarboxaldehyde, 4-biphenylcarboxaldehyde, 2-naphthaldehyde, or 2-thiophenecarboxaldehyde; 7 mmol) in 5 mL of ethanol was added dropwise to the reaction mixture. The reaction was stirred at room temperature for 6 h. The progress of the reaction was monitored by thin-layer chromatography (TLC), and upon complete conversion of the starting material to the Schiff base, the reaction was terminated. The crude products were extracted with ethyl acetate (3 × 15 mL) and water (30 mL), and crystallized from methanol. Ethanol was removed under reduced pressure, and the resulting crude Schiff bases were used directly in the next step without further purification. In the subsequent step, the obtained Schiff bases were dissolved in 10 mL of DMF, and a catalytic amount of sodium cyanide (NaCN) was added. The reaction mixture was stirred in open air at room temperature for 12 h, after which a catalytic amount of hydrogen peroxide (H_2_O_2_) was added and stirring was continued for an additional 1 h. The progress of the reaction was monitored by TLC. Upon completion, the reaction mixture was extracted with saturated brine (50 mL) and ethyl acetate (3 × 30 mL). The combined organic layers were dried over anhydrous sodium sulfate, and the solvent was removed under reduced pressure. The crude products were purified by column chromatography using an appropriate ratio of ethyl acetate/*n*-hexane as the eluent to afford the pure target compounds.^46,47^

#### 4.1.3 Synthesis of the Final Compounds (1–20)

In the final step, methyl 2-(aryl-substituted)­benzoxazole-6-carboxylate derivatives (0.5 mmol) were dissolved in 4 mL of dichloromethane (DCM). To this solution, the corresponding p-substituted aniline derivatives (1.2 mmol) were added. The reaction mixture was stirred at room temperature for 1 h, followed by the addition of triethylamine (TEA, 1.2 mmol). The mixture was stirred at room temperature for an additional 30 min, after which a catalytic amount of aluminum chloride (AlCl_3_) was added. The reaction was allowed to stir overnight at room temperature. Progress of the reaction was monitored by thin-layer chromatography (TLC), and upon complete conversion of the starting material to the product, the reaction was terminated. The resulting crude products were extracted with dichloromethane (3 × 15 mL) and water (30 mL). The combined organic layers were dried over anhydrous magnesium sulfate (MgSO_4_) and filtered. The solvent was removed under reduced pressure to yield a gel-like crude product. The crude compounds were washed with cyclohexane and purified by column chromatography on silica gel using an appropriate ratio of *n*-hexane/ethyl acetate as the eluent to afford the pure final products.^48^

The structures of the synthesized compounds, the results of the structural determination, and the spectra are presented below.

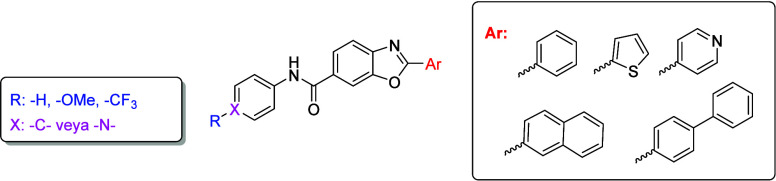



#### 4.1.4 *N*,2-Diphenylbenzo­[*d*]­oxazole-6-carboxamide (1)

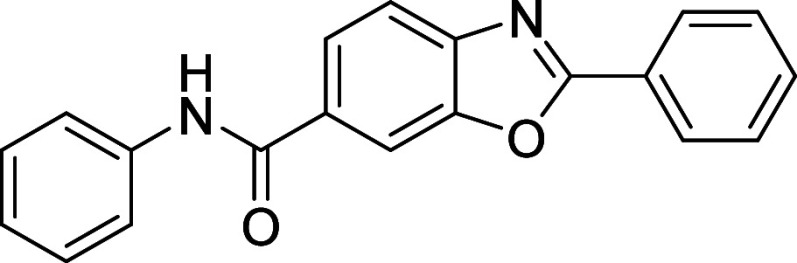



Light orange powder, Mp 226–227 °C, Yield 73%. FT-IR υ_max_ (cm^–1^) 3286.86 (N–H), 3061.97 (aromatic C–H), 2915.71 (aliphatic C–H), 1668.98 (C = O), 1535.88, 1484.89, 1441.35 (aromatic C = C), 875.16 (C–O), 1125.88 (C–N).^1^H NMR (400 MHz, *d*_*6*_-DMSO) δ 10.36 (s, 1H, -NH), 8.38 (d, J= 0.8 Hz, 1H, Ar–H), 8.23 (dd, J= 1.3 Hz, J= 7.8 Hz, 2H, Ar–H), 8.03 (dd, J= 1.3 Hz, J= 8.3 Hz, 1H, Ar–H), 7.93 (d, J= 8.3 Hz, 1H, Ar–H), 7.80 (d, J= 7.8 Hz, 2H, Ar–H), 7.70–7.61 (m, 3H, Ar–H), 7.36 (t, J= 7.8 Hz, 2H, Ar–H), 7.10 (t, J= 7.4 Hz, 1H, Ar–H). ^13^C NMR (100 MHz, *d*_*6*_-DMSO) δ 165.2, 164.8, 150.4, 144.6, 139.6, 133.0, 132.7, 129.9, 129.1, 128.0, 126.5, 125.4, 124.2, 120.9, 119.9, 111.0. HRMS [M + H]; Calculated for C_20_H_15_N_2_O_2_: 315.1128, Found: 315.1125.

#### 4.1.5 *N*-(4-Methoxyphenyl)-2-phenylbenzo­[*d*]­oxazole-6-carboxamide (2)

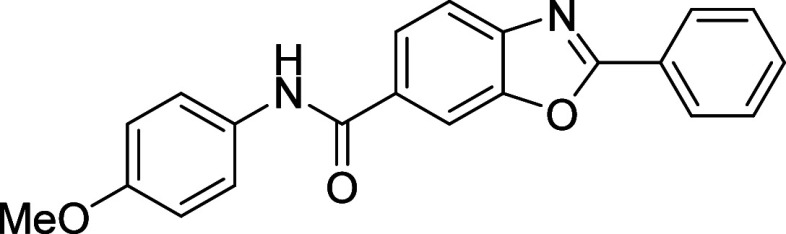



Pink powder, Mp 240–241 °C, Yield 81%. FT-IR υ_max_ (cm^–1^) 3293.05 (N–H), 3001.21 (aromatic C–H), 2974.35 (aliphatic C–H), 1598.21 (C = O), 1535.13, 1490.08, 1436.62 (aromatic C = C), 837.33 (C–O), 1119.43 (C–N). ^1^H NMR (400 MHz, *d*_*6*_-DMSO) δ 10.24 (s, 1H, -NH), 8.36 (s, 1H, Ar–H), 8.23 (d, J= 6.5 Hz, 2H, Ar–H), 8.02 (d, J= 8.3 Hz, 1H, Ar–H), 7.91 (d, J= 8.3 Hz, 1H, Ar–H), 7.75–7.57 (m, 5H, Ar–H), 6.97–6.89 (m, AA’BB’ system, 2H, Ar–H), 3.74 (s, 3H, -OMe). ^13^C NMR (100 MHz, *d*_*6*_-DMSO) δ 164.7, 156.1, 150.4, 144.4, 132.9, 132.8, 132.7, 129.9, 128.0, 126.6, 125.2, 122.5, 119.9, 119.3, 114.2, 110.8, 55.7. HRMS [M + H]; Calculated for C_21_H_14_F_3_N_2_O_2_: 383.1001, Found: 383.1002.

#### 4.1.6 2-Phenyl-*N*-(4-(trifluoromethyl)­phenyl)­benzo­[*d*]­oxazole-6-carboxamide (3)

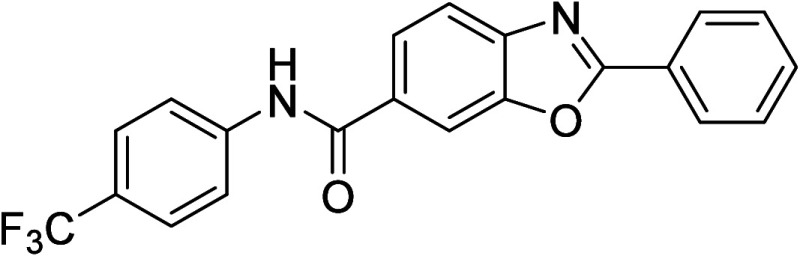



Pink powder, Mp 230–231 °C, Yield 70%. FT-IR υ_max_ (cm^–1^) 3345.96 (N–H), 3061.91 (aromatic C–H), 2984.08 (aliphatic C–H), 1647.40 (C = O), 1519.92, 1483.15, 1446.73 (aromatic C = C), 819.04 (C–O), 1030.82 (C–N).^1^H NMR (400 MHz, *d*_*6*_-DMSO) δ 10.69 (s, 1H, -NH), 8.43–8.40 (m, 1H, Ar–H), 8.27–8.22 (m, 2H, Ar–H), 8.06–8.01 (m, 3H, Ar–H), 7.95 (dd, J= 0.5 Hz, J= 8.3 Hz, 1H, Ar–H), 7.77–7.71 (m, AA’BB’ system, 2H, Ar–H), 7.69–7.61 (m, 3H, Ar–H). ^13^C NMR (100 MHz, *d*_*6*_-DMSO) δ 165.9, 164.9, 150.3, 144.8, 143.2, 132.9, 132.0, 129.9, 128.0, 126.4, 126.3 (quasi q, J= 3.2 Hz), 125.5, 124.2, 123.9, 120.5, 119.9, 111.1. HRMS [M + H]; Calculated for C_21_H_17_N_2_O_3_: 345.1233, Found: 345.1232.

#### 4.1.7 2-Phenyl-*N*-(pyridin-4-yl)­benzo­[*d*]­oxazole-6-carboxamide (4)

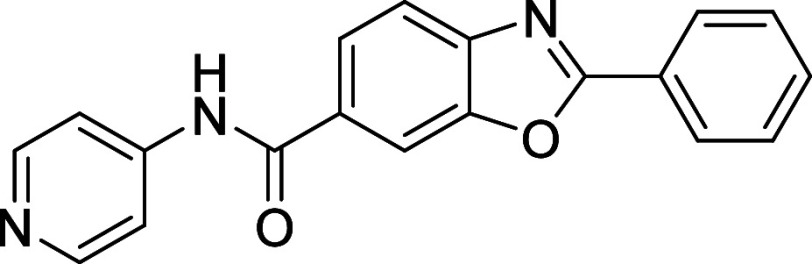



Light yellow powder, Mp 247–248 °C, Yield 74%. FT-IR υ_max_ (cm^–1^) 3369.29 (N–H), 3031.50 (aromatic C–H), 2920.57 (aliphatic C–H), 1661.94 (C = O), 1583.55, 1549.00, 1503.93 (aromatic C = C), 815.29 (C–O), 1262.55 (C–N).^1^H NMR (400 MHz, *d*_*6*_-DMSO) δ 10.69 (bs, 1H, -NH), 8.48 (d, J= 4.6 Hz, 2H, Ar–H), 8.42–8.39 (m, 1H, Ar–H), 8.26–8.21 (m, 2H, Ar–H), 8.03 (dd, J= 1.6 Hz, J= 8.3 Hz, 1H, Ar–H), 7.95 (dd, J= 0.4 Hz, J= 8.3 Hz, 1H, Ar–H), 7.83–7.77 (m, 2H, Ar–H), 7.70–7.62 (m, 3H, Ar–H). ^13^C NMR (100 MHz, *d*_*6*_-DMSO) δ 166.1, 165.0, 150.8, 150.3, 146.3, 144.9, 133.0, 131.7, 129.9, 128.1, 126.4, 125.5, 120.0, 114.4, 111.3. HRMS [M + H]; Calculated for C_19_H_14_N_3_O_2_: 316.1080, Found: 316.1080.

#### 4.1.8 *N*-Phenyl-2-(thiophen-2-yl)­benzo­[*d*]­oxazole-6-carboxamide (5)

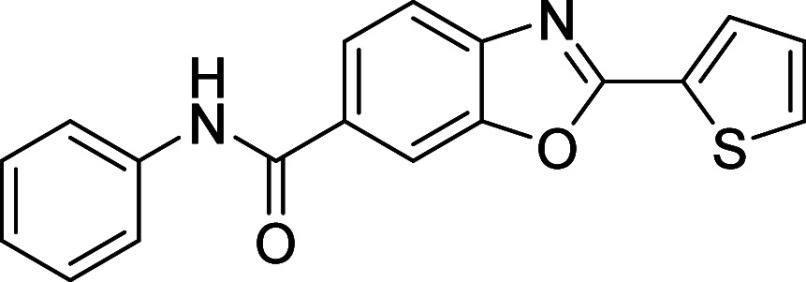



Light yellow powder, Mp 216–217 °C, Yield 90%. FT-IR υ_max_ (cm^–1^) 3272.89 (N–H), 3016.17 (aromatic C–H), 2954.53 (aliphatic C–H), 11650.46 (C = O), 1592.53, 1565.37, 1534.31 (aromatic C = C), 750.02 (C–O), 1061.79 (C–N). ^1^H NMR (400 MHz, *d*_*6*_-DMSO) δ 10.34 (s, 1H, -NH), 8.33 (d, J= 1.17 Hz, 1H, Ar–H), 8.04–7.98 (m, 3H, Ar–H), 7.86 (d, J= 8.3 Hz, 1H, Ar–H), 7.81–7.75 (m, 2H, Ar–H), 7.39–7.30 (m, 3H, Ar–H), 7.13–7.07 (m, 1H, Ar–H). ^13^C NMR (100 MHz, *d*_*6*_-DMSO) δ 165.2, 160.8, 150.1, 144.5, 139.5, 133.4, 132.5, 131.9, 129.6, 129.2, 128.5, 125.4, 124.2, 120.9, 119.6, 110.7. HRMS [M + H]; Calculated for C_18_H_13_N_2_O_2_S: 321.0692, Found: 321.0692.

#### 4.1.9 2-(Thiophen-2-yl)-*N*-(4-(trifluoromethyl)­phenyl)­benzo­[*d*]­oxazole-6-carboxamide (6)

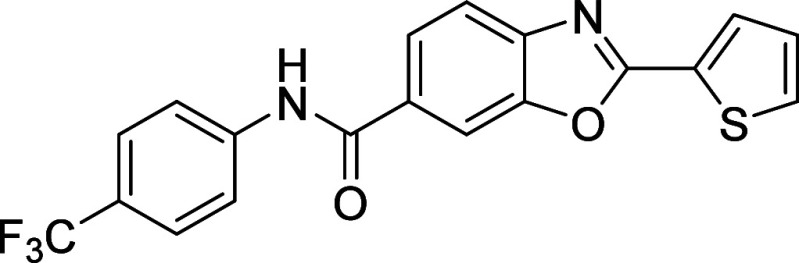



Light yellow powder, Mp 263–264 °C, Yield 94%. FT-IR υ_max_ (cm^–1^) 3272.89 (N–H), 3039.85 (aromatic C–H), 2943.80 (aliphatic C–H), 1652.93 (C = O), 1596.21, 1565.45, 1523,75 (aromatic C = C), 834.06 (C–O), 1012.89 (C–N). ^1^H NMR (400 MHz, *d*_*6*_-DMSO) δ 10.66 (s, 1H, -NH), 8.35 (d, J= 1.6 Hz, 1H, Ar–H), 8.08–7.97 (m, 5H, Ar–H), 7.88 (d, J= 8.3 Hz, 1H, Ar–H), 7.76–7.70 (m, AA’BB’ system, 2H, A-H), 7.35–7.31 (m, 1H, Ar–H). ^13^C NMR (100 MHz, *d*_*6*_-DMSO) δ 165.6, 161.0, 150.0, 144.8, 143.2, 133.4, 131.9, 129.6, 128.5, 126.6, 126.4 (quasi q, J= 3.2 Hz), 125.6, 120.5, 119.6, 111.0. HRMS [M + H]; Calculated for C_19_H_12_F_3_N_2_O_2_S: 389.0566, Found: 389.0568.

#### 4.1.10 *N*-(4-Methoxyphenylphenyl)-2-(thiophen-2-yl)­benzo­[*d*]­oxazole-6-carboxamide (7)

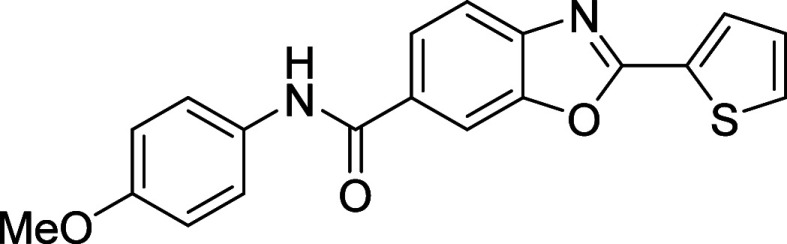



Light yellow powder, Mp 219–220 °C, Yield 94%. FT-IR υ_max_ (cm^–1^) 3336.45 (N–H), 3039.22 (aromatic C–H), 2953.73 (aliphatic C–H), 1645.92 (C = O), 1596.74, 1566.23, 1520.29 (aromatic C = C), 819.05 (C–O), 1024.05 (C–N).^1^H NMR (400 MHz, *d*_*6*_-DMSO) δ 10.22 (s, 1H, -NH), 8.31 (d, J= 1.0 Hz, 1H, Ar–H), 8.04–7.96 (m, 3H, Ar–H), 7.84 (d, J= 8.3 Hz, 1H, Ar–H), 7.72–7.64 (m, AA’BB’ system, 2H, Ar–H), 7.32 (dd, J= 3.8 Hz, J= 4.9 Hz, 1H, Ar–H), 6.96–6.89 (m, AA’BB’ system, 2H, Ar–H), 3.73 (s, 3H, -OMe). ^13^C NMR (100 MHz, *d*_*6*_-DMSO) δ 164.7, 160.7, 156.0, 150.1, 144.4, 133.2, 132.6, 131.8, 129.4, 128.5, 125.3, 122.5, 122.3, 119.6, 114.2, 110.6, 55.5. HRMS [M + H]; Calculated for C_19_H_15_N_2_O_3_S: 351.0797, Found: 351.0796.

#### 4.1.11 *N*-(Pyridin-4-yl)-2-(thiophen-2-yl)­benzo­[*d*]­oxazole-6-carboxamide (8)

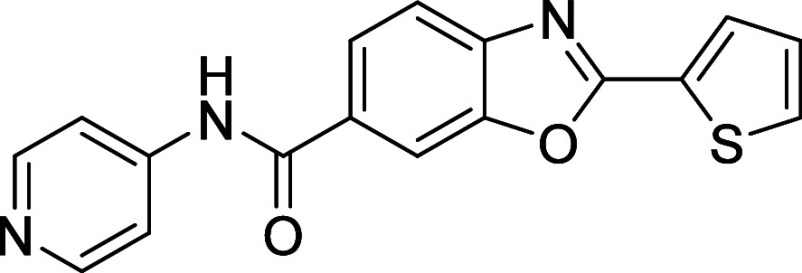



Light pink powder, Mp 108–109 °C, Yield 79%. FT-IR υ_max_ (cm^–1^) 3252.53 (N–H), 3043.71 (aromatic C–H), 2646.03 (aliphatic C–H), 1685.72 (C = O), 1595.26, 1545.95, 1520.51 (aromatic C = C), 825.71 (C–O), 1030.25 (C–N). ^1^H NMR (400 MHz, *d*_*6*_-DMSO) δ 10.67 (s, 1H, -NH), 8.58–8.38 (m, 2H, Ar–H), 8.32 (s, 1H, Ar–H), 8.05–7.96 (m, 3H, Ar–H), 7.86 (d, J= 8.3 Hz, 1H, Ar–H), 7.79–7.73 (m, 2H, Ar–H), 7.34–7.29 (m, 1H, Ar–H). ^13^C NMR (100 MHz, *d*_*6*_-DMSO) δ 166.0, 161.0, 150.7, 150.0, 146.3, 144.9, 133.4, 131.9, 131.5, 129.5, 128.4, 125.6, 119.6, 114.5, 111.0. HRMS [M + H]; Calculated for C_17_H_12_N_3_O_2_S: 322.0644, Found: 322.0643.

#### 4.1.12 *N*-Phenyl-2-(pyridin-4-yl)­benzo­[*d*]­oxazole-6-carboxamide (9)

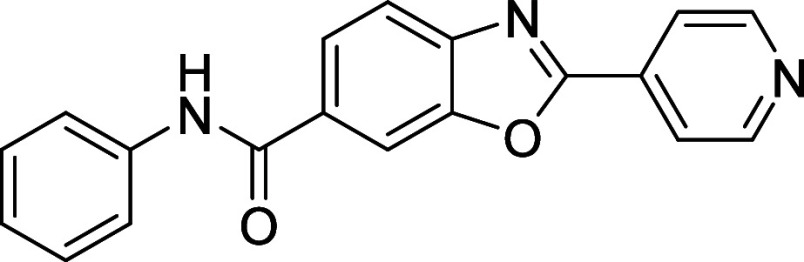



Light red powder, Mp 272–273 °C, Yield 74%. FT-IR υ_max_ (cm^–1^) 3259.88 (N–H), 3036.30 (aromatic C–H), 2672.20 (aliphatic C–H), 1642.47 (C = O), 1595.33, 1531.02, 1479.24 (aromatic C = C), 840.18 (C–O), 1058.37 (C–N). ^1^H NMR (400 MHz, *d*_*6*_-DMSO) δ 10.40 (bs, 1H, -NH), 8.90–8.84 (m, 2H, Ar–H), 8.44 (s, 1H, Ar–H), 8.15–8.11 (m, 2H, Ar–H), 8.07 (dd, J= 1.1 Hz, J= 8.4 Hz, 1H, Ar–H), 8.01 (d, J= 8.4 Hz, 1H, Ar–H), 7.82–7.77 (m, 2H, Ar–H), 7.36 (t, J= 7.8 Hz, 2H, Ar–H), 7.14–7.08 (m, 1H, Ar–H). ^13^C NMR (100 MHz, *d*_*6*_-DMSO) δ 165.0, 162.8, 151.4, 150.5, 144.1, 139.4, 133.6, 133.5, 129.1, 125.6, 124.3, 121.4, 120.8, 120.6, 111.3. HRMS [M + H]; Calculated for C_19_H_14_N_3_O_2_: 316.1080, Found: 316.1081.

#### 4.1.13 *N*-(4-Methoxyphenyl)-2-(pyridin-4-yl)­benzo­[*d*]­oxazole-6-carboxamide (10)

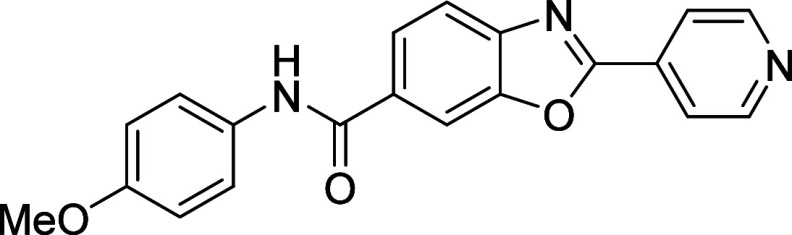



Light pink powder, Mp 300 °C decomp., Yield 85%. FT-IR υ_max_ (cm^–1^) 3376.59 (N–H), 3037.01 (aromatic C–H), 2923.27 (aliphatic C–H), 1650.65 (C = O), 1531.13, 1481.51, 1430.36 (aromatic C = C), 835.06 (C–O), 1071.88 (C–N). ^1^H NMR (400 MHz, *d*_*6*_-DMSO) δ 10.72 (bs, 1H, -NH), 8.89–8.84 (m, 2H, Ar–H), 8.46–8.43 (m, 2H, Ar–H), 8.14–8.10 (m, 2H, Ar–H), 8.07 (dd, J= 1.6 Hz, J= 8.4 Hz, 1H, Ar–H), 8.05–7.99 (m, 3H, Ar–H), 7.76–7.70 (m, AA’BB’ system, 2H, Ar–H). ^13^C NMR (100 MHz, *d*_*6*_-DMSO) δ 165.5, 162.9, 151.4, 150.5, 144.3, 143.2, 143.1, 133.6, 133.0, 126.4 (q, J= 3.7 Hz), 125.8, 124.0, 121.5, 120.7, 120.6, 111.5. HRMS [M + H]; Calculated for C_20_H_13_F_3_N_3_O_2_: 384.0954, Found: 384.0955.

#### 4.1.14 2-(Pyridin-4-yl)-*N*-(4-(trifluoromethyl)­phenyl)­benzo­[*d*]­oxazole-6-carboxamide (11)

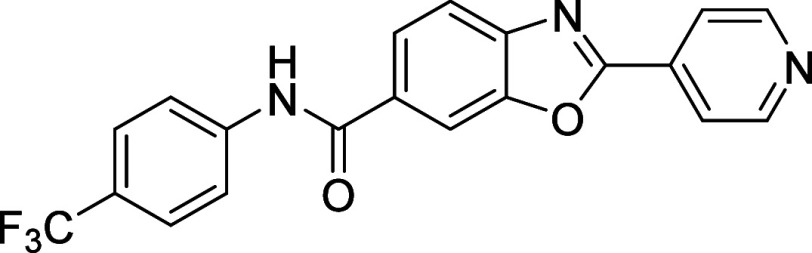



Light pink powder, Mp 240–241 °C, Yield 93%. FT-IR υ_max_ (cm^–1^) 3269.26 (N–H), 3002.37 (aromatic C–H), 2951.30 (aliphatic C–H), 1642.41 (C = O), 1534.47, 1513.22 (aromatic C = C), 816.28 (C–O), 1032.69 (C–N). ^1^H NMR (400 MHz, *d*_*6*_-DMSO) δ 10.27 (bs, 1H, -NH), 8.90–8.83 (m, 2H, Ar–H), 8.41 (d, J= 1.2 Hz, 1H, Ar–H), 8.15–8.10 (m, 2H, Ar–H), 8.06 (dd, J= 1.2 Hz, J= 8.4 Hz, 1H, Ar–H), 7.99 (d, J= 8.4 Hz, 1H, Ar–H), 7.71–7.67 (m, AA’BB’ system, 2H, Ar–H), 6.95–6.91 (m, AA’BB’ system, 2H, Ar–H), 3.74 (s, 3H, -OMe). ^13^C NMR (100 MHz, *d*_*6*_-DMSO) δ 164.5, 162.7, 156.1, 151.4, 150.5, 143.9, 133.7, 133.6, 132.5, 125.5, 122.4, 121.4, 120.5, 114.2, 111.1, 55.6. HRMS [M + H]; Calculated for C_20_H_16_N_3_O_3_: 346.1186, Found: 346.1186.

#### 4.1.15 *N*,2-Di­(pyridin-4-yl)­benzo­[*d*]­oxazole-6-carboxamide (12)

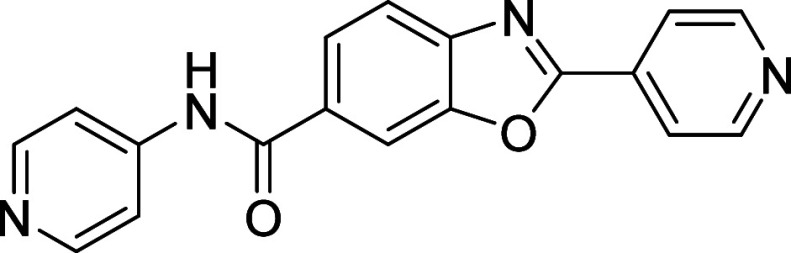



Dark pink powder, Mp 246–247 °C, Yield 70%. FT-IR υ_max_ (cm^–1^) 3241.40 (N–H), 3035.60 (aromatic C–H), 2993.79 (aliphatic C–H), 1593.71 (C = O), 1514.08, 1482.33 (aromatic C = C), 829.28 (C–O), 1057.07 (C–N).^1^H NMR (400 MHz, *d*_*6*_-DMSO) δ 10.71 (s, 1H, -NH), 8.87–8.83 (m, 2H, Ar–H), 8.51–8.44 (m, 2H, Ar–H), 8.42 (dd, J= 0.6 Hz, J = 1.6 Hz, 1H, Ar–H), 8.12–8.08 (m, 2H, Ar–H), 8.05 (dd, J= 1.6 Hz, J= 8.4 Hz, 1H, Ar–H), 8.00 (dd, J= 0.6 Hz, J= 8.4 Hz, 1H, Ar–H), 7.81–7.76 (m, 2H, Ar–H). ^13^C NMR (100 MHz, *d*_*6*_-DMSO) δ 165.9, 163.0, 151.4, 150.8, 150.4, 146.3, 144.4, 133.5, 132.7, 125.8, 121.4, 120.6, 114.5, 111.6. HRMS [M + H]; Calculated for C_18_H_13_N_4_O_2_: 317.1033, Found: 317.1033.

#### 4.1.16 2-(Naphthalen-2-yl)-*N*-phenylbenzo­[*d*]­oxazole-6-carboxamide (13)

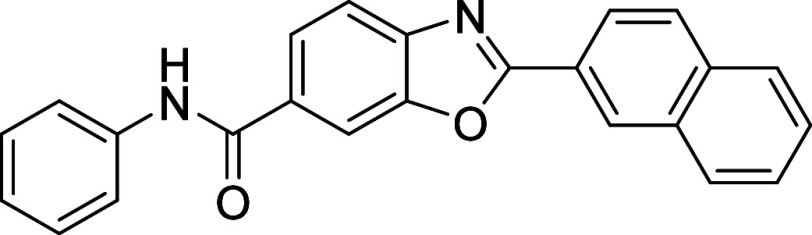



Light yellow powder, Mp 255–256 °C, Yield 92%. FT-IR υ_max_ (cm^–1^) 3383.68 (N–H), 3053.50 (aromatic C–H), 2908.01 (aliphatic C–H), 1647.48 (C = O), 1553.01, 1526.28 (aromatic C = C), 747.74 (C–O), 1028.17 (C–N). 1H NMR (400 MHz, *d6*-DMSO) δ 10.39 (bs, 1H, -NH), 8.88 (s, 1H, Ar–H), 8.40 (s, 1H, Ar–H), 8.29–8.14 (m, 3H, Ar–H), 8.08–8.00 (m, 2H, Ar–H), 7.98–7.93 (m, 1H, Ar–H), 7.81 (d, J= 7.6 Hz, 2H, Ar–H), 7.70–7.61 (m, 2H, Ar–H), 7.37 (t, J= 7.6 Hz, 2H, Ar–H), 7.11 (t, J= 7.2 Hz, 1H, Ar–H). 13C NMR (100 MHz, *d6*-DMSO) δ 165.2, 164.9, 150.5, 144.6, 139.5, 134.9, 132.9, 132.7, 129.6, 129.5, 129.1, 128.9, 128.7, 128.3, 127.8, 125.3, 124.2, 124.1, 123.8, 120.9, 119.9, 110.9. HRMS [M + H]; Calculated for C24H17N2O2:365.1284, Found: 365.1283.

#### 4.1.17 2-(Naphthalen-2-yl)-*N*-(4-(trifluoromethyl)­phenyl)­benzo­[*d*]­oxazole-6-carboxamide (14)

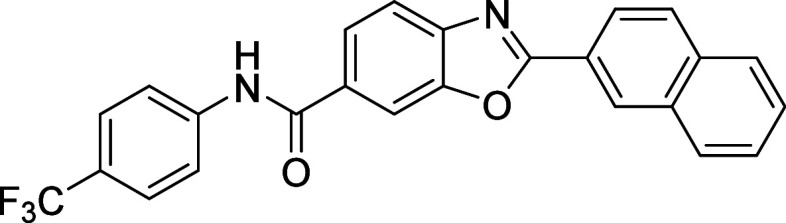



Light brown powder, Mp 285–286 °C, Yield 81%. FT-IR υ_max_ (cm^–1^) 3386.04 (N–H), 3072.55 (aromatic C–H), 2923.88 (aliphatic C–H), 1660.96 (C = O), 1599.29, 1550.66, 1519.74 (aromatic C = C), 828.87 (C–O), 1068.17 (C–N). ^1^H NMR (400 MHz, *d*_*6*_-DMSO) δ 10.71 (bs, 1H, -NH), 8.88 (s, 1H, Ar–H), 8.42 (s, 1H, Ar–H), 8.28 (dd, J= 0.6 Hz, J= 8.6 Hz, 1H, Ar–H), 8.22–8.12 (m, 2H, Ar–H), 8.09–8.00 (m, 4H, Ar–H), 7.97 (d, J= 8.2 Hz, 1H, Ar–H), 7.74 (d, J= 8.6 Hz, 1H, Ar–H), 7.70–7.63 (m, 2H, Ar–H). ^13^C NMR (100 MHz, *d*_*6*_-DMSO) δ 165.7, 165.1, 150.5, 144.9, 143.2, 136.8, 135.0, 132.9, 132.1, 129.6, 129.5, 128.9, 128.8, 128.3, 127.8, 126.5, 126.4 (quasi q, J= 3.4 Hz), 125.5, 124.1, 120.6, 120.5, 120.0, 111.1. HRMS [M + H]; Calculated for C_25_H_16_F_3_N_2_O_2_: 433.1158, Found: 433.1158.

#### 4.1.18 *N*-(4-Methoxyphenyl)-2-(naphthalen-2-yl)­benzo­[*d*]­oxazole-6-carboxamide (15)

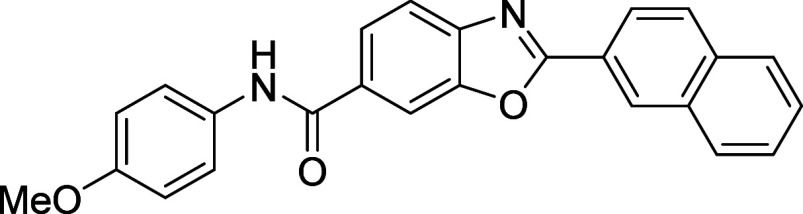



Light pink powder, Mp 238–239 °C, Yield 89%. FT-IR υ_max_ (cm^–1^) 3370.63 (N–H), 3000.02 (aromatic C–H), 2966.99 (aliphatic C–H), 1645.66 (C = O), 1554.97, 1516.38, 1462.66 (aromatic C = C), 819.87 (C–O), 1058.87 (C–N). ^1^H NMR (400 MHz, *d*_*6*_-DMSO) δ 10.27 (bs, 1H, -NH), 8.87 (bs, 1H, Ar–H), 8.38 (d, J= 1.0 Hz, 1H, Ar–H), 8.28 (dd, J= 1.6 Hz, J= 8.7 Hz, 1H, Ar–H), 8.22–8.18 (m, 1H, Ar–H), 8.15 (d, J= 8.7 Hz, 1H, Ar–H), 8.07–8.01 (m, 2H, Ar–H), 7.94 (d, J= 8.3 Hz, 1H, Ar–H), 7.73–7.61 (m, 4H, Ar–H), 6.97–6.91 (m, AA’BB’ system, 2H, Ar–H), 3.74 (s, 3H, -OMe). ^13^C NMR (100 MHz, *d*_*6*_-DMSO) δ 164.8, 164.7, 156.0, 150.5, 144.5, 134.9, 132.9, 132.8, 132.6, 129.6, 129.5, 128.9, 128.7, 128.3, 127.7, 125.2, 124.1, 123.8, 122.5, 119.8, 114.2, 110.8, 55.6. HRMS [M + H]; Calculated for C_25_H_19_N_2_O_3_: 395.1390, Found: 395.1391.

#### 4.1.19 2-(Naphthalen-2-yl)-*N*-(pyridin-4-yl)­benzo­[*d*]­oxazole-6-carboxamide (16)

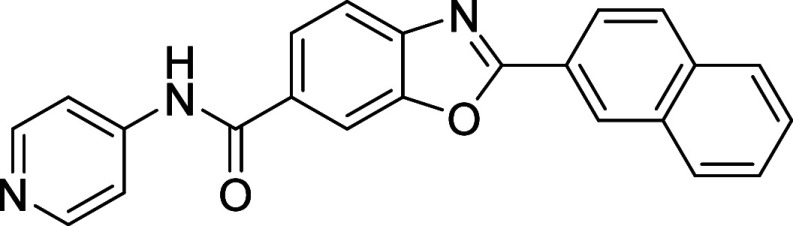



Light gray powder, Mp 253–254 °C, Yield 74%. FT-IR υ_max_ (cm^–1^) 3334.91 (N–H), 3056.07 (aromatic C–H), 2693.50 (aliphatic C–H), 1590.14 (C = O), 1546.35, 1509.05, 1474.43 (aromatic C = C), 819.08 (C–O), 1000.47 (C–N). ^1^H NMR (400 MHz, *d*_*6*_-DMSO) δ 10.73 (bs, 1H, -NH), 8.87 (bs, 1H, Ar–H), 8.49 (d, J= 5.0 Hz, 2H, Ar–H), 8.41 (s, 1H, Ar–H), 8.28–8.17 (m, 2H, Ar–H), 8.14 (d, J= 8.6 Hz, 1H, Ar–H), 8.08–8.00 (m, 2H, Ar–H), 7.97 (d, J= 8.2 Hz, 1H, Ar–H), 7.81 (d, J= 5.0 Hz, 2H, Ar–H), 7.70–7.61 (m, 2H, Ar–H). ^13^C NMR (100 MHz, *d*_*6*_-DMSO) δ 166.1, 165.2, 150.8, 150.4, 146.3, 145.1, 135.0, 132.9, 131.8, 129.6, 129.5, 128.9, 128.8, 128.3, 127.8, 125.5, 124.0, 123.7, 120.0, 114.5, 111.2. HRMS [M + H]; Calculated for C_23_H_16_N_3_O_2_: 366.1237, Found: 366.1237.

#### 4.1.20 2-([1,1′-Biphenyl]-4-yl)-*N*-phenylbenzo­[*d*]­oxazole-6-carboxamide (17)

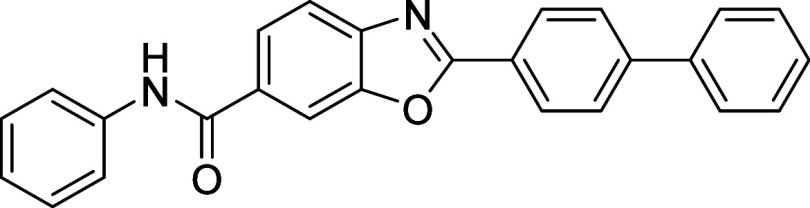



Brown powder, Mp 265–266 °C, Yield 84%. FT-IR υ_max_ (cm^–1^) 3363.43 (N–H), 3046.79 (aromatic C–H), 2906.61 (aliphatic C–H), 1677.27 (C = O), 1523.45, 1492.75 (aromatic C = C), 832.55 (C–O), 1058.32 (C–N). ^1^H NMR (400 MHz, *d*_*6*_-DMSO) δ 10.36 (bs, 1H, -NH), 8.40 (s, 1H, Ar–H), 8.31 (d, J= 7.4 Hz, 2H, Ar–H), 8.07–7.90 (m, 4H, Ar–H), 7.84–7.74 (m, 4H, Ar–H), 7.56–7.35 (m, 5H, Ar–H), 7.17–7.07 (m, 1H, Ar–H). ^13^C NMR (100 MHz, *d*_*6*_-DMSO) δ 165.2, 164.6, 150.4, 144.6, 144.2, 139.5, 139.2, 132.6, 129.3, 129.1, 128.9, 128.6, 128.0, 127.3, 125.4, 124.2, 120.8, 119.9, 110.9. HRMS [M + H]; Calculated for C_26_H_19_N_2_O_2_: 391.1441, Found: 391.1440.

#### 4.1.21 2-([1,1′-Biphenyl]-4-yl)-*N*-(4-methoxyphenyl)­benzo­[*d*]­oxazole-6-carboxamide (18)

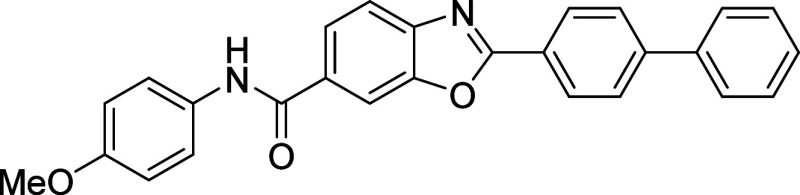



Light yellow powder, Mp 256–257 °C, Yield 87%. FT-IR υ_max_ (cm^–1^) 3363.70 (N–H), 3047,09 (aromatic C–H), 2904.96 (aliphatic C–H), 1670.41 (C = O), 1524.31, 1493,85 (aromatic C = C), 797.26 (C–O), 1004.77 (C–N). ^1^H NMR (400 MHz, *d*_*6*_-DMSO) δ 10.25 (bs, 1H, -NH), 8.38 (s, 1H, Ar–H), 8.33–8.27 (m, 2H, Ar–H), 8.05–8.00 (m, 1H, Ar–H), 7.98–7.90 (m, 3H, Ar–H), 7.81–7.75 (m, 2H, Ar–H), 7.74–7.67 (m, 2H, Ar–H), 7.55–7.48 (m, 2H, Ar–H), 7.45–7.41 (m, 1H, Ar–H), 6.97–6.90 (m, 2H, Ar–H), 3.74 (s, 3H, -OMe). ^13^C NMR (100 MHz, *d*_*6*_-DMSO) δ 166.9, 164.7, 164.5, 156.0, 150.4, 149.5, 147.4, 144.5, 139.2, 133.5, 132.6, 129.6, 128.6, 128.0, 127.3, 125.4, 122.4, 119.8, 114.2, 110.8, 55.6. HRMS [M + H]; Calculated for C_27_H_18_F_3_N_2_O_2_: 459.1314, Found: 459.1311.

#### 4.1.22 2-([1,1′-Biphenyl]-4-yl)-*N*-(4-(trifluoromethyl)­phenyl)­benzo­[*d*]­oxazole-6-carboxamide (19)

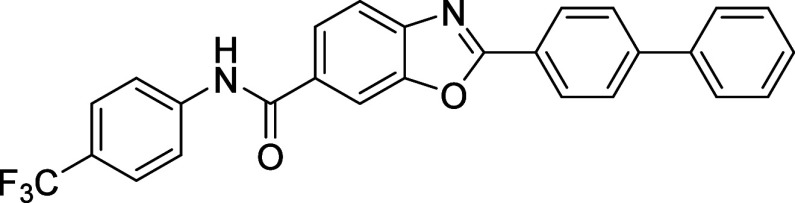



Light yellow powder, Mp 161–163 °C, Yield 74%. FT-IR υ_max_ (cm^–1^) 3362.52 (N–H), 3002.32 (aromatic C–H), 2937.54 (aliphatic C–H), 1641.20 (C = O), 1571.52, 1516.26, 1462.19 (aromatic C = C), 825.81 (C–O), 1036.81 (C–N). ^1^H NMR (400 MHz, *d*_*6*_-DMSO) δ 10.69 (bs, 1H, -NH), 8.42 (s, 1H, Ar–H), 8.33–8.25 (m, 2H, Ar–H), 8.08–7.90 (m, 5H, Ar–H), 7.83–7.67 (m, 4H, Ar–H), 7.56–7.36 (m, 4H, Ar–H). ^13^C NMR (100 MHz, *d*_*6*_-DMSO) δ 165.7, 164.8, 150.4, 144.9, 144.3, 143.2, 139.2, 132.0, 129.6, 128.9, 128.7, 128.6, 128.0, 127.3, 126.4 (quasi q, J= 3.2 Hz), 126.3, 125.5, 125.3, 120.6, 119.9, 111.1. HRMS [M + H]; Calculated for C_27_H_21_N_2_O_3_: 421.1546, Found: 421.1548.

#### 4.1.23 2-([1,1′-Biphenyl]-4-yl)-*N*-(pyridin-4-yl)­benzo­[*d*]­oxazole-6-carboxamide (20)

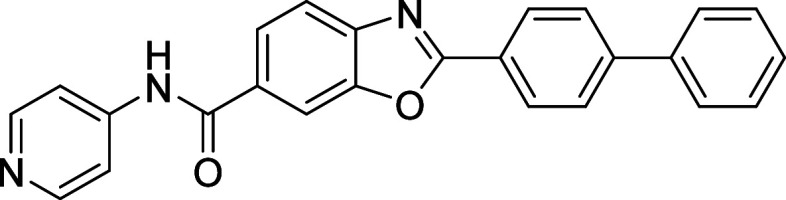



Light yellow powder, Mp 244–245 °C, Yield 75%. FT-IR υ_max_ (cm^–1^) 3269.62 (N–H), 3018.51 (aromatic C–H), 2946.42 (aliphatic C–H), 1644.99 (C = O), 1596.75, 1535.62, 1494.14 (aromatic C = C), 712.77 (C–O), 1022.70 (C–N). ^1^H NMR (400 MHz, *d*_*6*_-DMSO) δ 10.71 (bs, 1H, -NH), 8.49 (d, J= 3.5 Hz, 1H, Ar–H), 8.41 (s, 1H, Ar–H), 8.34–8.24 (m, 2H, Ar–H), 8.08–7.89 (m, 4H, Ar–H), 7.86–7.74 (m, 4H, Ar–H), 7.57–7.38 (m, 4H, Ar–H). ^13^C NMR (100 MHz, *d*_*6*_-DMSO) δ 166.1, 164.9, 150.8, 150.4, 146.3, 145.0, 144.3, 139.2, 131.7, 129.6, 128.9, 128.7, 128.0, 127.3, 125.6, 125.3, 120.0, 114.5, 111.2. HRMS [M + H]; Calculated for C_25_H_18_N_3_O_2_: 392.1393, Found: 392.1392.

### 4.2 Biological Activity Studies

#### 4.2.1 Cell Culture

The MCF-7 and MDA-MB-231 cell lines used in the study were cultured in DMEM medium containing l-glutamine, 10% fetal bovine serum (FBS), and 1% penicillin+streptomycin, in a 5% CO2 environment at 37 °C, in T-25 flasks, in an incubator.^33^

#### 4.2.2 MTT Analysis

MTT analysis is one of the most commonly used methods for assessing cell viability and proliferation. This assay is based on the metabolic activity of mitochondria. The enzyme succinate dehydrogenase, located in the mitochondria of living cells, reduces the MTT compound [3-(4,5-dimethylthiazol-2-yl)-2,5-diphenyltetrazolium bromide], a yellow, water-soluble tetrazolium salt. This reduction produces insoluble, purple-colored formazan crystals. The greater the metabolic activity within a cell population, the more formazan crystals are generated.

Cells were seeded at a density of 1 × 10^5^ cells per well in a 96-well plate and incubated at 37 °C for 24 h. The newly synthesized compounds were prepared at various concentrations (100, 50, 25, 12.5, 6.25, 3.125, and 1.56 μg/mL) and applied to the cells. After 24 h of incubation, 30 μL of MTT solution (5 mg/mL in PBS) was added to each well, followed by an additional 3 h of incubation. Absorbance was measured at 570 nm to calculate the percentage of viable cells.^39^

The percentage of live cells was calculated using the following formula:

%live cells=(Asample−Ablanc)/(Alive−Ablanc)x100

#### 4.2.3 mTOR ELISA Assay

Cells were seeded in 12-well plates and incubated for 24 h, after which the synthesized compounds were applied at their previously determined IC_5_0 concentrations. Following treatment, cells were harvested and lysed according to the manufacturer’s instructions. To evaluate mTOR inhibition, the Human mTOR (Mammalian Target of Rapamycin) ELISA Kit (E-EL-H1655) was used. Total protein concentrations in the cell lysates were measured, and all samples were normalized to equal total protein amounts prior to ELISA loading. The assay was performed according to the kit protocol, and mTOR concentrations were expressed as ng/mL, representing relative mTOR levels per unit of total cellular protein rather than absolute protein per cell number.

#### 4.2.4 LDH ELISA Assay

The ELISA kit (catalog number ELK-2236; ELK Biotech, China) was used in this study. The analysis was conducted following the manufacturer’s instructions. Eight different concentrations of the standard solution were prepared to generate the standard curve. A volume of 100 μL of antibody-coated antigen was added to each well, and the plate was incubated at 37 °C for 90 min. After incubation, the wells were washed three times consecutively with 300 μL of wash buffer. Subsequently, 100 μL of HRP-conjugated detection antibody was added to each well and incubated at 37 °C for 60 min. Following the second incubation, the wells were washed three times again. To initiate the enzymatic reaction, substrate solutions were mixed in equal volumes, and 100 μL of the mixture was added to each well. After incubation at 37 °C in the dark for 15 min, 50 μL of Stop Solution was added to each well to terminate the reaction. Absorbance values were measured at 450 nm. These absorbance values were then plotted on the standard curve to calculate the concentration of each sample.

#### 4.2.5 RT-qPCR Analysis

Three different groups were established in the experiment: MCF-7 cells treated with compounds COH-17 and COH-19 (at their respective IC_50_ doses) and a control group treated with DMSO. Total RNA was isolated from MCF-7 cells using the Hibrigen RNA isolation kit. The RNA samples obtained from each group were quantitatively measured by ultraviolet–visible (UV–vis) spectroscopy (Maestro NANO). The concentration (ng/μL) and purity (A260/A280 ratio) of each RNA sample were assessed. Complementary DNA (cDNA) synthesis was performed using the A.B.T. One-Step cDNA Synthesis Kit (Cat. No: C07–01–25) following the manufacturer’s protocol. In this study, the expression levels of BRCA1, BRCA2, PTEN, TP53, BCL-2, BAX, CASPASE-3, PI3K, AKT, and BRAD1 genes in MCF-7 cells were analyzed using the RT-qPCR method. RT-qPCR reactions were conducted in triplicate for each group. GAPDH (Gene ID: 2597) expression was used as an internal control for normalization. RT-qPCR analyses were performed using A.B.T. reagents (Cat. No: Q03–02–01) and optimized primer pairs for the target genes as follows:

BCL-2 [(Gene ID: 596), Forward: 5′-GGATAACGGAGGCTGGGATG-3′, Reverse: 5′-TGACTTCACTTGTGGCCCAG-3′],

BAX [(Gene ID: 581), Forward: 5′-GATGGACGGGTCCGGGG-3′, Reverse: 5′-CGATCCTGGATGAAACCCTGA-3′],

BRCA1 [(Gene ID:672), Forward: 5′-CTGAAGACTGCTCAGGGCTATC-3′, Reverse: 5′-AGGGTAGCTGTTAGAAGGCTGG-3′],

BRCA2 [(Gene ID:675), Forward: 5′-GGCTTCAAAAAGCACTCCAGATG-3′, Reverse: 5′-GGATTCTGTATCTCTTGACGTTCC-3′],

PTEN [(Gene ID:5728), Forward: 5′-TGAGTTCCCTCAGCCGTTACCT-3′, Reverse: 5′-GAGGTTTCCTCTGGTCCTGGTA-3′],

TP53­[(Gene ID:7157), Forward: 5′-CCTCAGCATCTTATCCGAGTGG-3′,Reverse: 5′-TGGATGGTGGTACAGTCAGAGC-3′]

Casp 3 [(Gene ID: 836), Forward: 5′-GGAAGCGAATCAATGGACTCTGG-3′, Reverse: 5′-GCATCGACATCTGTACCAGACC-3′],

PIK3CA [(Gene ID:5290 Forward: 5′-GAAGCACCTGAATAGGCAAGTCG-3′, Reverse: 5′-GAGCATCCATGAAATCTGGTCGC-3′],

AKT1 [(Gene ID:207 Forward: 5′-TGGACTACCTGCACTCGGAGAA-3′ Reverse: 5′-GTGCCGCAAAAGGTCTTCATGG-3′],

BARD1 [(Gene ID:580 Forward: 5′-TGCAGCCAAGAATGGGCATGTG-3′ Reverse: 5′-CTTCTCTGGTAGCAGCAATAGCG-3′]

Reactions were performed on the Roche LightCycler 96 system using the following cycling conditions: an initial denaturation at 95 °C for 5 min (1 cycle), followed by 40 cycles of denaturation at 95 °C for 15 s and annealing/extension at 60 °C for 1 min. Melting curve analysis was conducted in a single cycle at 95 °C for 10 s, 65 °C for 1 min, and 97 °C for 1 s to verify reaction specificity. Gene expression levels were quantified using the comparative threshold cycle (Ct) method, specifically the 2^–ΔΔCt^ approach.

#### 4.2.6 Determination of Bcl-2, Bax, Caspase-3, and p-53 Protein Levels Using the Western Blot Method

##### 4.2.6.1 Protein Isolation

The cell pellets were washed by adding 1 mL of A.B.T. PBS Buffer (pH 7.4, Phosphate Buffered Saline; Cat. No.: B02–01–01) and centrifuging at 2,500 rpm for 3 min. This washing step was repeated three times. After removing the PBS, 1 mL of ProtinEX Total Protein Extraction Solution (701–001, Geneall) and 10 μL of protease inhibitor cocktail were added to the pellet and mixed by pipetting. The mixture was incubated on ice for 10 min, then centrifuged at 16,000 rpm at 4 °C for 15 min. The supernatant was transferred to a clean tube. Protein quantification was performed using the Bradford Protein Assay Kit (P010, ABP Bioscience).

#### 4.2.7 Loading Samples onto Gel and SDS-PAGE Electrophoresis

The vertical gel system was placed inside the tank. The Bis-Tris Gradient Gel (NP0321, Invitrogen) was carefully removed from its packaging without disturbing the wells. To prepare the running buffer, 50 mL of 20× MOPS running buffer (NP0050, Invitrogen) was diluted with 950 mL of distilled water in a measuring cylinder to make 1× MOPS buffer. Then, 600 mL of the 1× MOPS running buffer was added to the tank. The wells were cleaned by pipetting. Five microliters of marker were loaded into the first and last wells of the gel cassette, and 15 μL of the prepared protein sample were loaded into the remaining wells. Electrophoresis was performed for 60 min at 150 V and 150 mA. The Invitrogen iBlot2 gel transfer system was used for blotting. After confirming that all marker bands had transferred to the membrane, the membrane was carefully placed in distilled water.The nitrocellulose membrane was placed in a blocking solution containing 5% bovine serum albumin (BSA) in PBS-T buffer with 0.1% Tween-20 and incubated overnight at 4 °C.

Protein expression levels were analyzed by Western blotting. Band intensities were quantified using ImageJ software and normalized to ACTB as the housekeeping protein. Relative expression levels were calculated as fold change compared to the control group.

#### 4.2.8 DAPI

MCF-7 cells were fixed with 4% glutaraldehyde at room temperature for 10 min. After fixation, the cells were washed three times with PBS, each wash lasting 5 min. For nuclear staining, a 300 nM DAPI solution was added to the cells and incubated in the dark for 5 min. Following staining, excess dye was removed, and the cells were washed three times with PBS. The cells were imaged using an Olympus fluorescence microscope with excitation/emission settings of 358/461 nm. The images were analyzed to assess nuclear morphology and cell density.

#### 4.2.9 Annexin V/PI (Apoptosis)

In our study, the Muse Annexin V & Dead Cell Kit (Cytek Biosciences, USA) was used to assess apoptosis and cell viability in MCF-7 cell cultures treated with compounds COH-17 and COH-19. Following the manufacturer’s protocol, cells collected from the culture medium were washed twice with phosphate-buffered saline (PBS), then mixed with 100 μL of Muse Annexin V & Dead Cell reagent (1:1) and incubated for 30 min. After incubation, the cell suspensions were analyzed using the Muse Cell Analyzer. The data obtained recorded the percentage of cells in each category: live, early apoptotic, and late apoptotic/necrotic cells.

#### 4.2.10 Cell Cycle

The Muse Cell Cycle Kit (Cytek Biosciences, USA) was used to quantitatively determine the cell cycle phases (G0/G1, S, and G2/M). Following the manufacturer’s protocol, cell samples obtained from the collected MCF-7 and COH-17 compounds were first treated with the Muse Cell Cycle reagent to permeabilize the cell membranes. Propidium iodide (PI) dye bound proportionally to the DNA content of the cells, producing a fluorescent signal that reflected the amount of DNA in each phase. The stained cell suspensions were analyzed using the Muse Cell Analyzer. This device measured the DNA content of each cell based on the fluorescence intensity from PI staining and classified the cell population into G0/G1, S, and G2/M phases. The resulting data were presented as histograms showing the percentage distribution of cells in each phase, enabling quantitative evaluation of effects on the cell cycle.

### 4.3 *In Silico* Studies Result

#### 4.3.1 Molecular Docking

The three-dimensional structure of the target protein (PDB ID: 4JT5) was obtained from the Protein Data Bank, and all crystallographic water molecules, ions, and cocrystallized ligands were removed prior to docking. Polar hydrogens were added, and Kollman charges were assigned using AutoDock vina, and the prepared protein was saved in PDBQT format. Twenty synthesized compounds were drawn in ChemDraw (version 2015), converted to three-dimensional structures in Avogadro, and energy-minimized using the MMFF94 force field. Gasteiger charges were assigned, rotatable bonds were defined, and ligands were exported in PDBQT format. Molecular docking simulations were performed using AutoDock Vina with a grid box covering the protein’s active site (dimensions 20*20*20 Å^3^, centered at coordinates X, Y, Z) and an exhaustiveness parameter of 8 to ensure adequate conformational sampling. For each ligand, the pose with the lowest predicted binding free energy (ΔG, kcal/mol) was selected for further analysis. Docking results were visualized using Discovery Studio Visualizer (version 2022), and interactions such as hydrogen bonds, hydrophobic contacts, and π–π stacking between ligands and key residues were identified. The docking protocol was validated by redocking the cocrystallized ligand, and a root-mean-square deviation (RMSD) below 2.0 Å confirmed the reliability of the procedure. This integrated approach allowed predictive evaluation of ligand binding affinities and interaction patterns, supporting *in silico* assessment of their potential bioactivity prior to experimental validation.^40^

#### 4.3.2 Molecular Dynamic Simulation

To investigate the dynamic behavior and structural stability of the complexes of COH-17 and 19 with mTOR (PDB ID: 4JT5), GROMACS molecular dynamics simulations were employed.^41,42^ The complexes were constructed using appropriate force field parameters, dissolved in an aqueous environment, and neutralized with counterions prior to equilibration. Following energy minimization and equilibration processes, production simulations were run for 100 ns under constant temperature and pressure conditions. The obtained trajectories were evaluated for stability using RMSD, for active site flexibility using RMSF, and for compactness using Gyration Radius analyses. Additionally, to quantitatively determine the binding strength, the nonbonded interaction energies, which are the sum of the Coulombic electrostatic and Lennard–Jones van der Waals components between the ligand and the mTOR protein, were calculated.

#### 4.3.3 ADME Prediction

COH-17 and 19 were subjected to comprehensive *in silico* analyses using the SwissADME web tool^43^ developed by the Swiss Institute of Bioinformatics (SIB) to evaluate their potential pharmacokinetic behavior and drug-similarity profiles. Canonical SMILES formats of the compounds were uploaded to the system and the following parameters were calculated collectively: GI absorption and Blood-Brain Barrier (BBB) penetration were visually analyzed using the BOILED Egg model and the overall profile was evaluated using the Bioavailability Radar. Finally, spider web modeling was applied to identify potentially risky structural motifs in the compounds. All quantitative results obtained were compiled to determine the suitability of the compounds as lead candidates.^42,43^

### 4.4 Statistical Analysis

For each gene, the p-value in the comparisons between the COH-17- or COH-19-treated groups and the control group was calculated based on the Student’s *t* test using the Data Analysis Center tool (https://www.qiagen.com/de/shop/genes-and-pathways/data-analysis-center-overview-page/). To assess changes in gene expression levels, fold change (FC) was calculated using the 2̂(-ΔΔCt) method. The formulas used for expression analysis were as follows: ΔCt = Cttarget – Ctreference (for normalization), ΔΔCt = ΔCtexperimental – ΔCtcontrol, and 2̂(-ΔΔCt).

The IC_5_0 values, mTOR levels, LDH levels, and relative 2̂(-ΔΔCt) values obtained from qPCR analysis in the COH-17- or COH-19-treated groups and control groups were analyzed using the independent samples *t* test or the Mann–Whitney U test, which assess differences between two independent groups, using SPSS version 22.0 (SPSS, Chicago, IL, USA). Statistical analyses involving more than two groups were performed using one-way analysis of variance (ANOVA) or the Kruskal–Wallis test. The normality of data distribution was evaluated using the Shapiro–Wilk test. The graphs were generated using SigmaPlot software. Statistical significance was defined at the 95% confidence level, with *p* < 0.05 considered statistically significant.

## Supplementary Material



## Notes

^1^H NMR and ^13^C NMR spectra for target compounds; FT-IR spectra for target compounds; LC-HRMS spectra for target compounds (PDF)

## ABBREVIATIONS

NMR: nuclear magnetic resonance
IC_5_0: half maximal inhibitory concentration
MCF-7: breast cancer cell line
MDA-MB-231: breast cancer cell line
MCF-10A: normal breast epithelial cell line
mTOR: mammalian target of rapamycin
LDH: lactate dehydrogenase
Bcl-2 B-: cell lymphoma 2
BRCA1: breast cancer type 1 susceptibility protein
BRCA2: breast cancer type 2 susceptibility protein
PTEN: phosphatase and tensin homologue
TP53: tumor protein p53
BAX: BCL2-associated X protein
Caspase-3: cysteine-aspartic acid protease 3
PI3K: phosphoinositide 3-kinase
AKT: protein kinase B (PKB)
BRAD1: breast cancer antiestrogen resistance 1
RT-qPCR: reverse transcription quantitative polymerase chain reaction
PIKK: phosphoinositide-3-kinase-related kinase family
HER: human epidermal growth factor receptor
VEGFR: vascular endothelial growth factor receptors
MTT: 3-(4,5-dimethylthiazol-2-yl)-2,5-diphenyltetrazolium bromide
SI: selectivity index
