# Supplementary material for: Synthesis and In Vitro/In silico Evaluation of Novel 2‑Aryl-6-carboxamide-Substituted Benzoxazole Derivatives with Anticancer Effects and mTOR Inhibitory Potential
Source: ACS Omega. 2026 May 22;11(22):32096–117. doi: 10.1021/acsomega.5c12045 (PMC13261455; doi:10.1021/acsomega.5c12045)
Supplement: Supplementary file 1 [file ao5c12045_si_001.pdf]

Synthesis and *In Vitro/İn silico* Evaluation of Novel 2-Aryl-6-Carboxamide-Substituted Benzoxazole Derivatives with Anticancer effects and mTOR Inhibitory Potential

Ceylan HEPOKUR[1*], Okan AYKAÇ[2],Sema MISIR[1], Şeyda AKIN[3], Burak KUZU[4], Naveen KOSAR[5], Recep KURNAZ[6], Öztekin ALGÜL[7]

***Ceylan HEPOKUR-** Sivas Cumhuriyet University, Faculty of Pharmacy, Department of Biochemistry, 58140, Sivas/Turkey

Okan AYKAÇ - Sivas Cumhuriyet University, Faculty of Pharmacy, Department of Pharmaceutical Chemistry, 58140, Sivas/Turkey

Sema MISIR-Sivas Cumhuriyet University, Faculty of Pharmacy, Department of Biochemistry, 58140, Sivas/Turkey

Şeyda AKIN-Sivas Cumhuriyet University, Faculty of Medicine, Department of Medical Biology, 58140,Sivas/Turkey

Burak KUZU-Pharmaceutical Chemistry Section, Van Yuzuncu Yil University, 65080 Van, Turkey

Naveen KOSAR- Chemistry Department,King Fahd University of Petroleum & Minerals, Dhahran, 31261, Saudi Arabia; Interdisciplinary Research Center for Refining and Advanced Chemicals, King Fahd University of Petroleum & Minerals, Dhahran 31261, Saudi Arabia

Recep Kurnaz- Department of Orthopaedics and Traumatology, Acıbadem State Hospital, Eskişehir/Turkey

Öztekin ALGÜL-Department of Pharmaceutical Chemistry, Faculty of Pharmacy, Mersin University, Mersin, Türkiye

Supplementary Material

Contents	Pages
**1.** Structures of the synthesized compounds **1-20**	**2-3**
**2.** ^{1}H- and ^{13}C-NMR spectrum copies of compounds **1-20**	**4-23**
**3.** HRMS spectrum copies of compounds **1-20**	**24-30**
**4.** FTIR spectrum	**31-50**

1. Structures of the synthesized compounds **1-20**

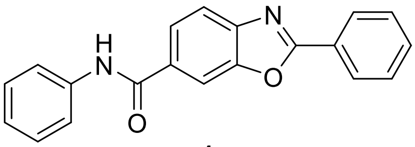

1

Chemical Formula: $C_{20}H_{14}N_2O_2$
Exact Mass: 314,1055

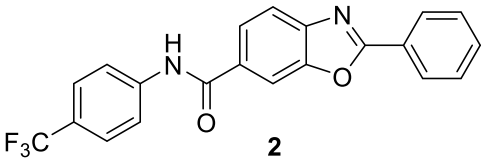

2

Chemical Formula: $C_{21}H_{13}F_3N_2O_2$
Exact Mass: 382,0929

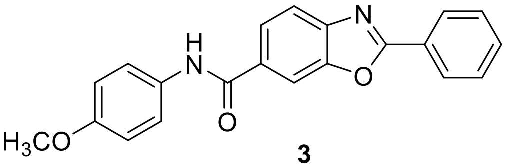

3

Chemical Formula: $C_{21}H_{16}N_2O_3$
Exact Mass: 344,1161

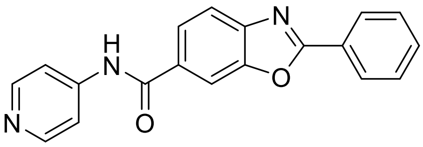

4

Chemical Formula: $C_{19}H_{13}N_3O_2$
Exact Mass: 315,1008

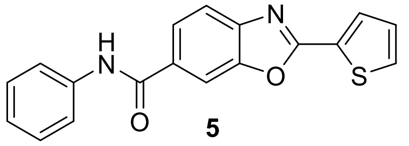

5

Chemical Formula: $C_{18}H_{12}N_2O_2S$
Exact Mass: 320,0619

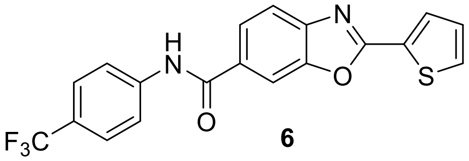

6

Chemical Formula: $C_{19}H_{11}F_3N_2O_2S$
Exact Mass: 388,0493

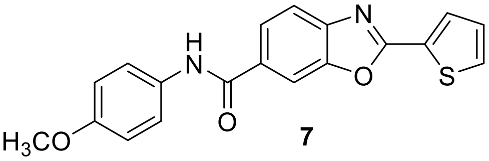

7

Chemical Formula: $C_{19}H_{14}N_2O_3S$
Exact Mass: 350,0725

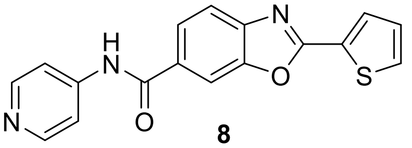

8

Chemical Formula: $C_{17}H_{11}N_3O_2S$
Exact Mass: 321,0572

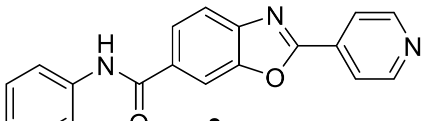

9

Chemical Formula: $C_{19}H_{13}N_3O_2$
Exact Mass: 315,1008

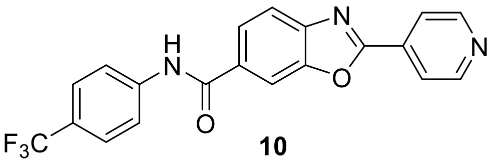

10

Chemical Formula: $C_{20}H_{12}F_3N_3O_2$
Exact Mass: 383,0882

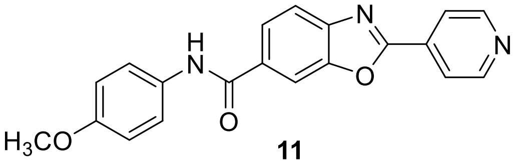

11

Chemical Formula: $C_{20}H_{15}N_{3}O_{3}$
Exact Mass: 345,1113

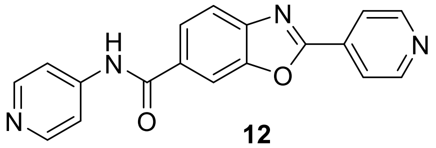

12

Chemical Formula: $C_{18}H_{12}N_{4}O_{2}$
Exact Mass: 316,0960

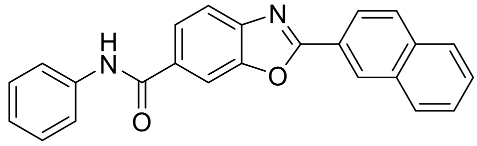

13

Chemical Formula: $C_{24}H_{16}N_{2}O_{2}$
Exact Mass: 364,1212

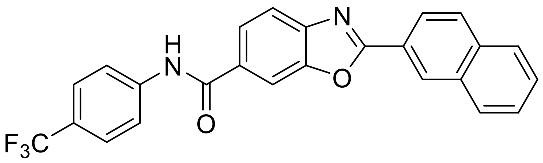

14

Chemical Formula: $C_{25}H_{15}F_{3}N_{2}O_{2}$
Exact Mass: 432,1086

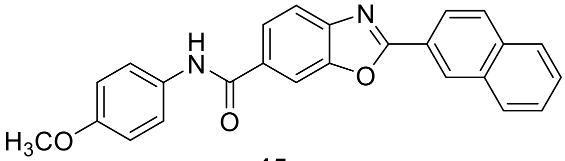

15

Chemical Formula: $C_{25}H_{18}N_{2}O_{3}$
Exact Mass: 394,1317

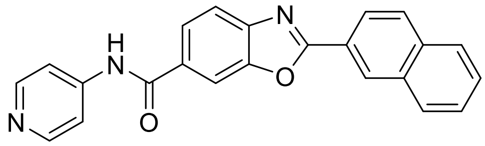

16

Chemical Formula: $C_{23}H_{15}N_{3}O_{2}$
Exact Mass: 365,1164

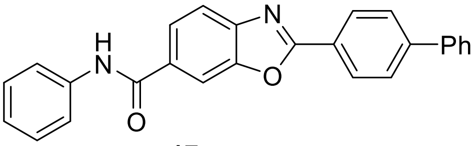

17

Chemical Formula: $C_{26}H_{18}N_{2}O_{2}$
Exact Mass: 390,1368

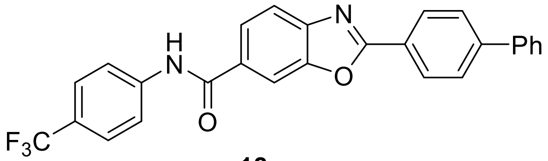

18

Chemical Formula: $C_{27}H_{17}F_{3}N_{2}O_{2}$
Exact Mass: 458,1242

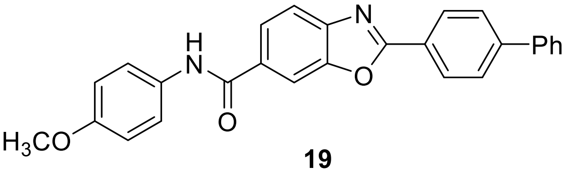

19

Chemical Formula: $C_{27}H_{20}N_{2}O_{3}$
Exact Mass: 420,1474

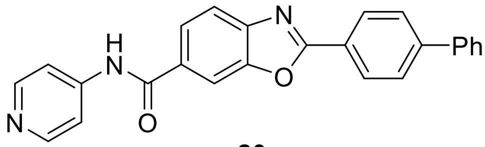

20

Chemical Formula: $C_{25}H_{17}N_{3}O_{2}$
Exact Mass: 391,1321

2. ^{1}H- and ^{13}C-NMR spectrum copies of compounds **1-20**

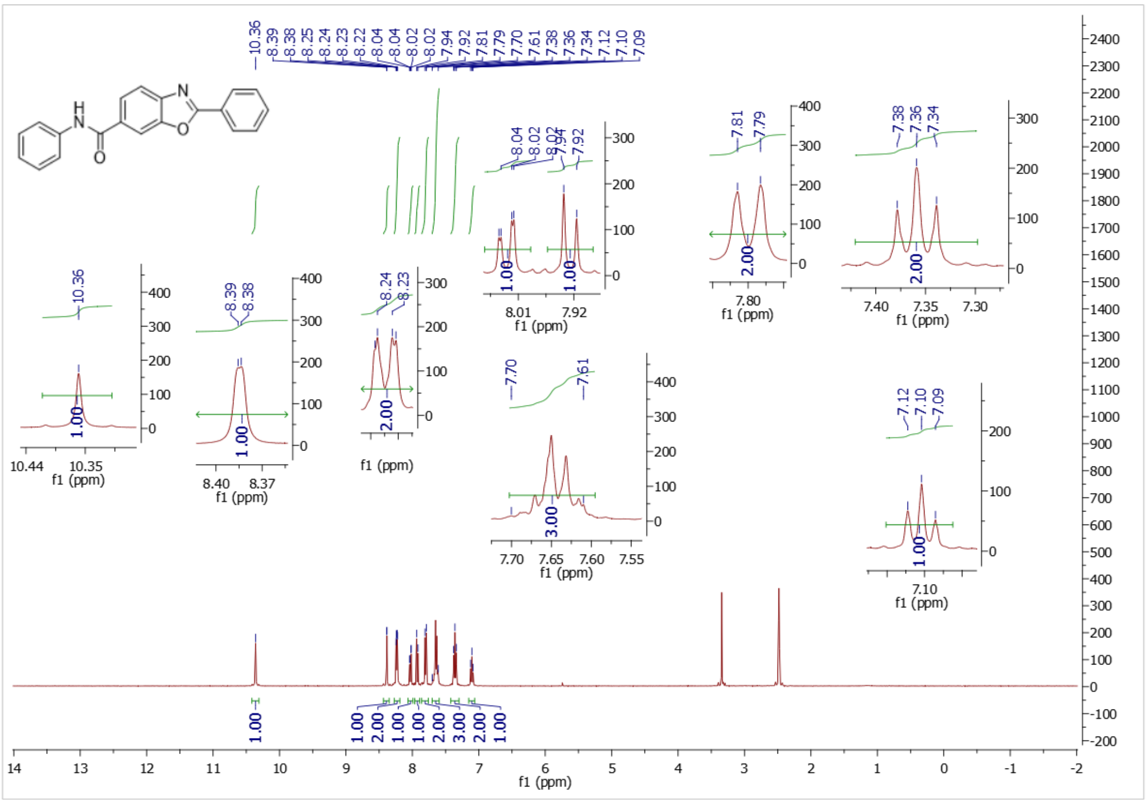

Figure S1. ^{1}H NMR spectrum of compound **1**

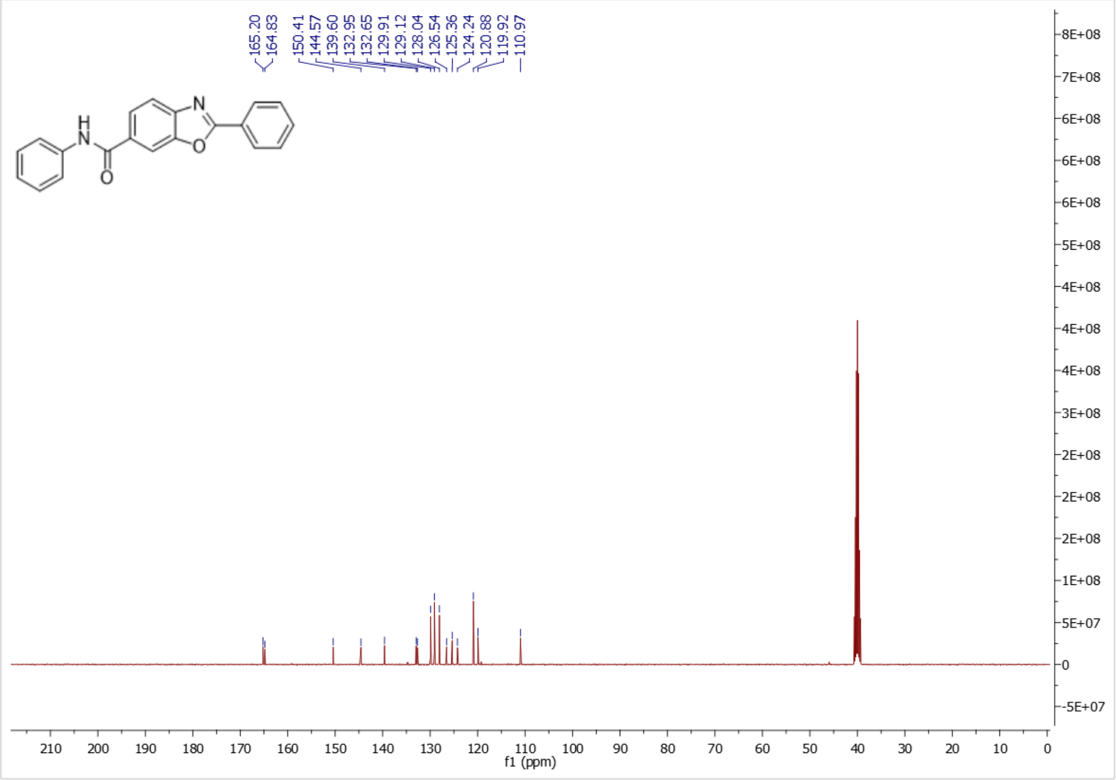

Figure S2. ^{13}C NMR spectrum of compound **1**

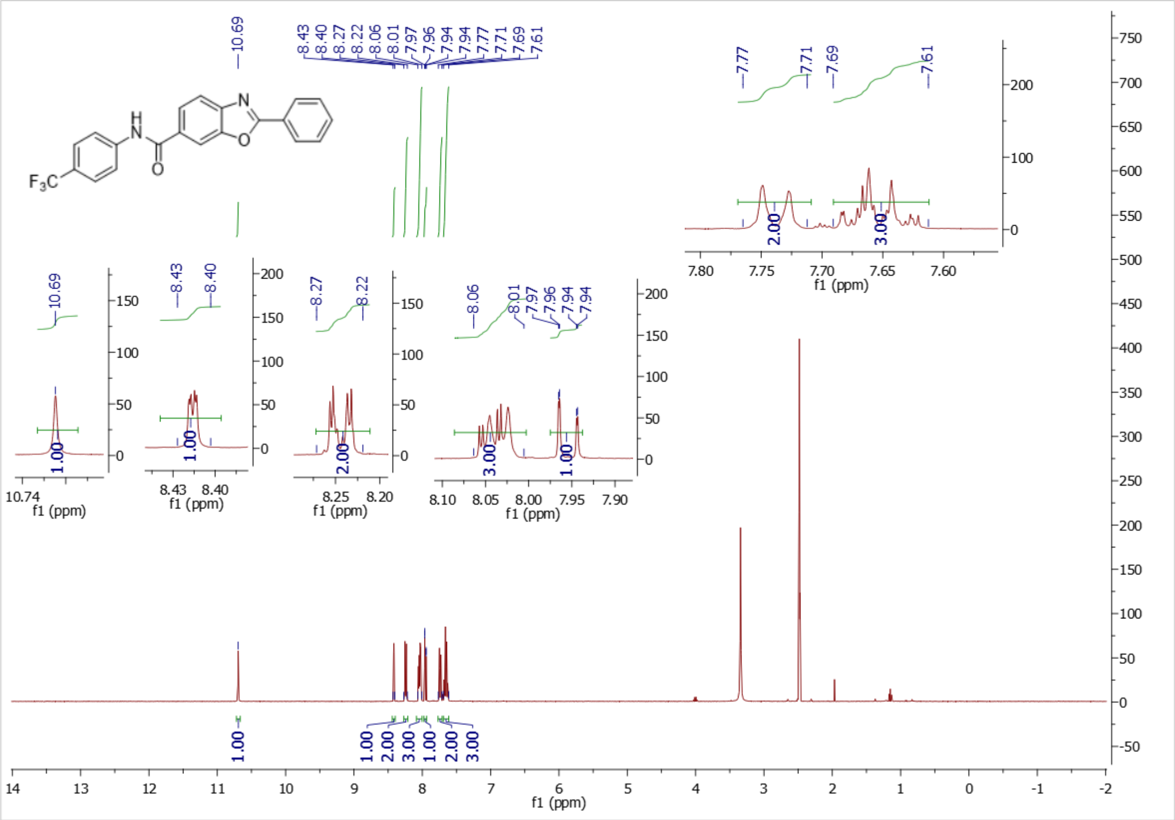

Figure S3. ^{1}H NMR spectrum of compound **2**

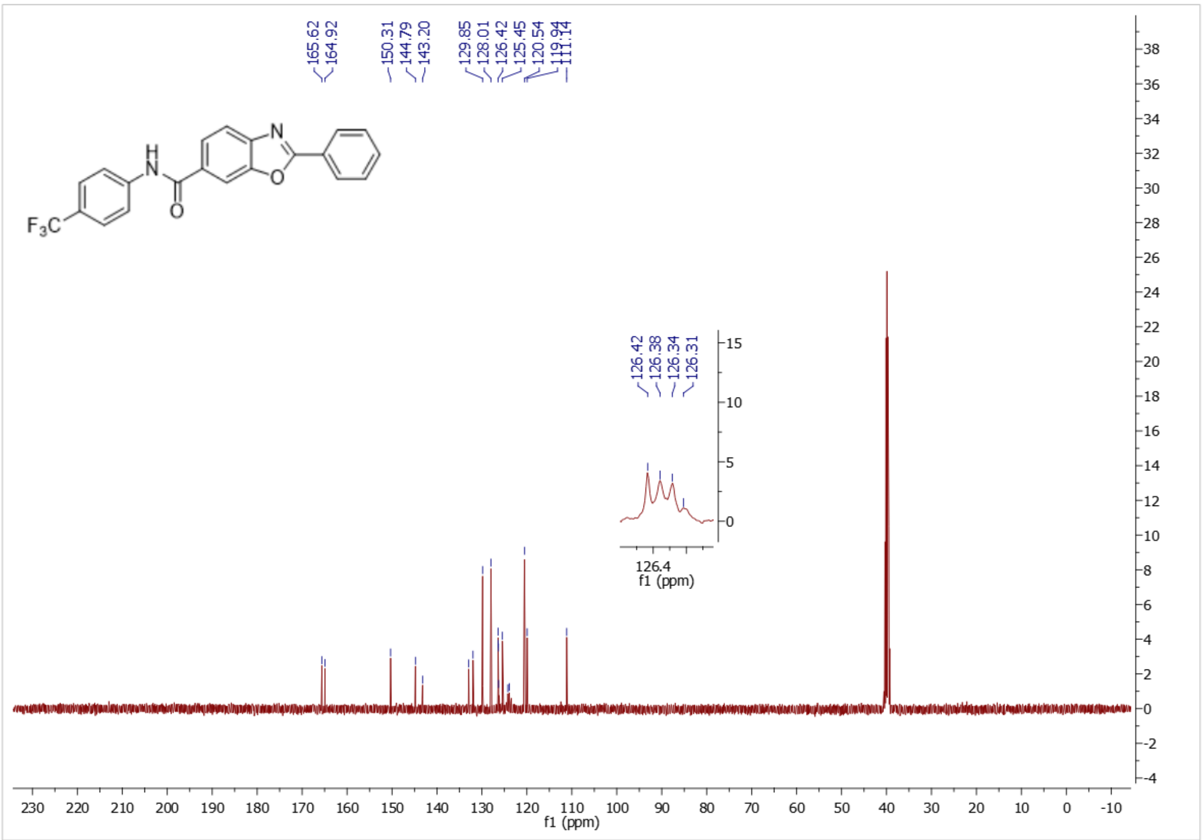

Figure S4. ^{13}C NMR spectrum of compound **2**

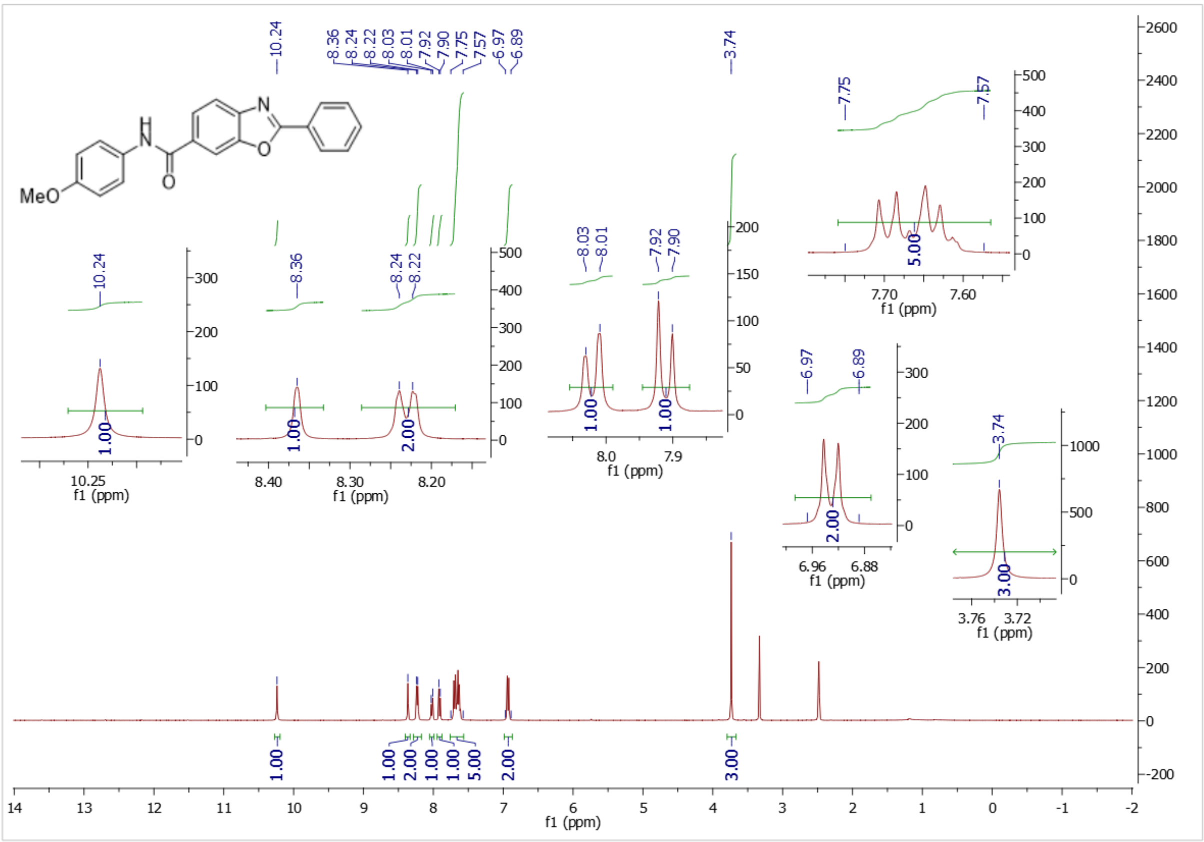

Figure S5. ^{1}H NMR spectrum of compound **3**

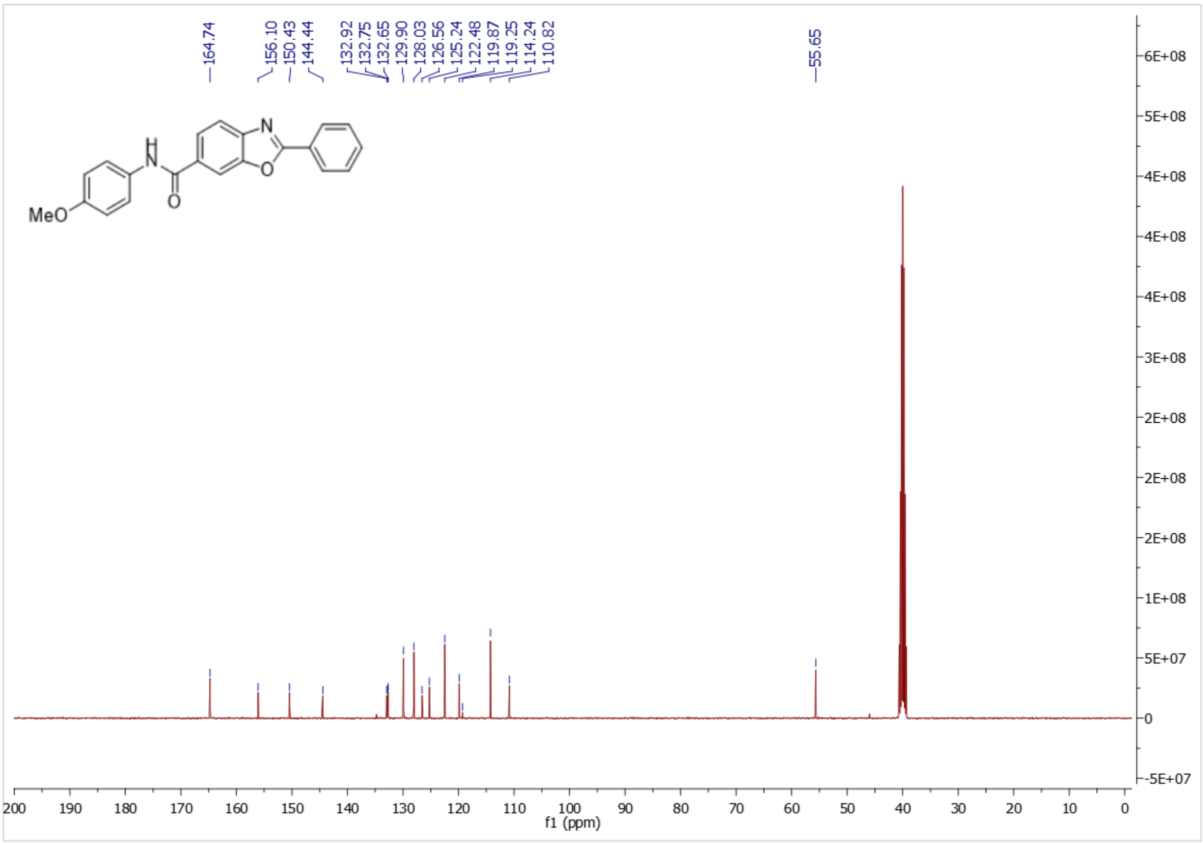

Figure S6. ^{13}C NMR spectrum of compound **3**

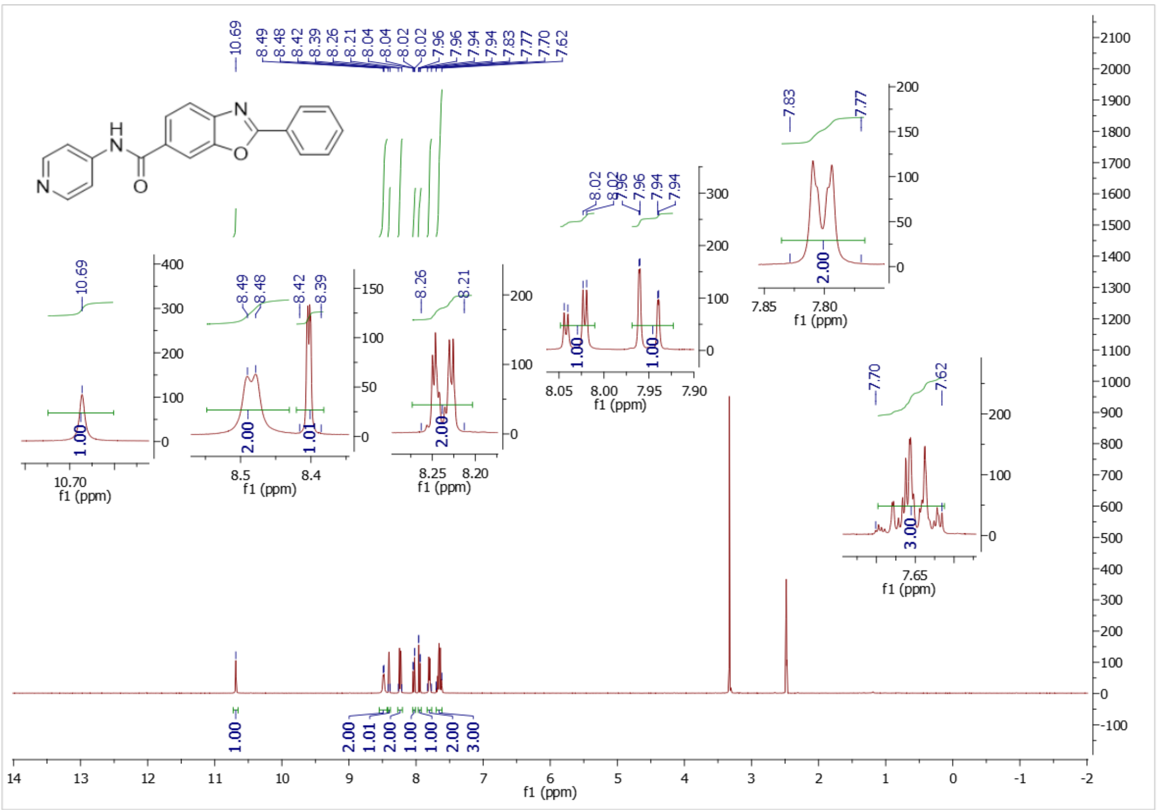

Figure S7. ^{1}H NMR spectrum of compound **4**

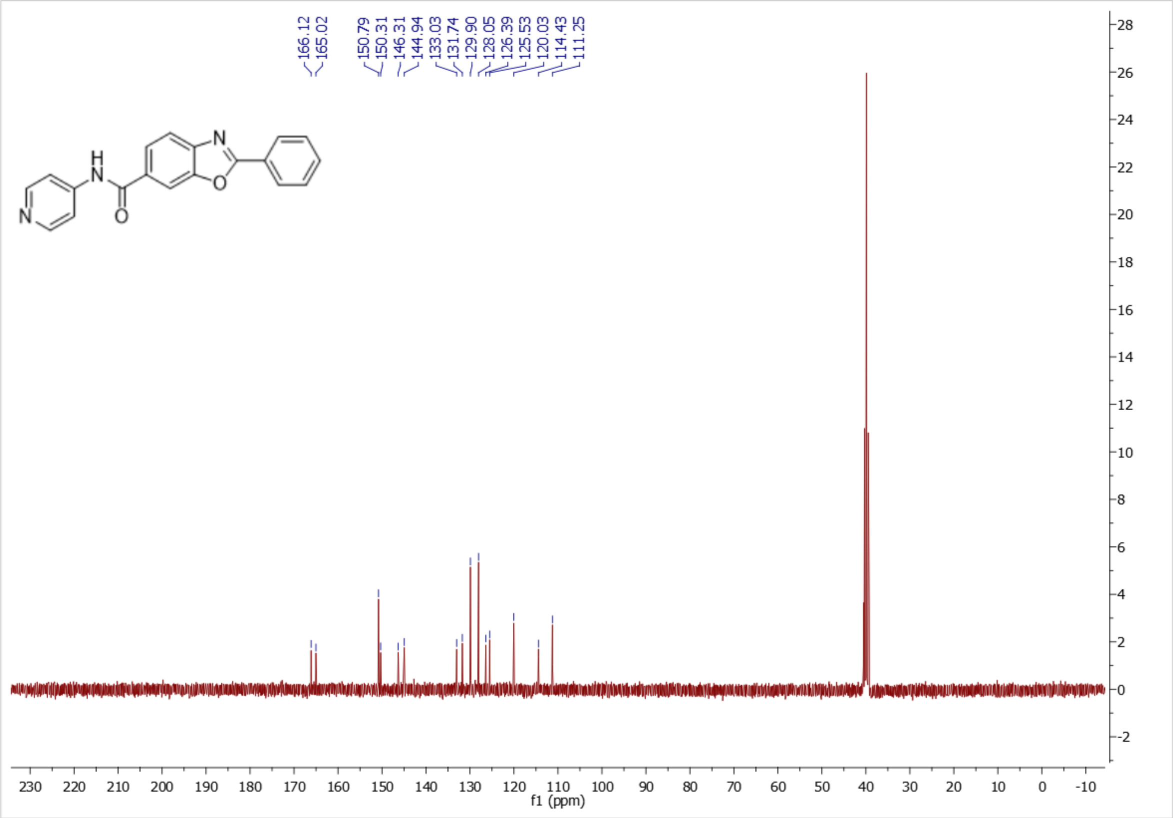

Figure S8. ^{13}C NMR spectrum of compound **4**

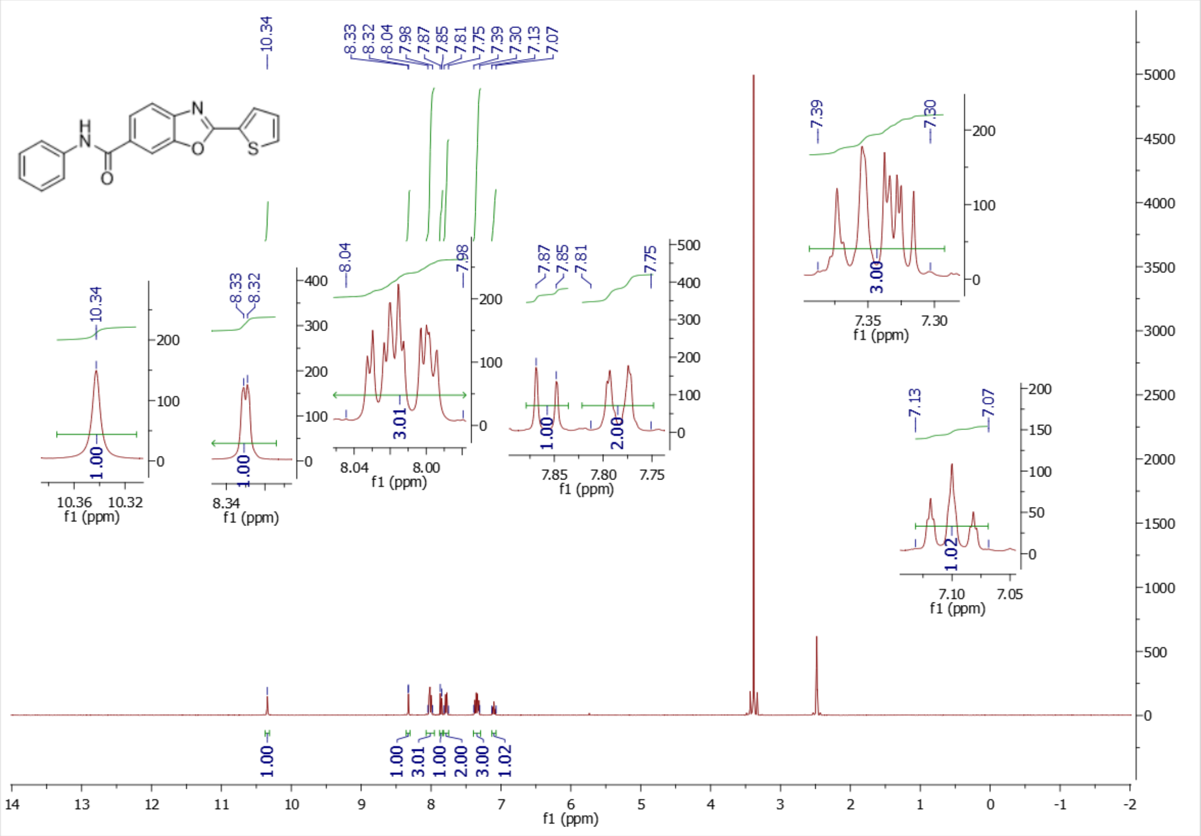

Figure S9. ^{1}H NMR spectrum of compound **5**

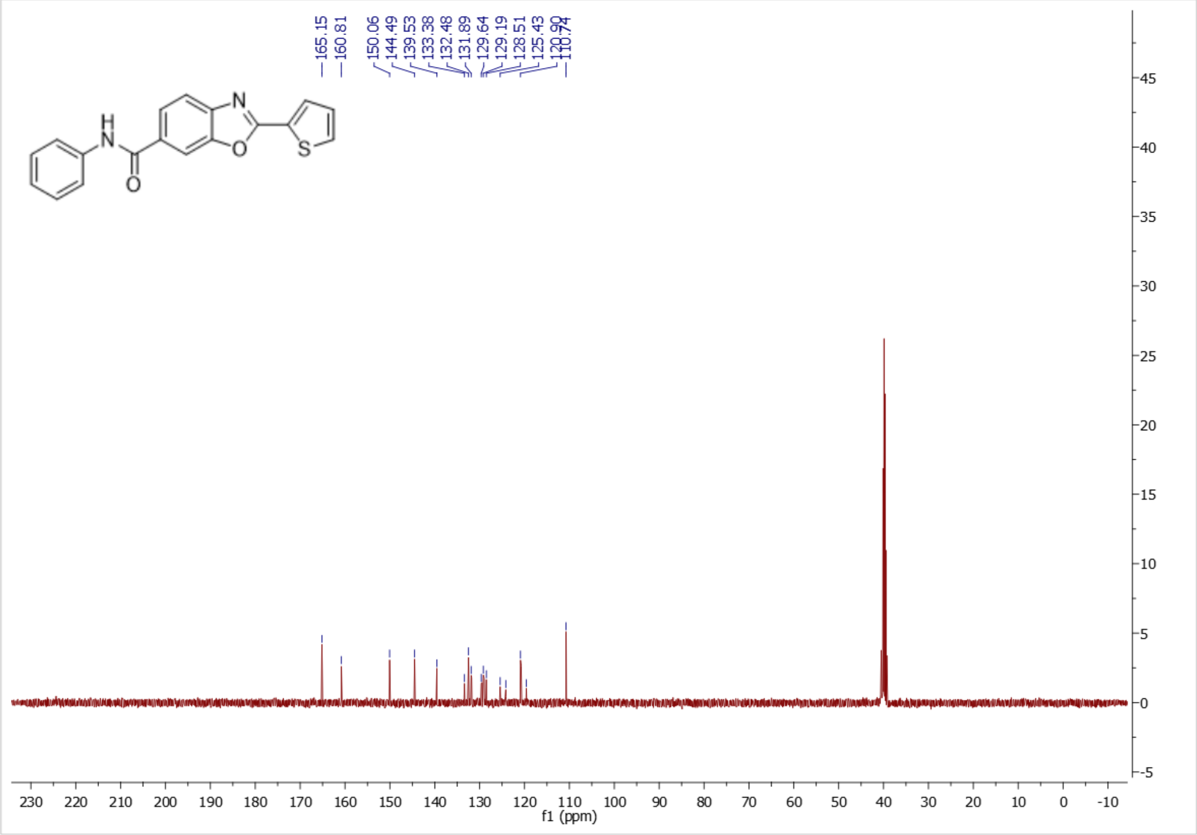

Figure S10. ^{13}C NMR spectrum of compound **5**

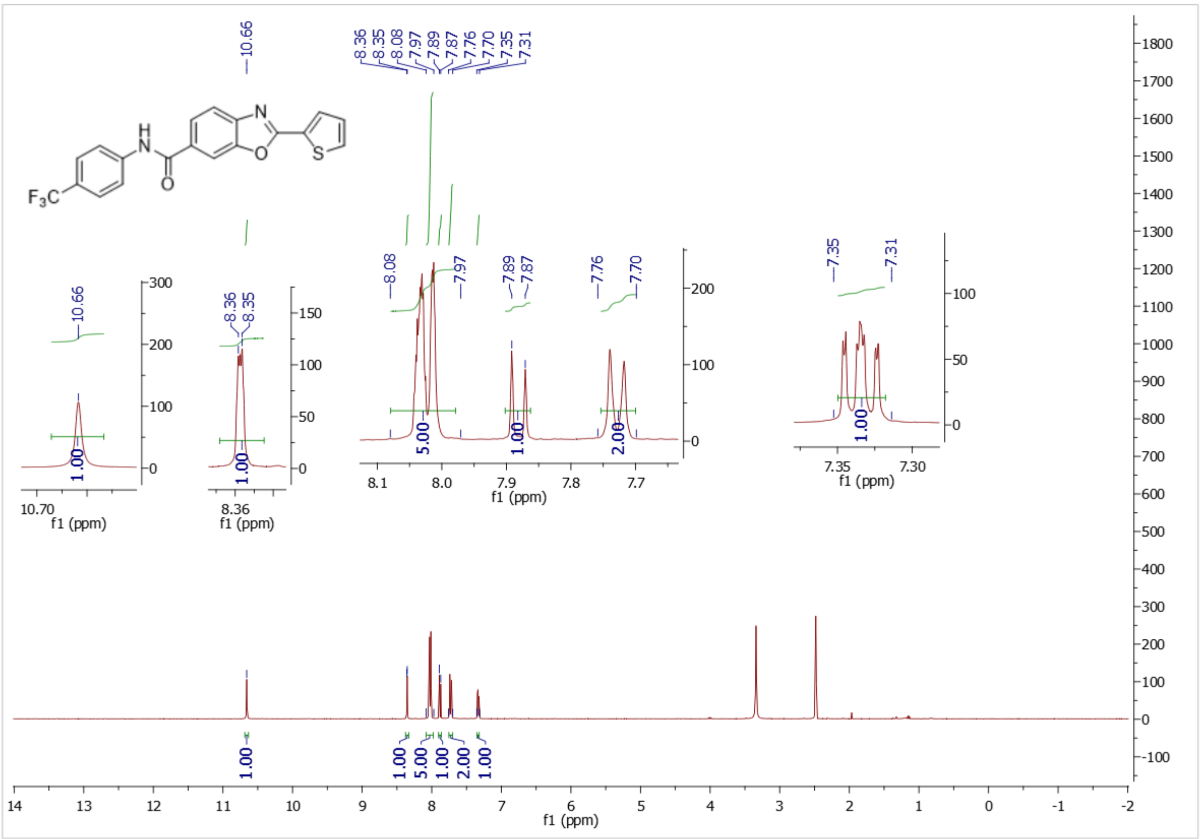

Figure S11. ^{1}H NMR spectrum of compound **6**

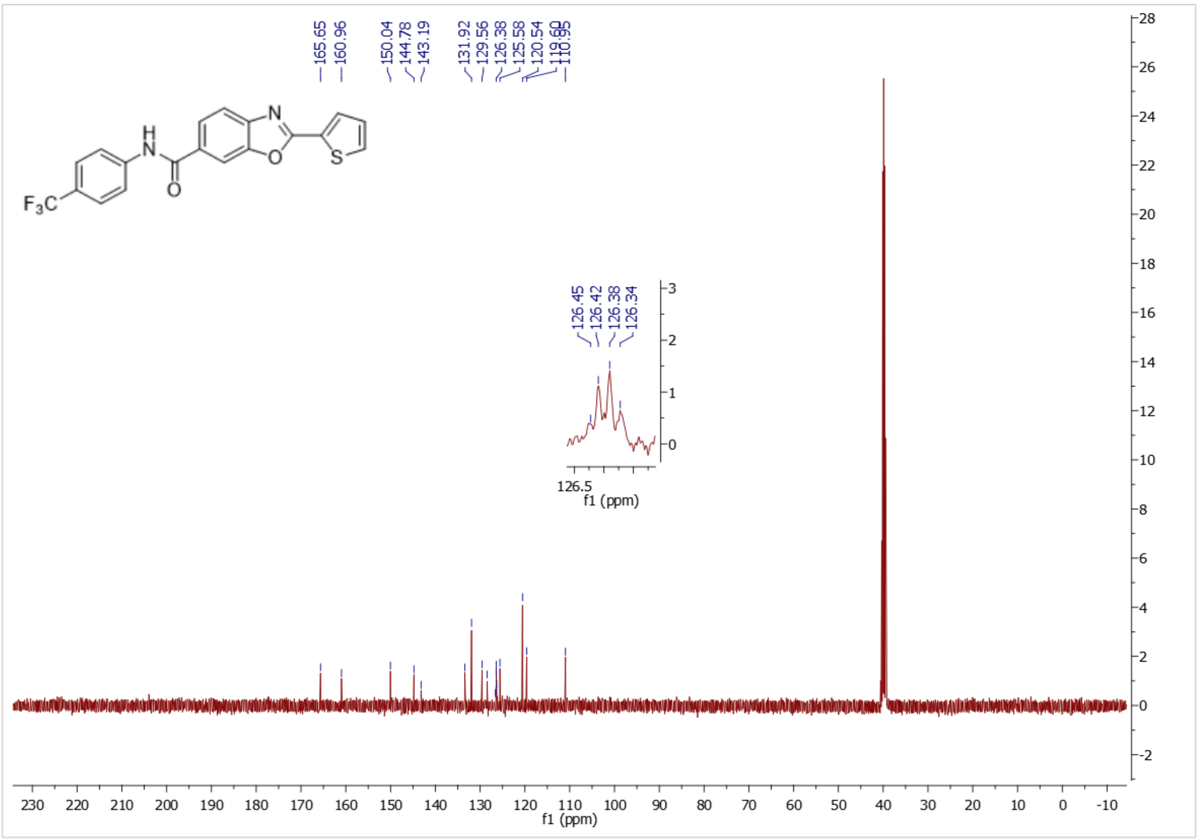

Figure S12. ^{13}C NMR spectrum of compound **6**

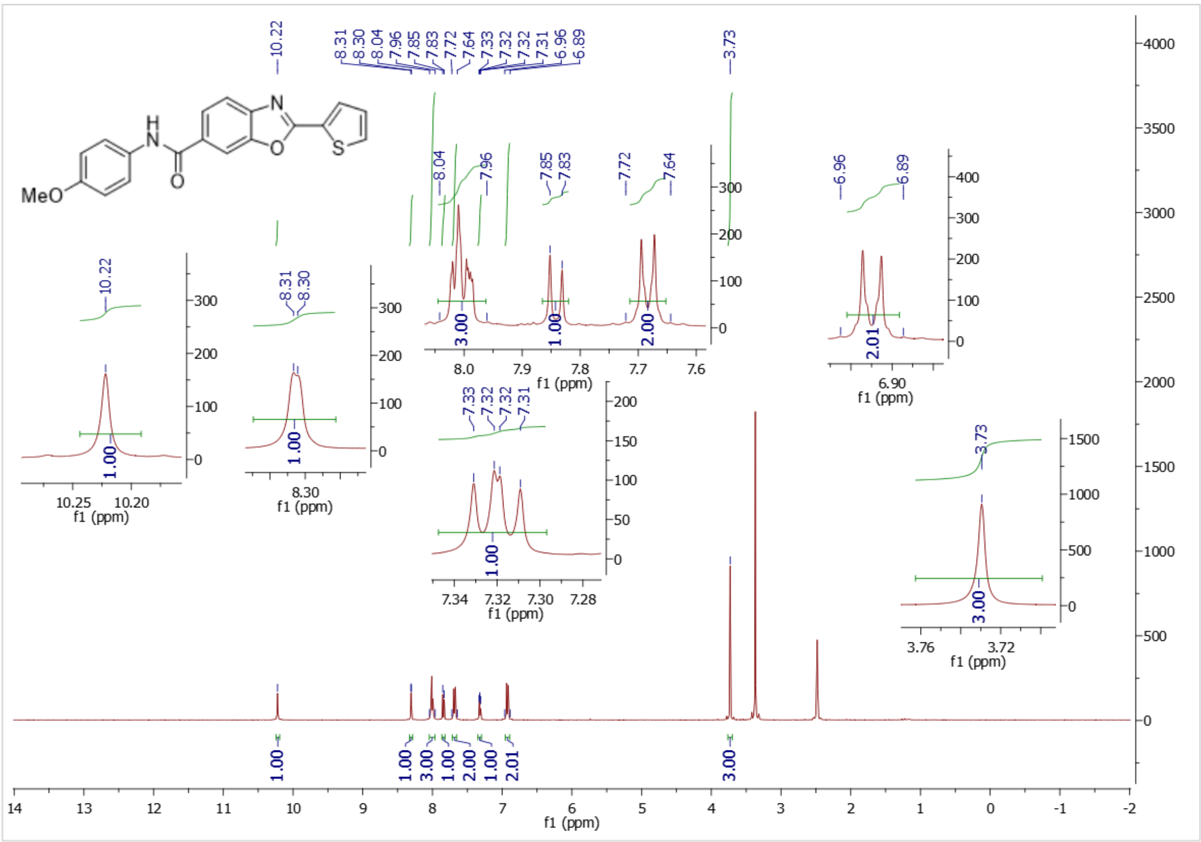

Figure S13. ^{1}H NMR spectrum of compound **7**

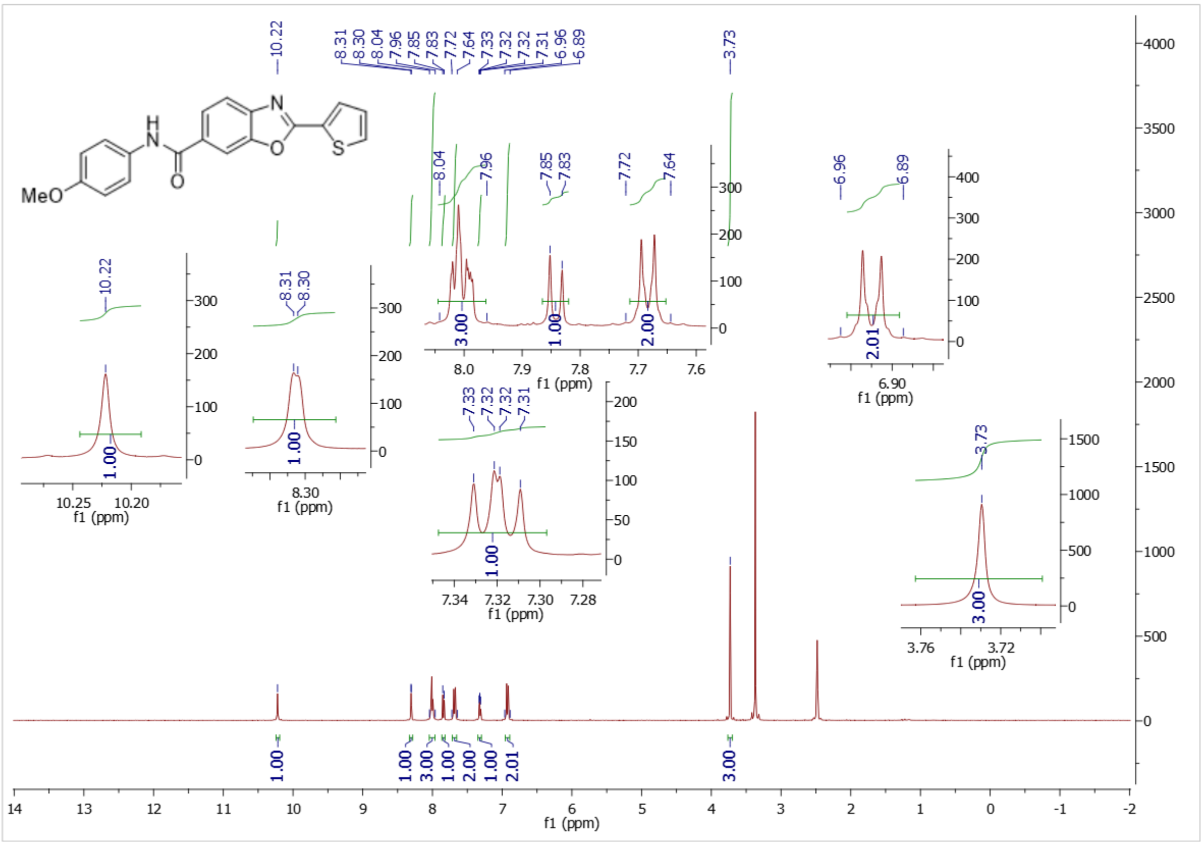

10.22
8.31 8.30 8.04 7.96 7.85 7.83 7.72 7.64 7.33 7.32 7.32 7.31 6.96 6.89
3.73
MeO
H
N
O
N
O
S
1.00 1.00 3.00 1.00 2.00 1.00 2.01 3.00
f1 (ppm)

Figure S14. ^{13}C NMR spectrum of compound **7**

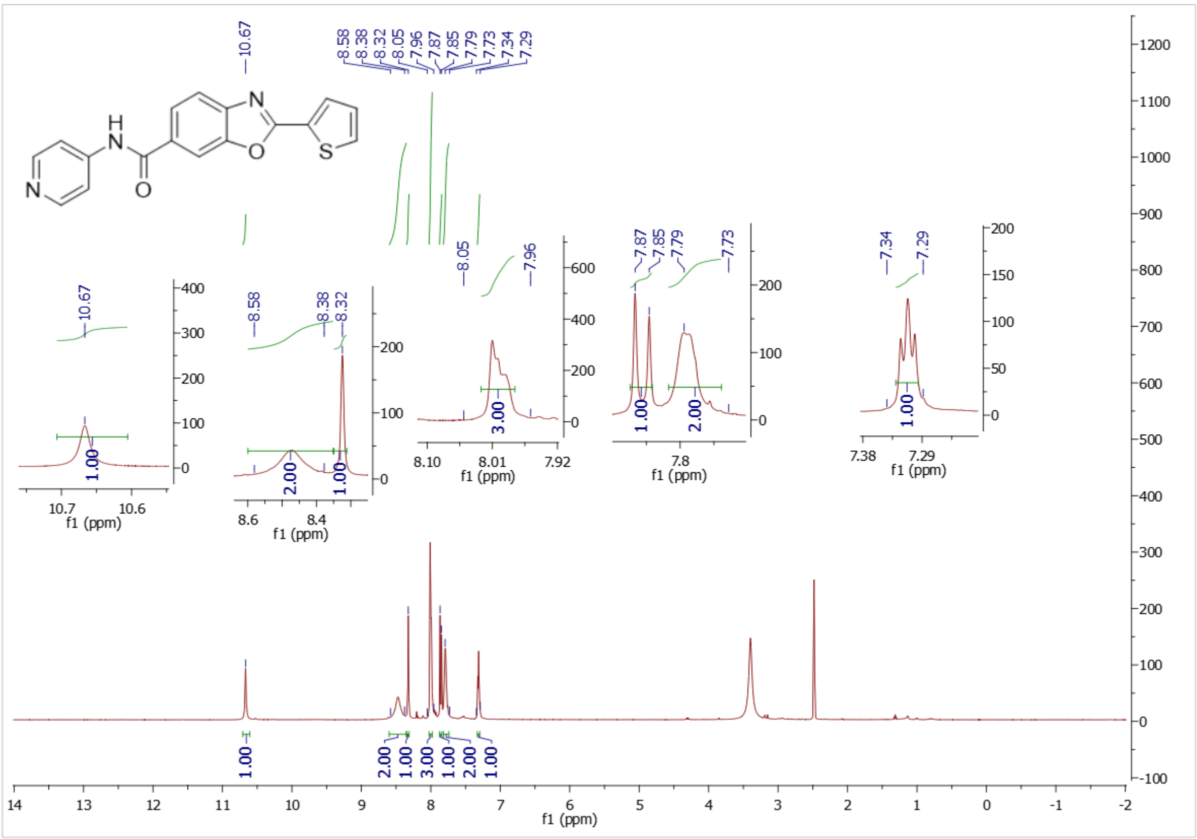

Figure S15. ^{1}H NMR spectrum of compound **8**

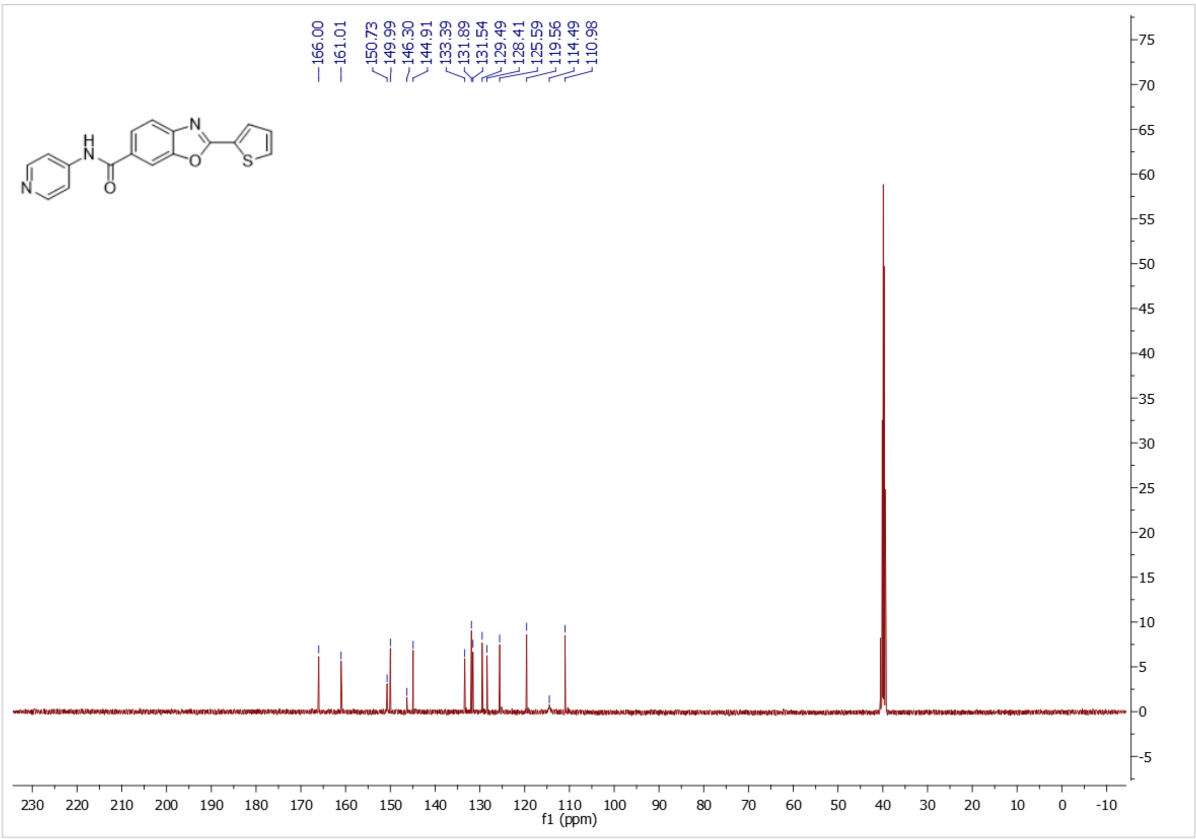

Figure S16. ^{13}C NMR spectrum of compound **8**

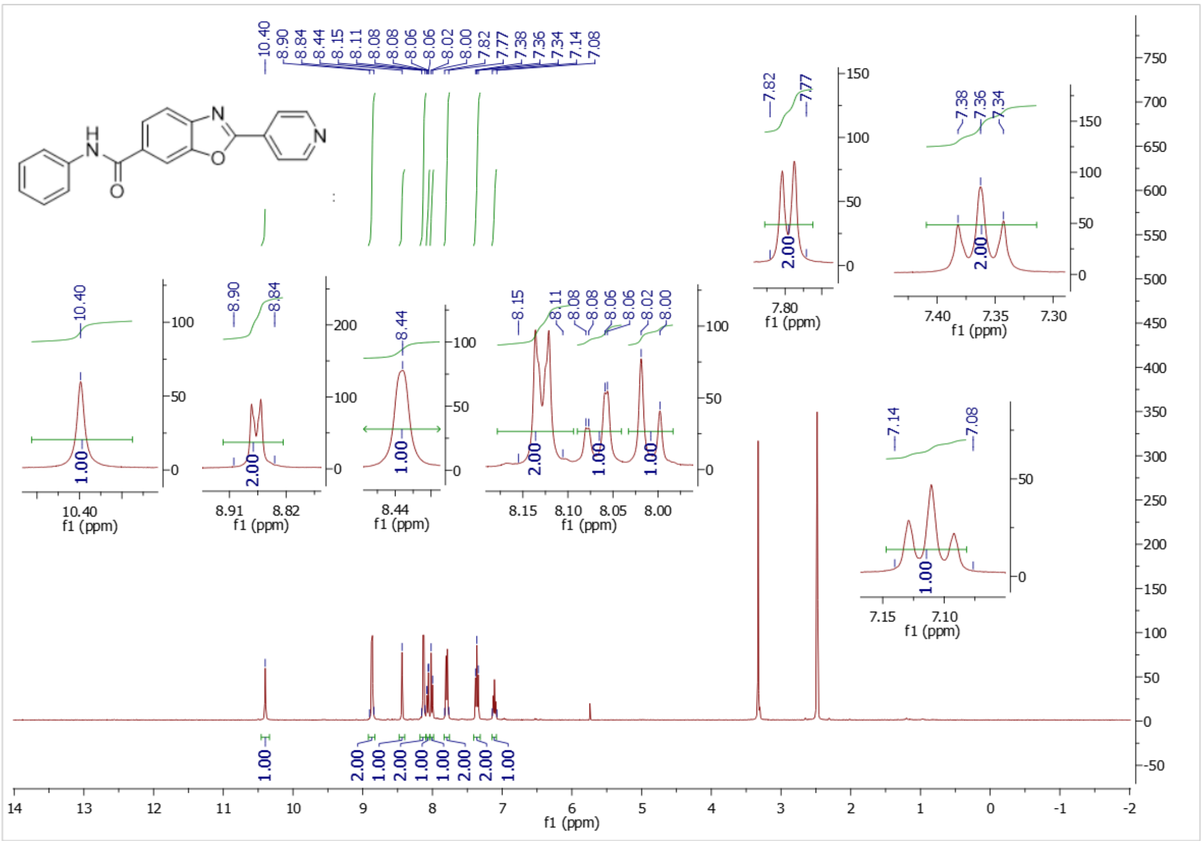

Figure S17. ^{1}H NMR spectrum of compound **9**

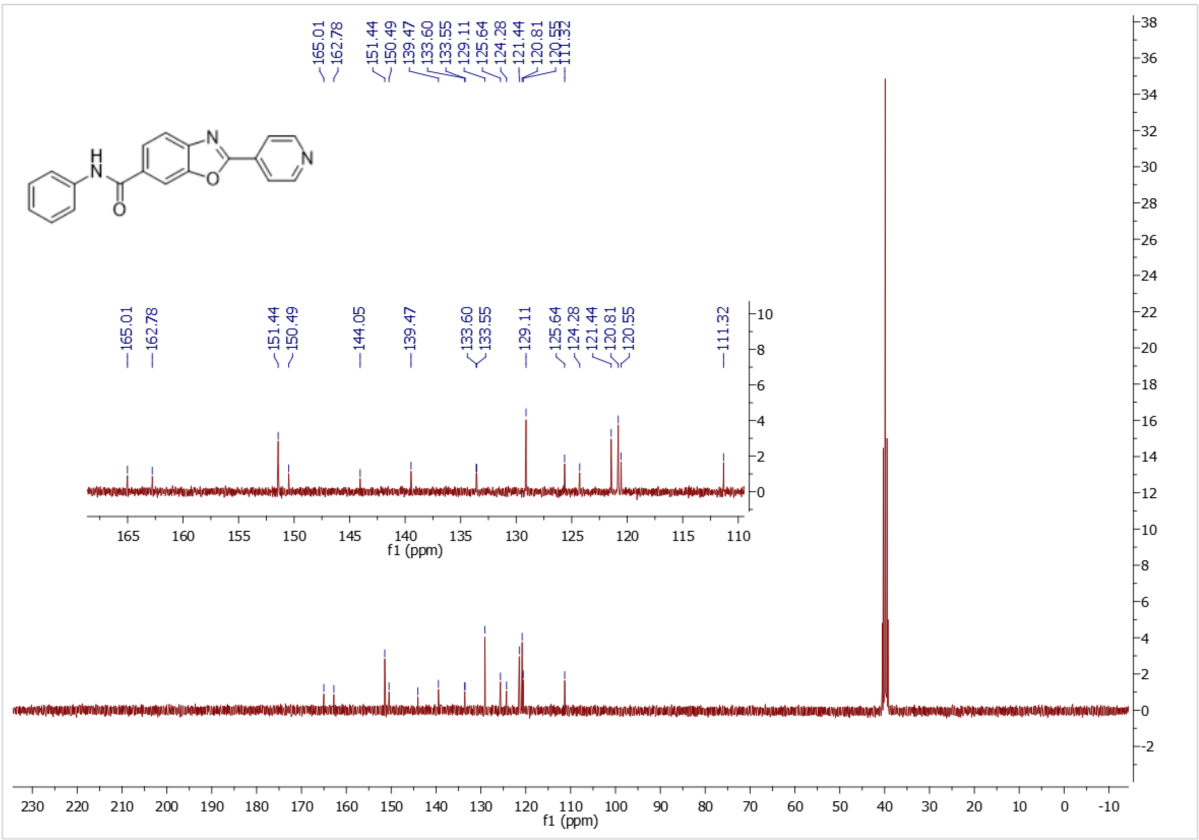

Figure S18. ^{13}C NMR spectrum of compound **9**

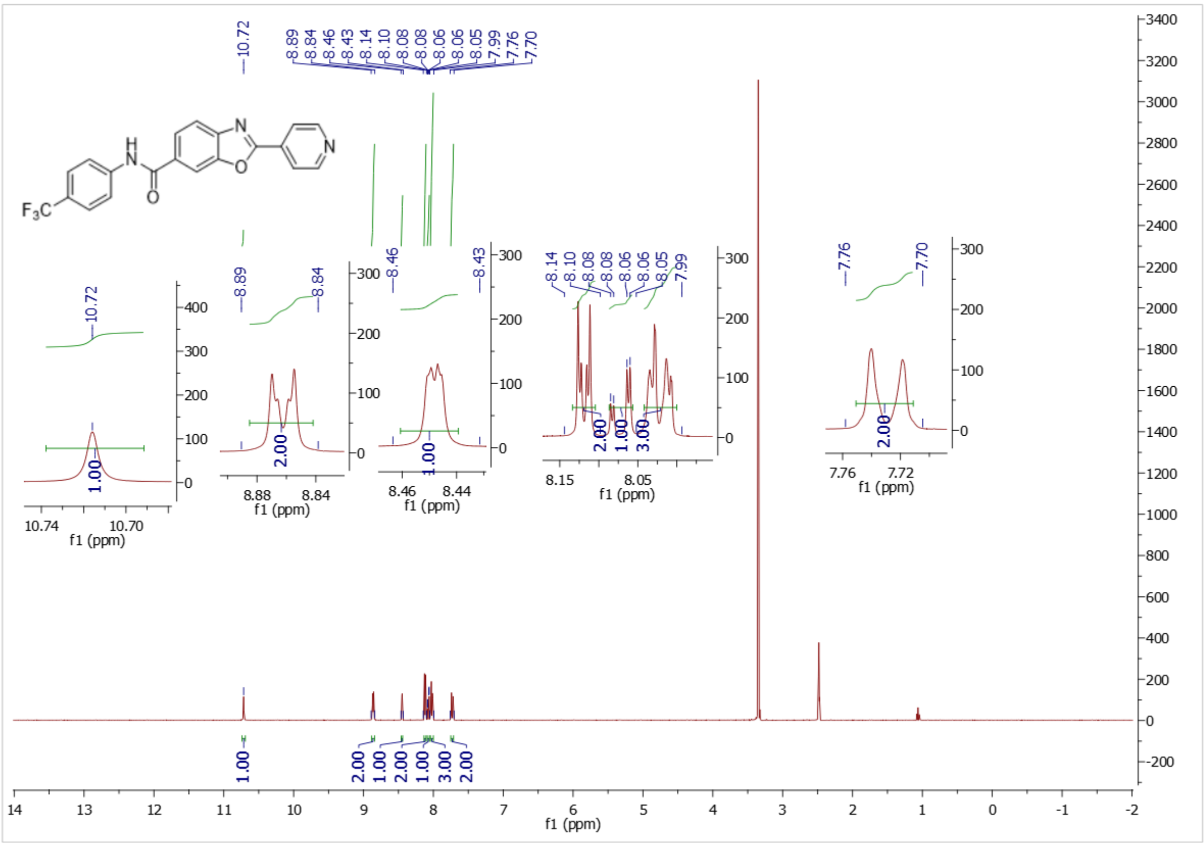

Figure S19. ^{1}H NMR spectrum of compound **10**

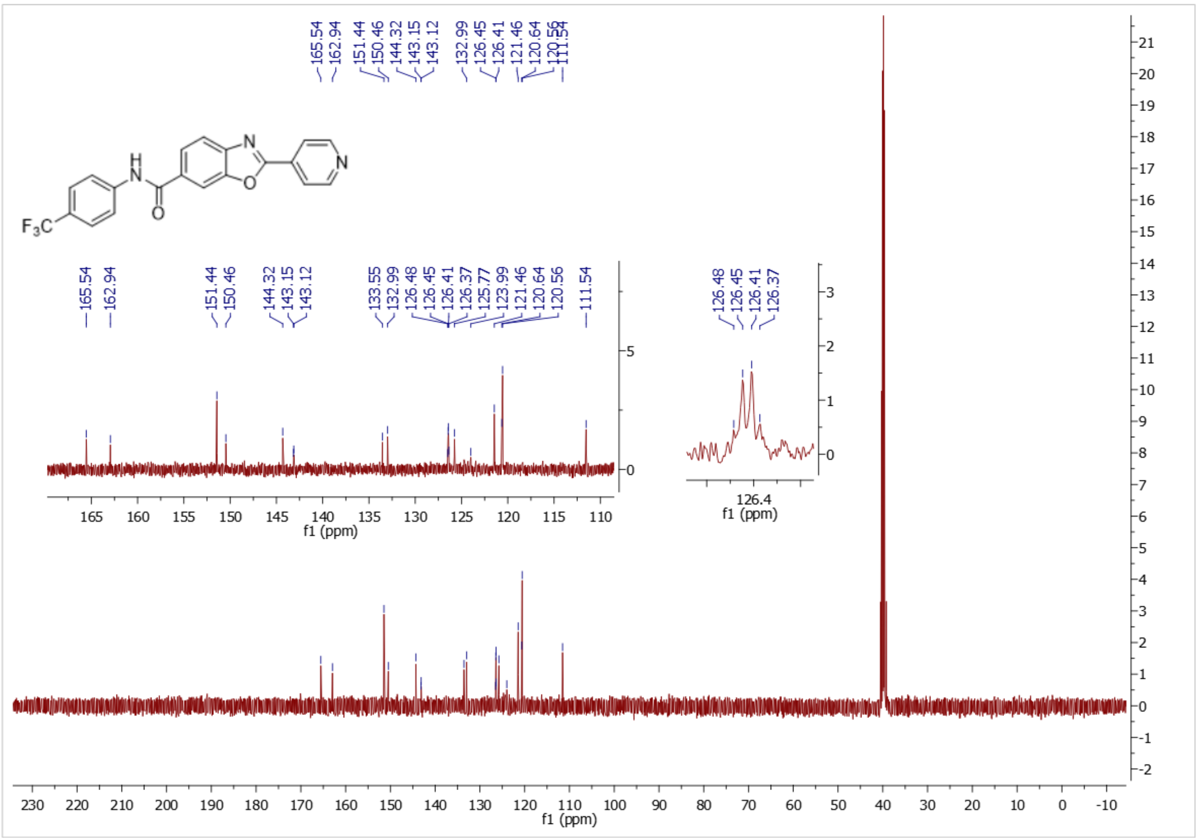

Figure S20. ^{13}C NMR spectrum of compound **10**

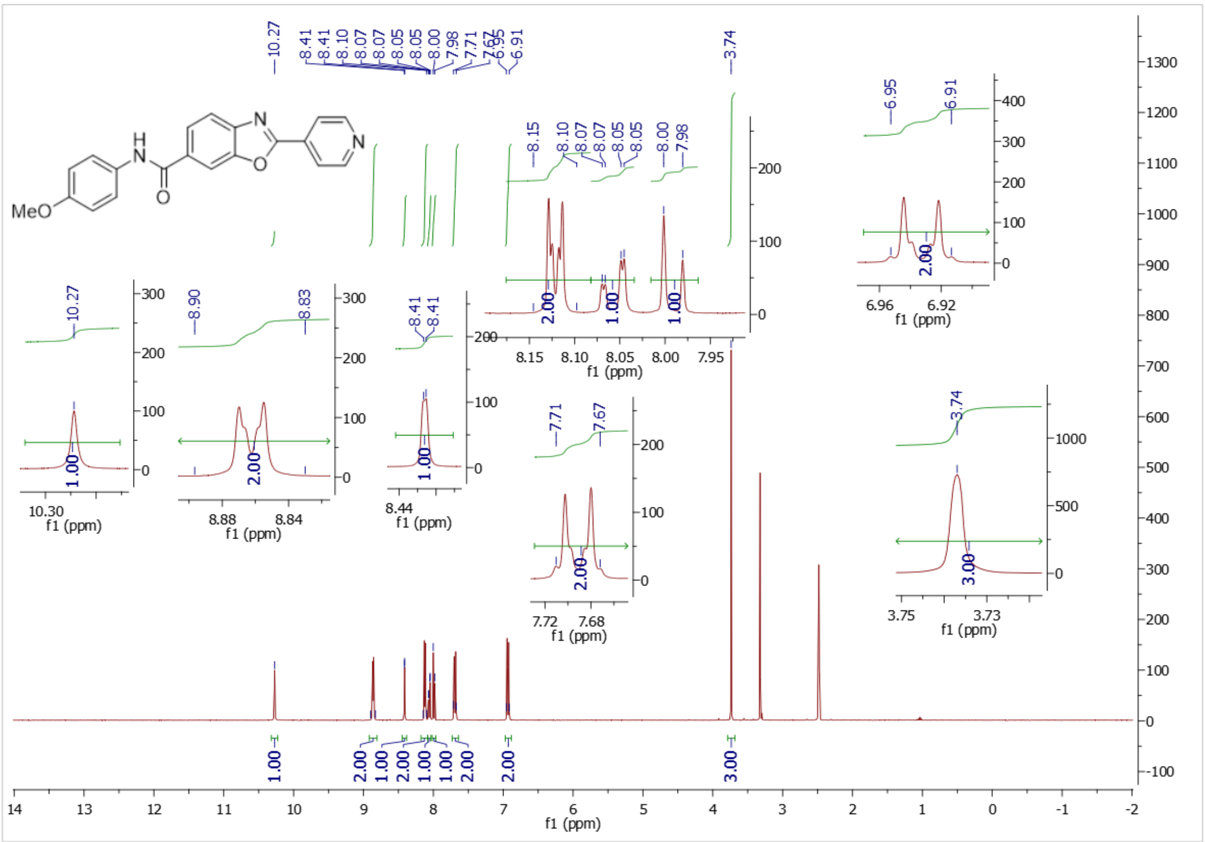

Figure S21. ^{1}H NMR spectrum of compound **11**

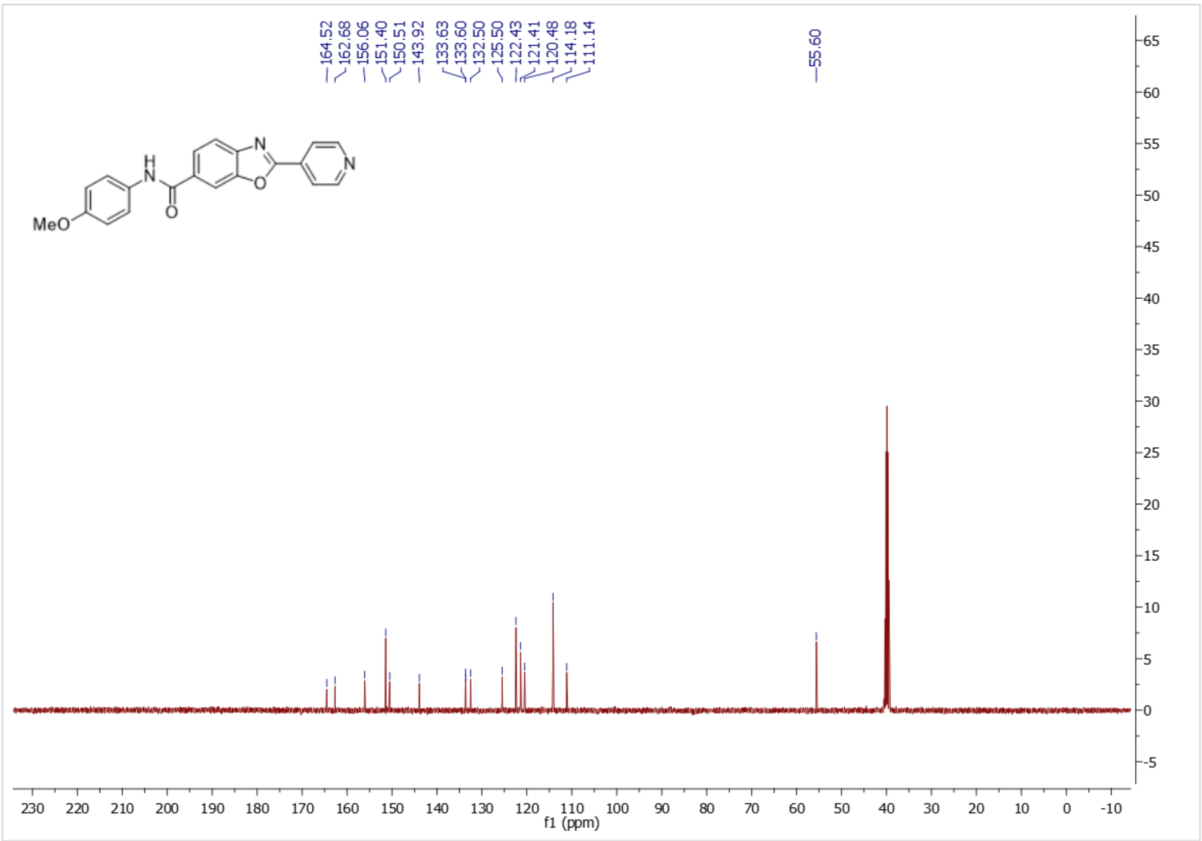

Figure S22. ^{13}C NMR spectrum of compound **11**

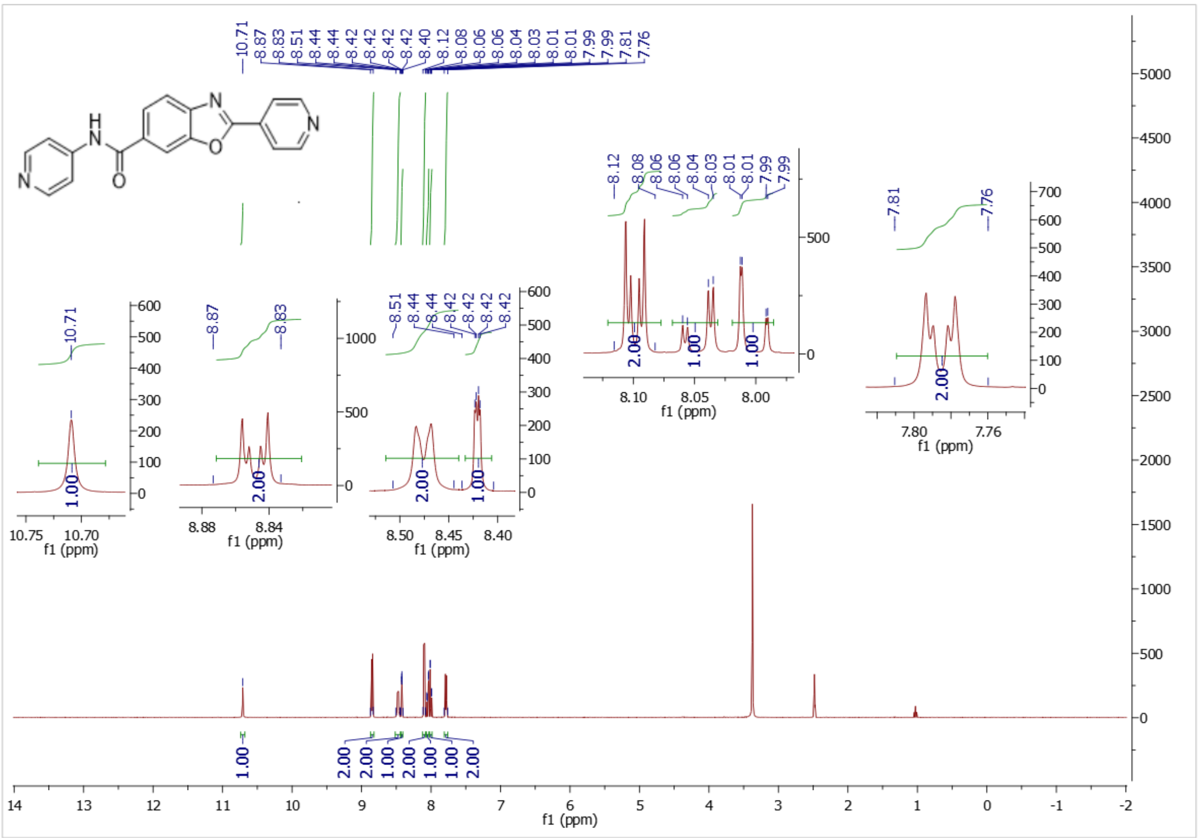

Figure S23. ^{1}H NMR spectrum of compound **12**

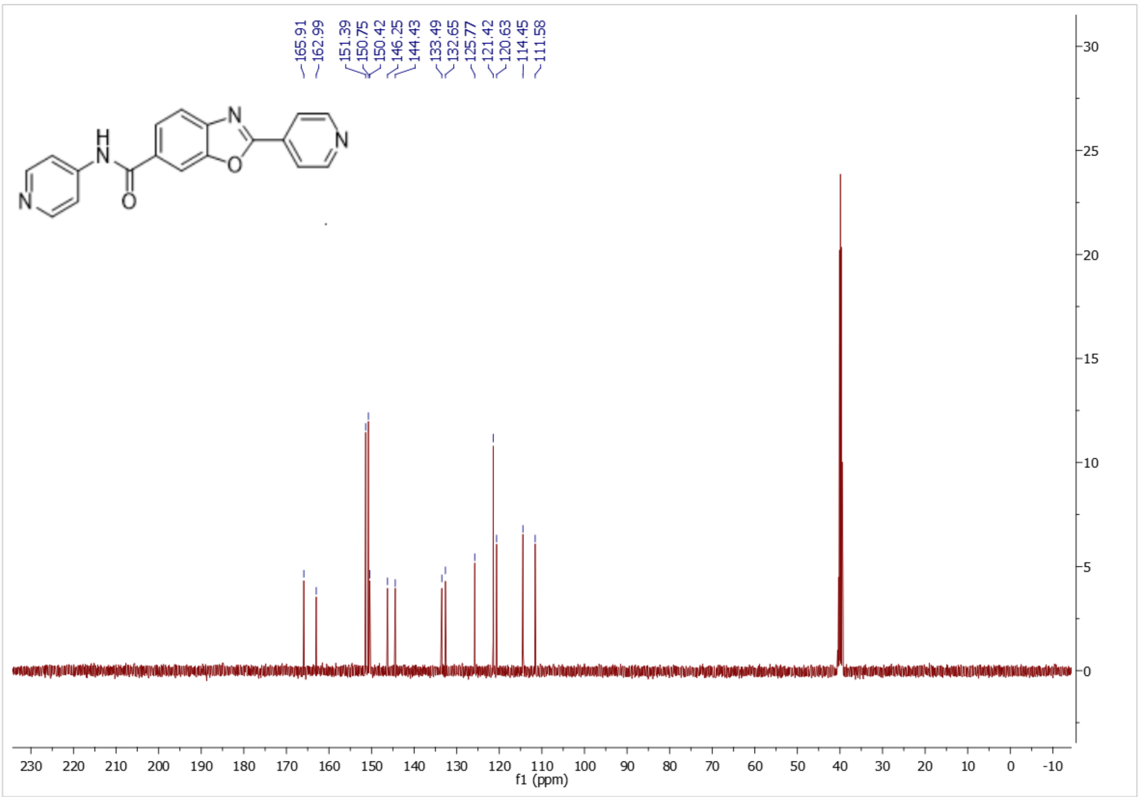

Figure S24. ^{13}C NMR spectrum of compound **12**

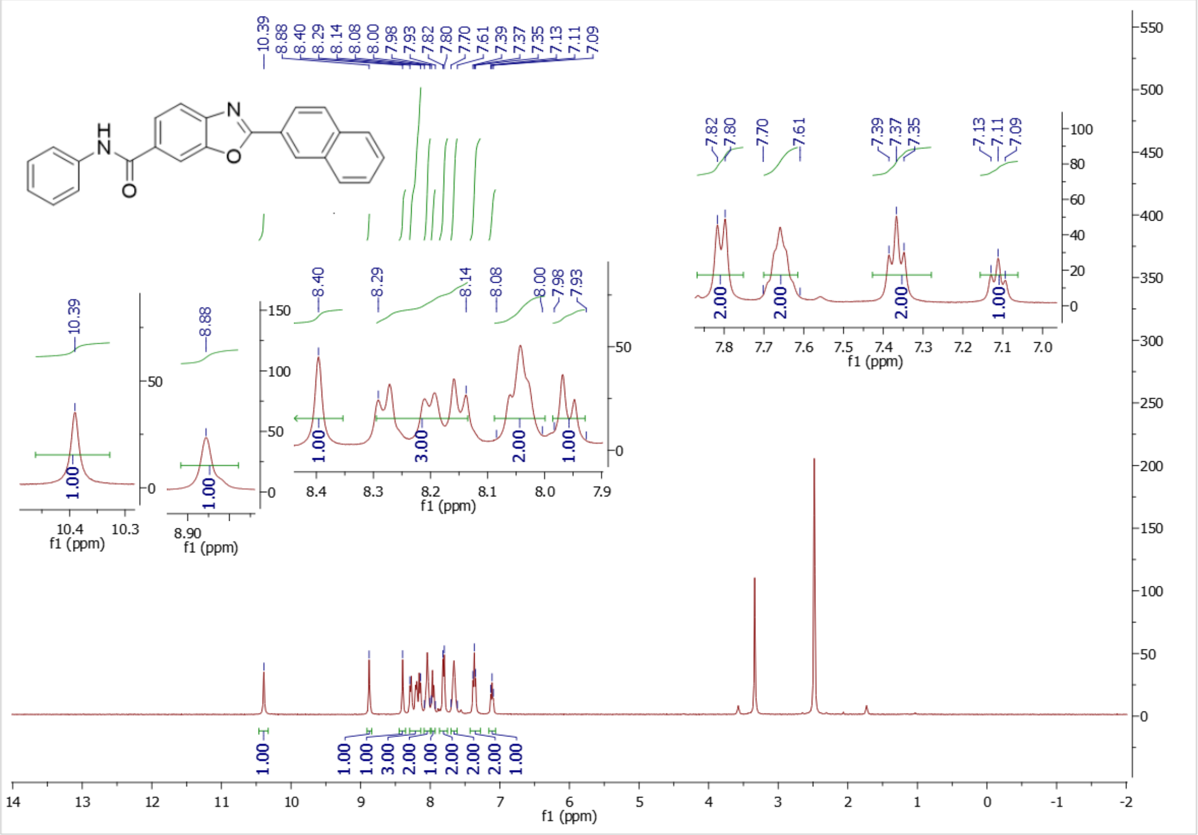

Figure S25. ^{1}H NMR spectrum of compound **13**

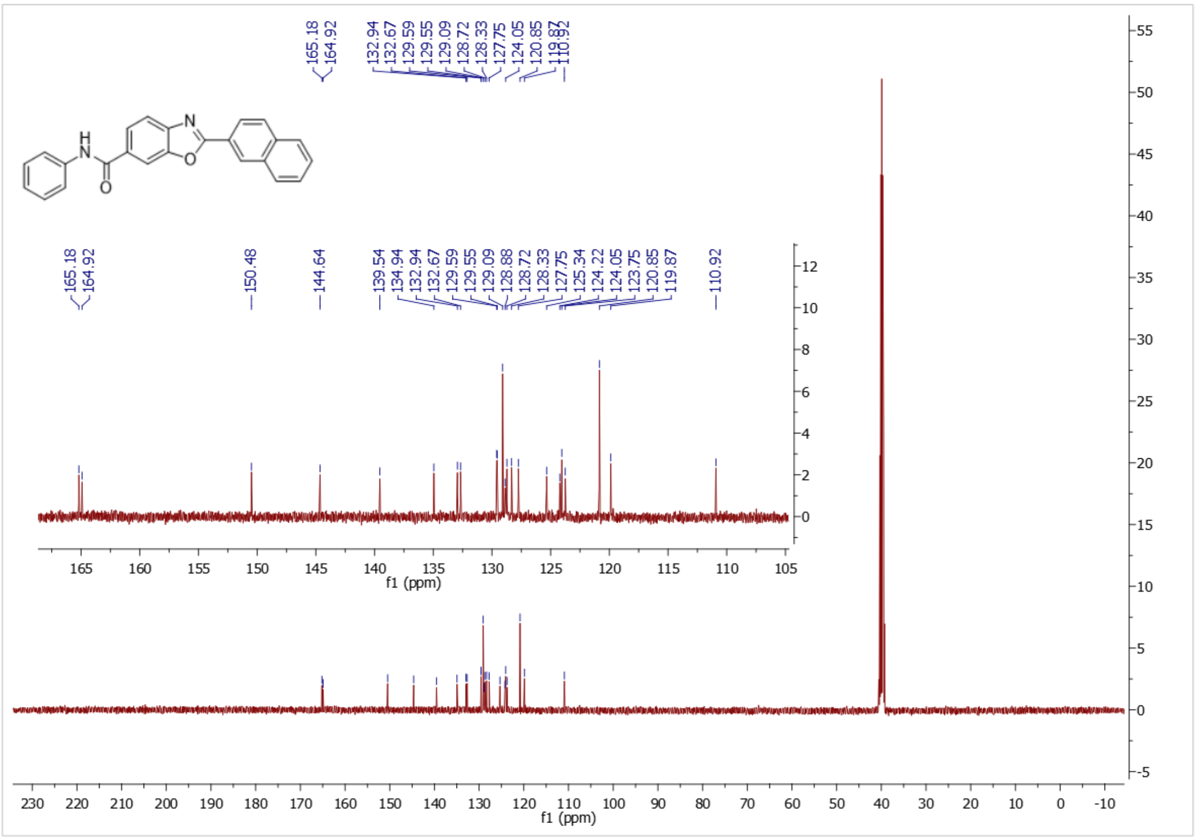

Figure S26. ^{13}C NMR spectrum of compound **13**

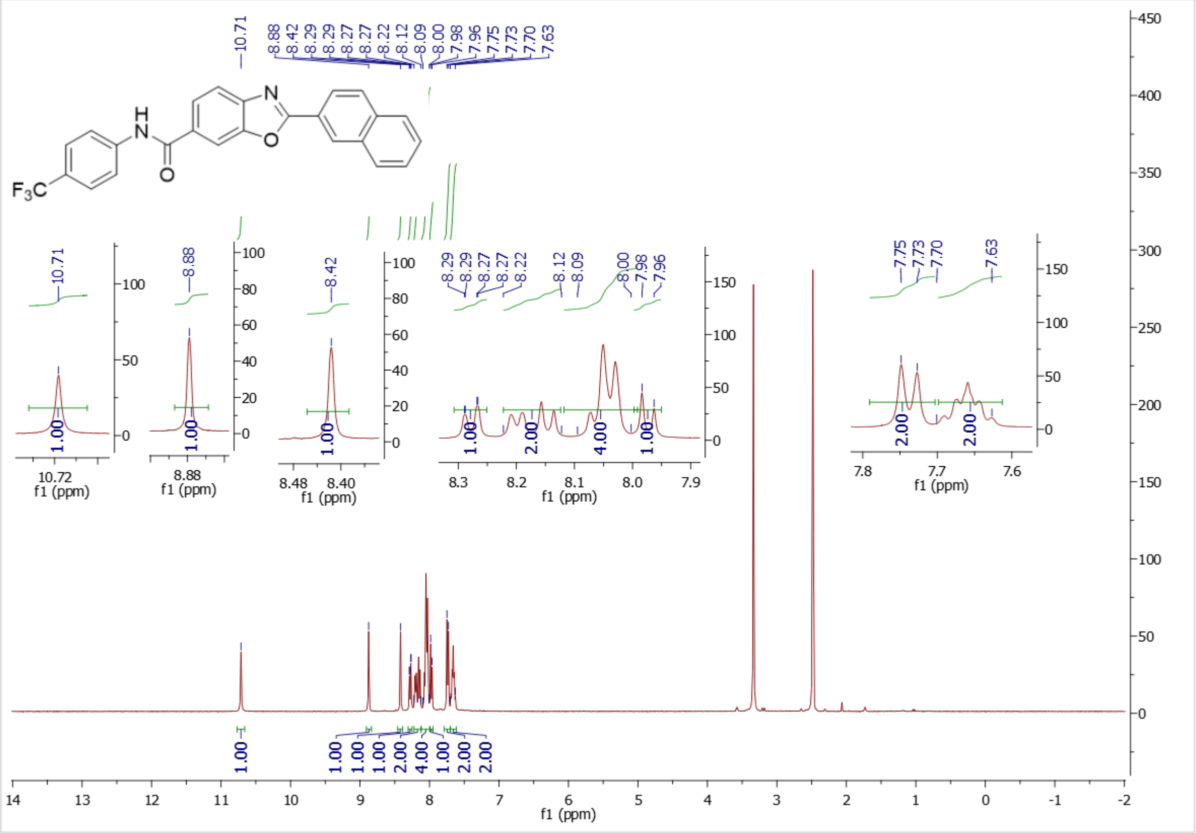

Figure S27. ^{1}H NMR spectrum of compound **14**

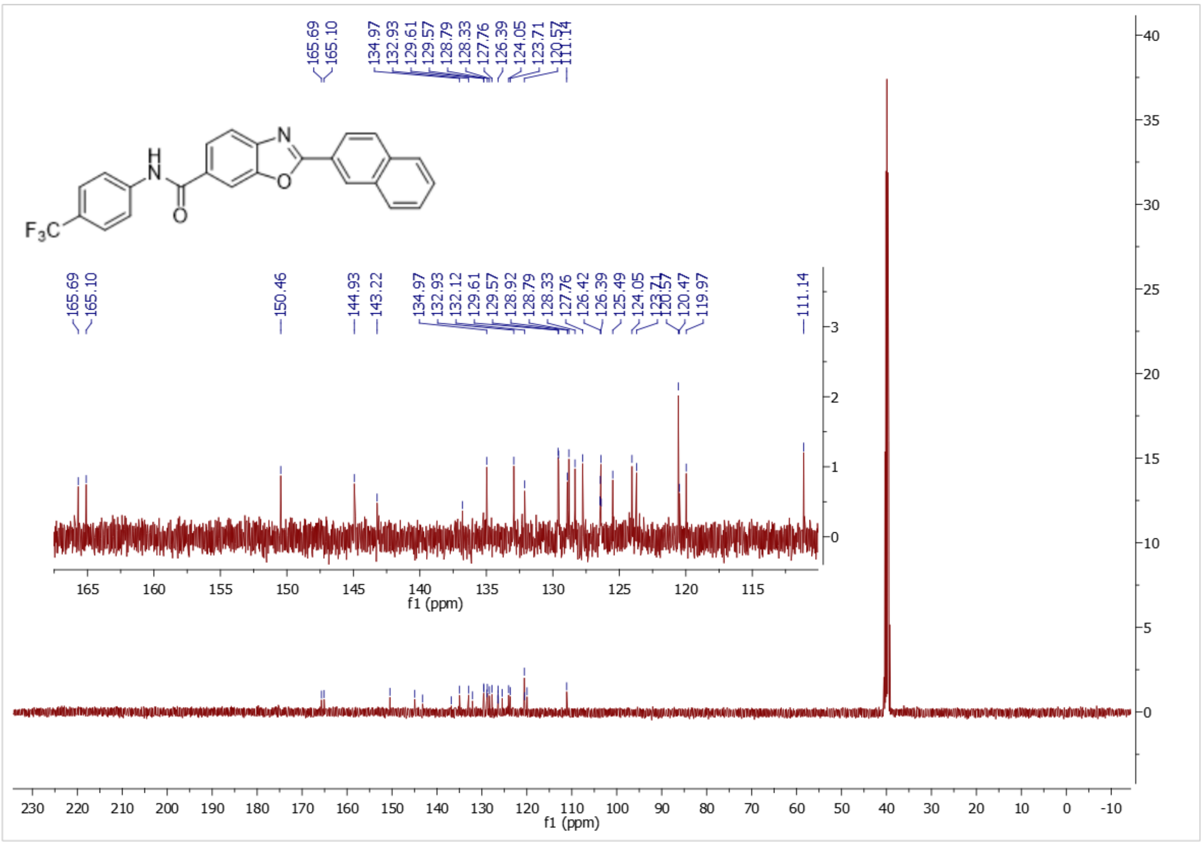

Figure S28. ^{13}C NMR spectrum of compound **14**

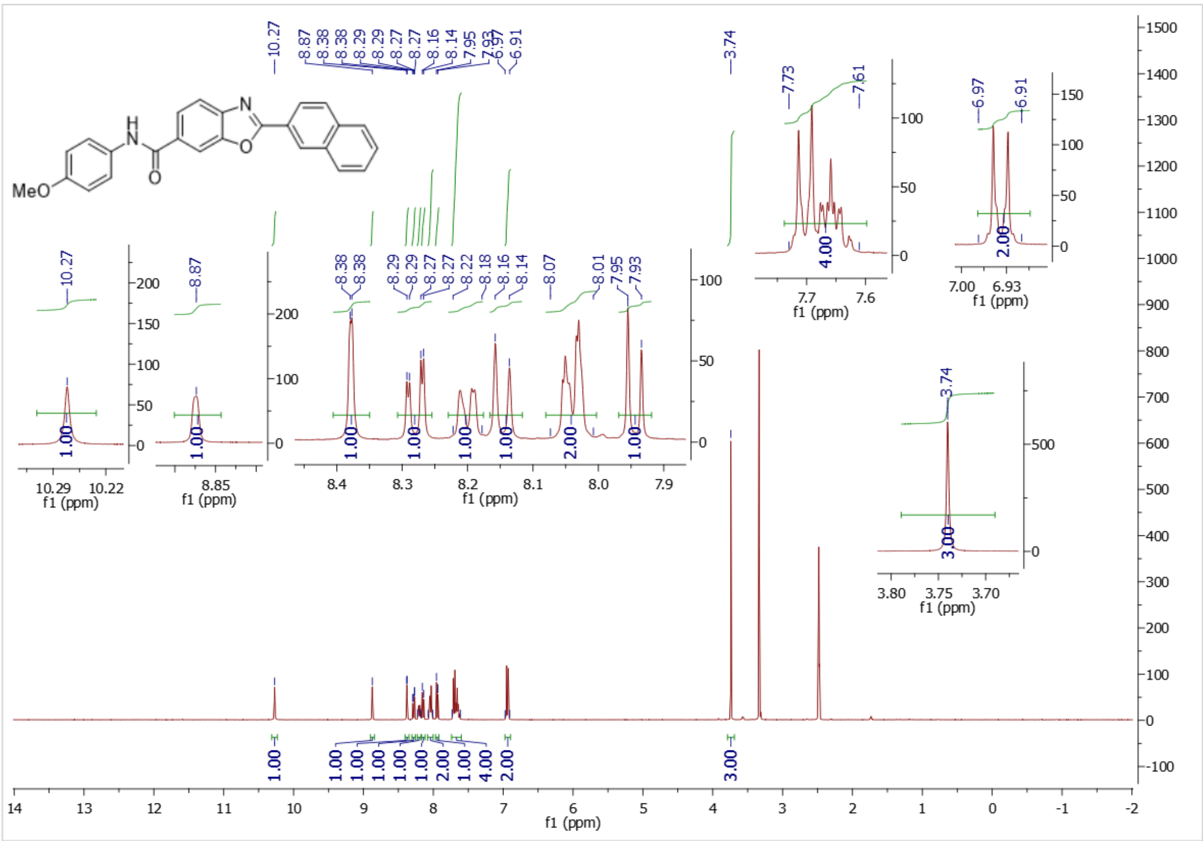

Figure S29. ^{1}H NMR spectrum of compound **15**

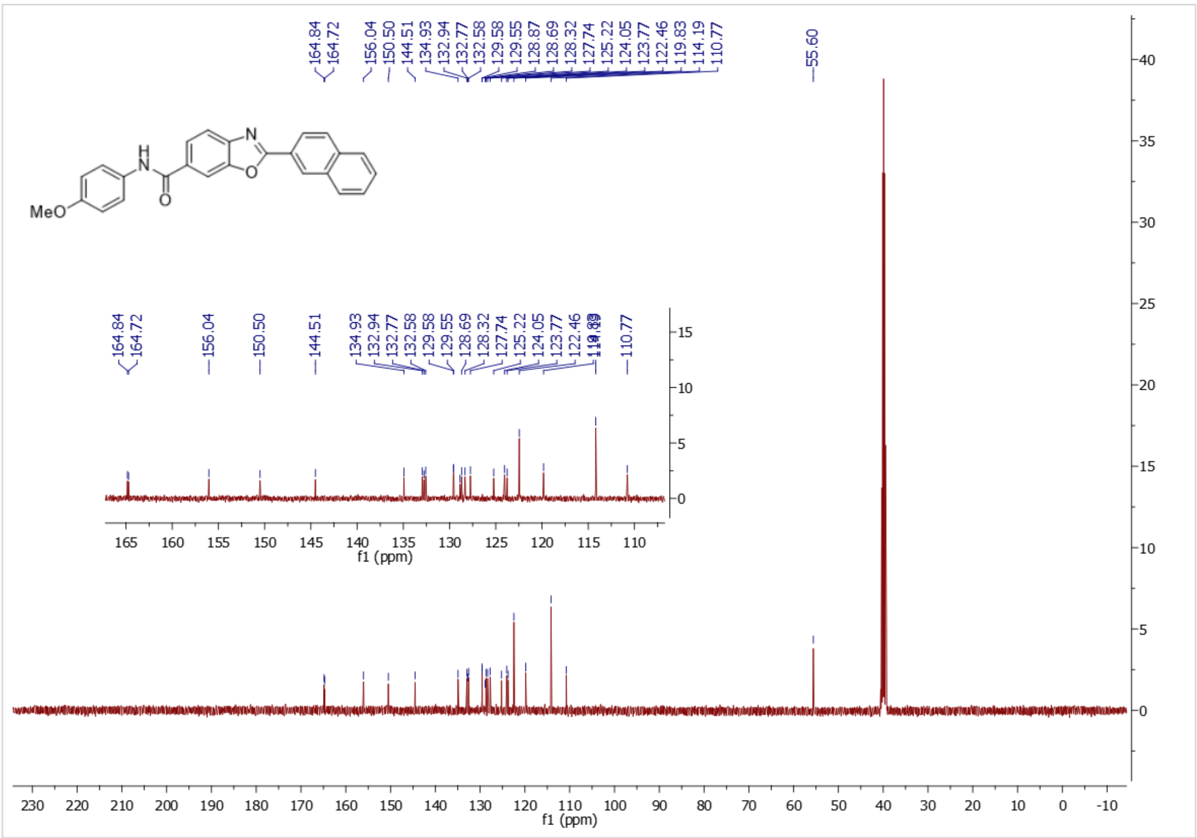

Figure S30. ^{13}C NMR spectrum of compound **15**

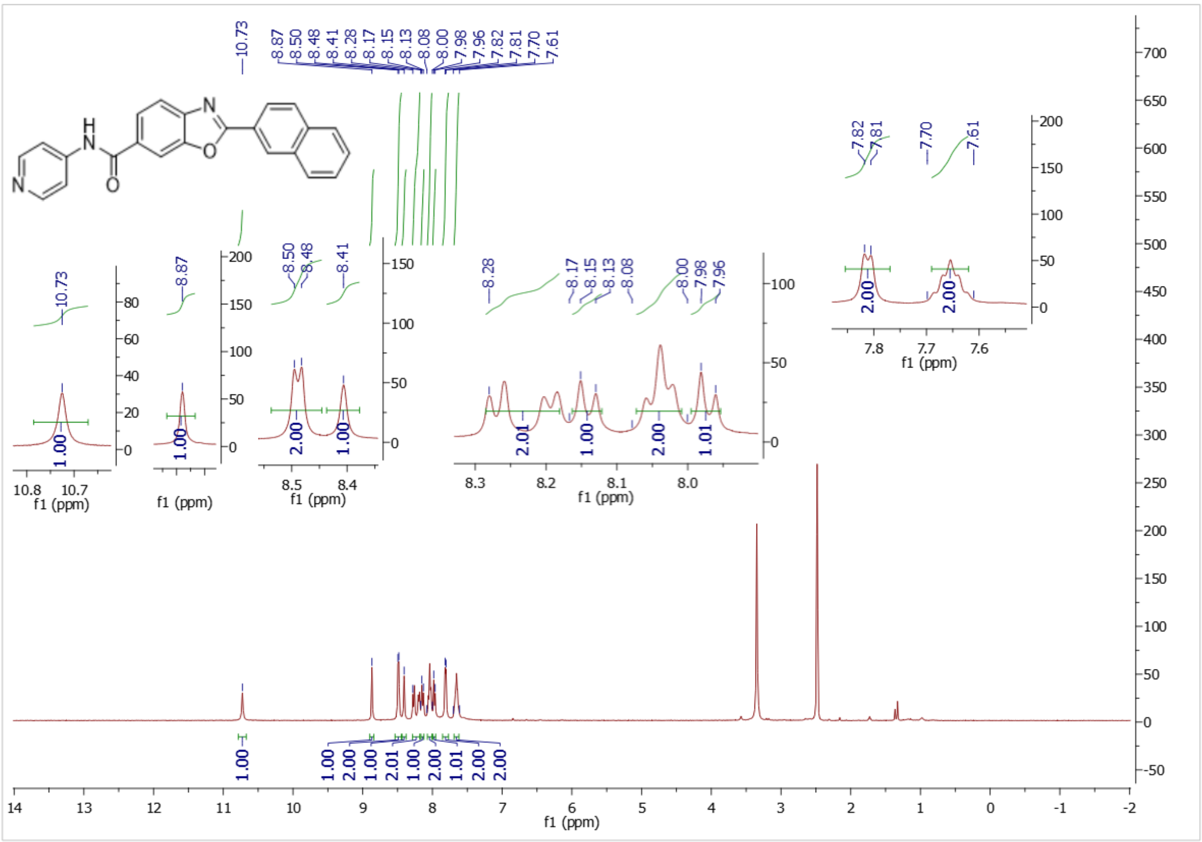

Figure S31. ^{1}H NMR spectrum of compound **16**

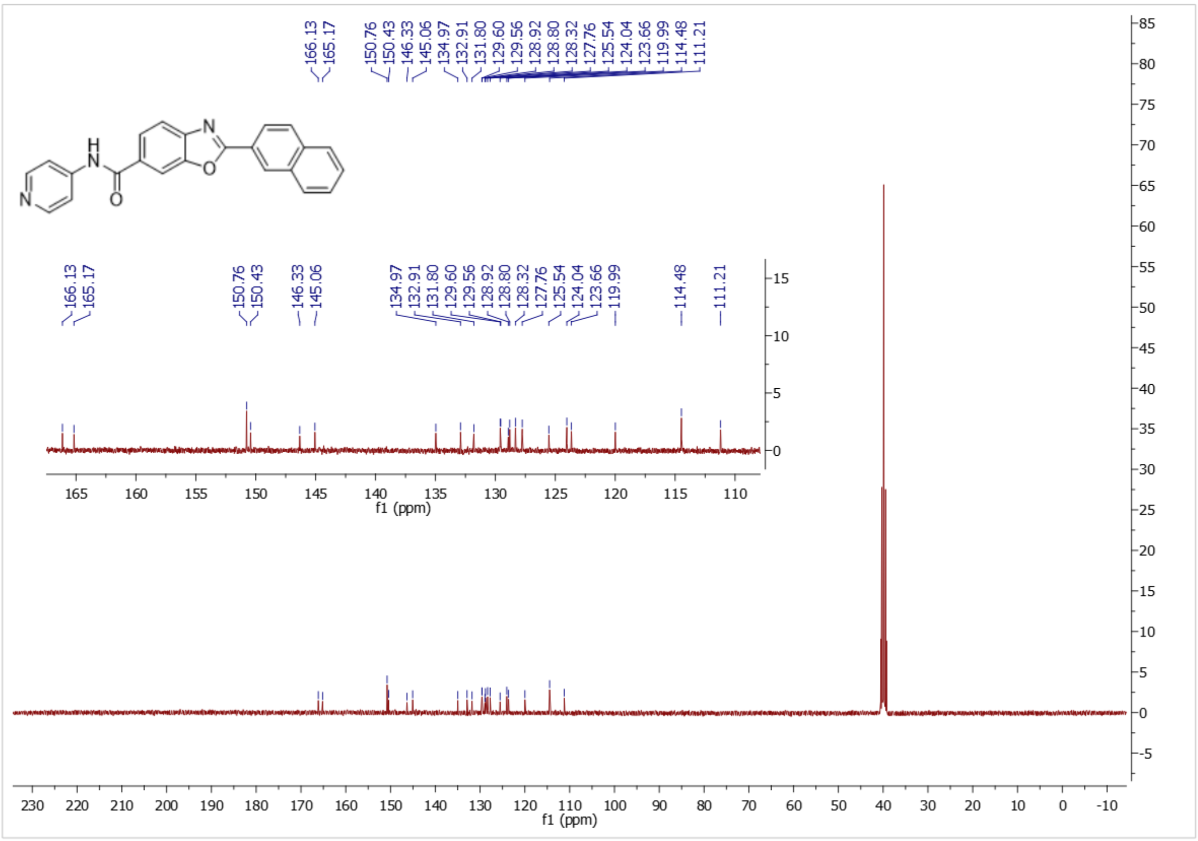

Figure S32. ^{13}C NMR spectrum of compound **16**

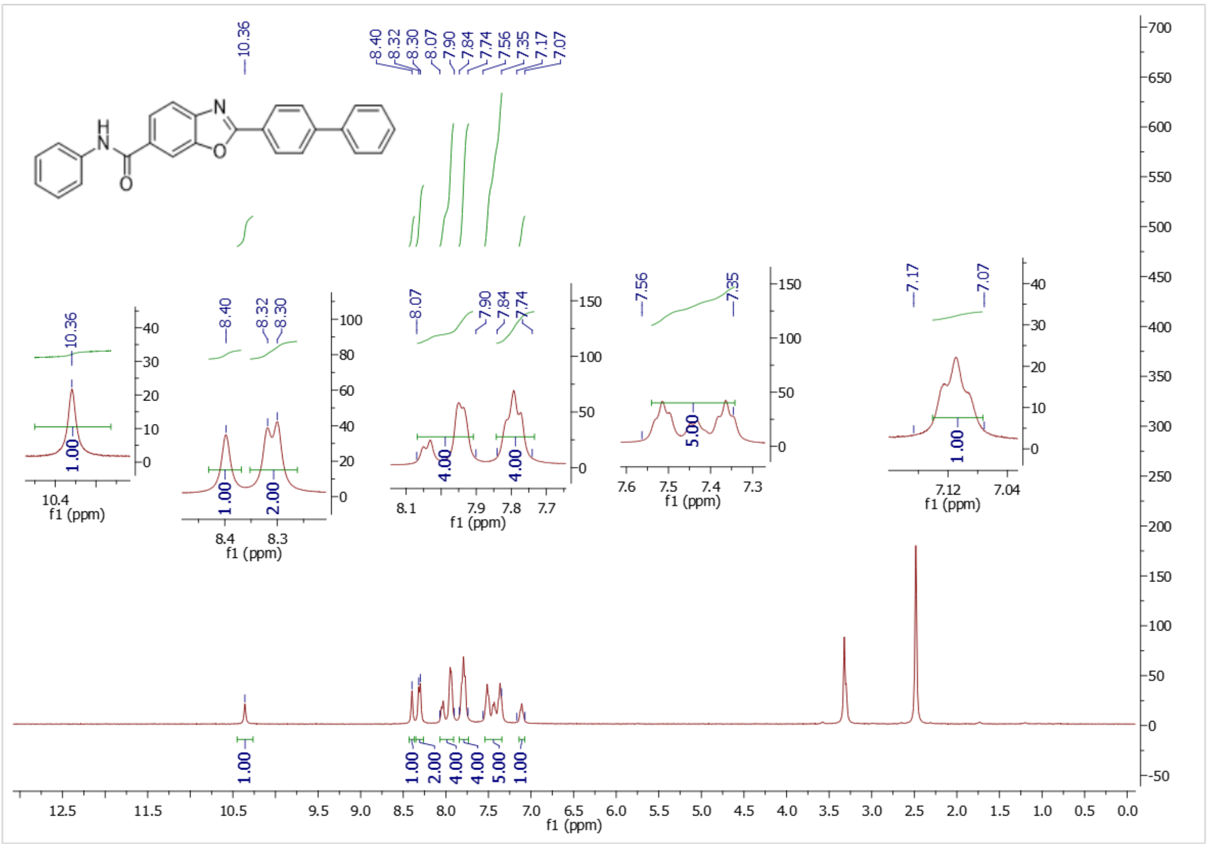

Figure S33. ^{1}H NMR spectrum of compound **17**

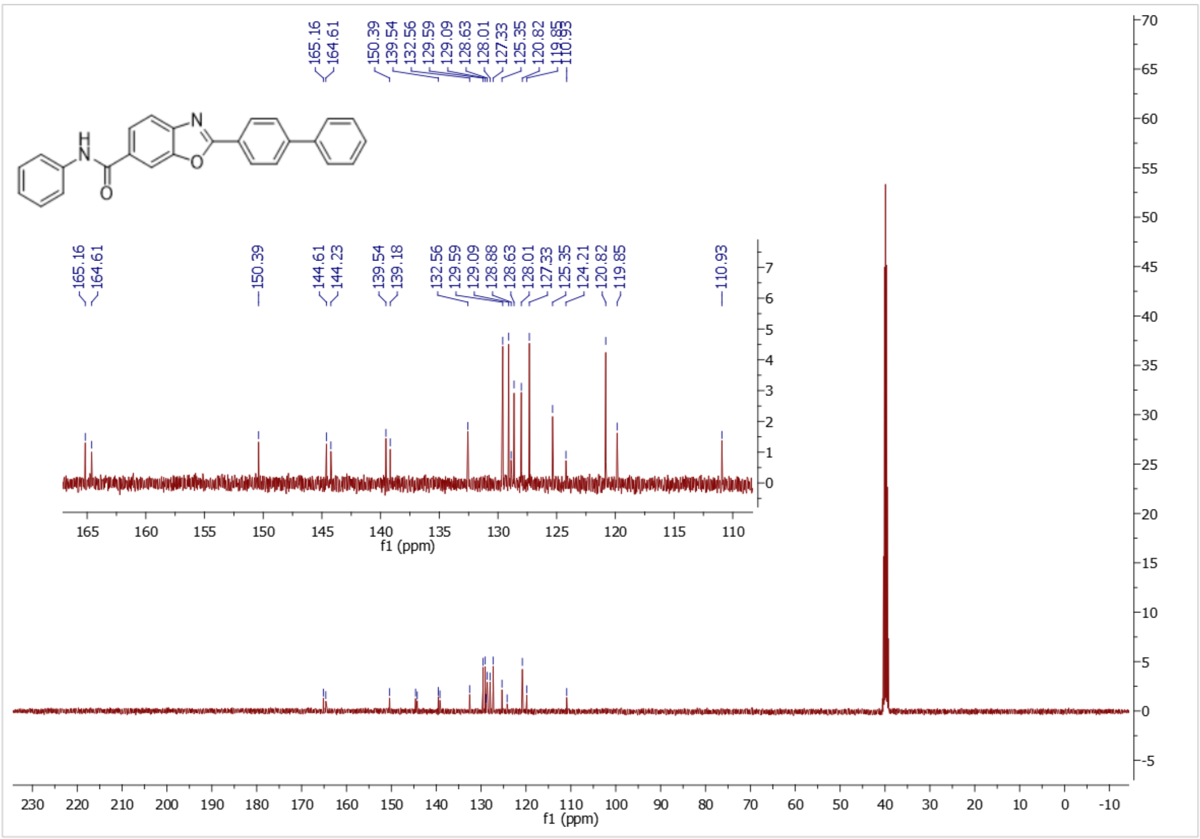

Figure S34. ^{13}C NMR spectrum of compound **17**

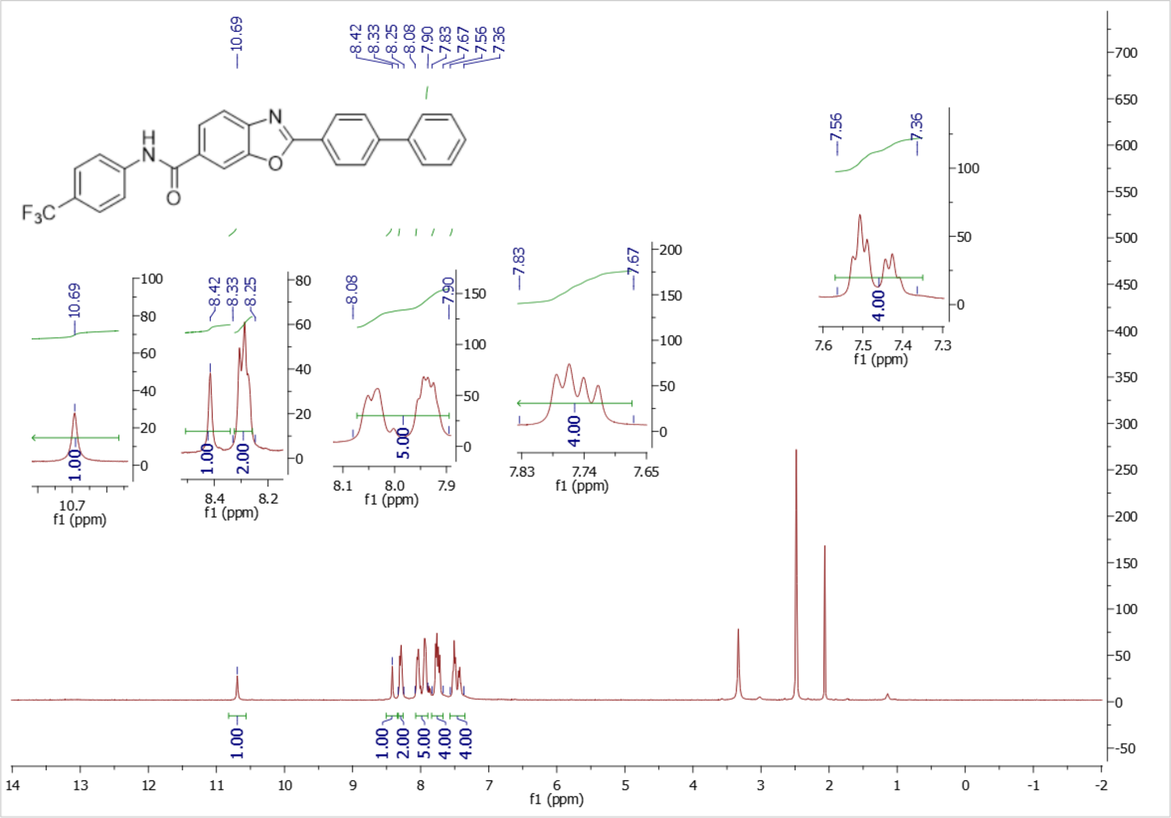

Figure S35. ^{1}H NMR spectrum of compound **18**

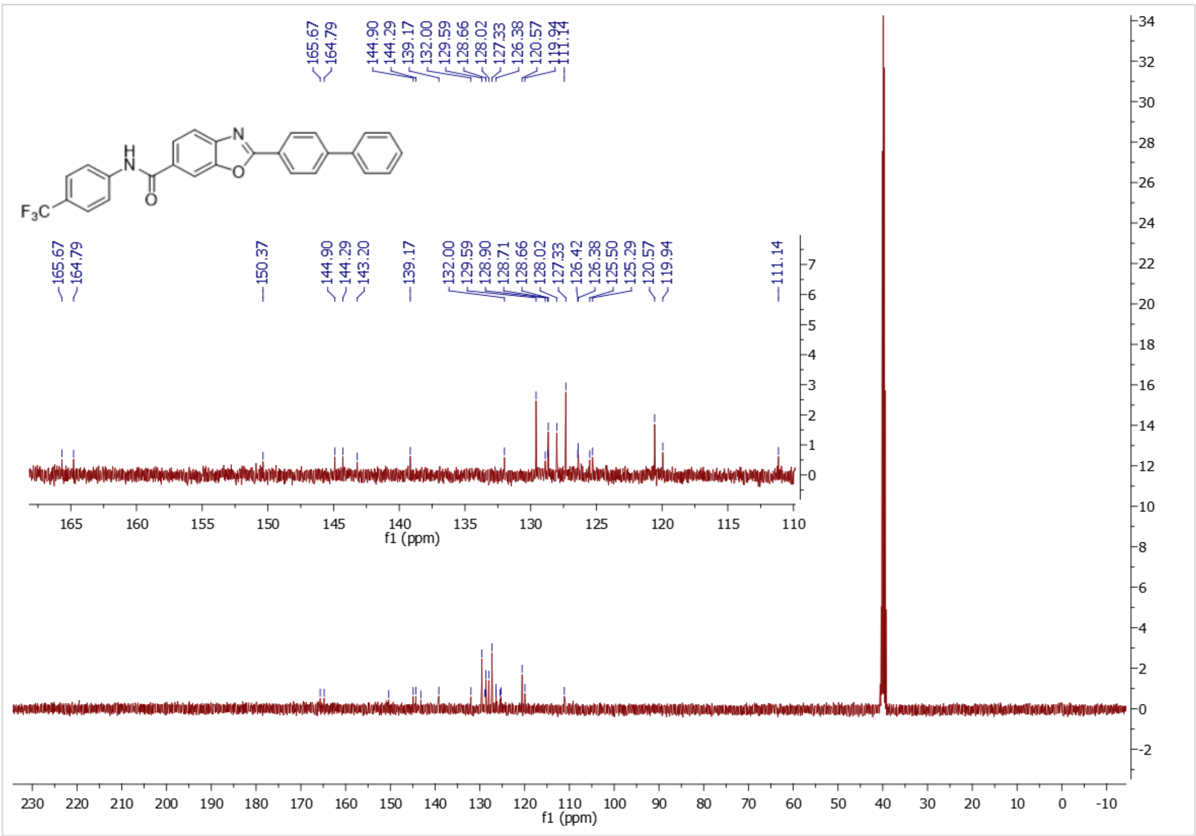

Figure S36. ^{13}C NMR spectrum of compound **18**

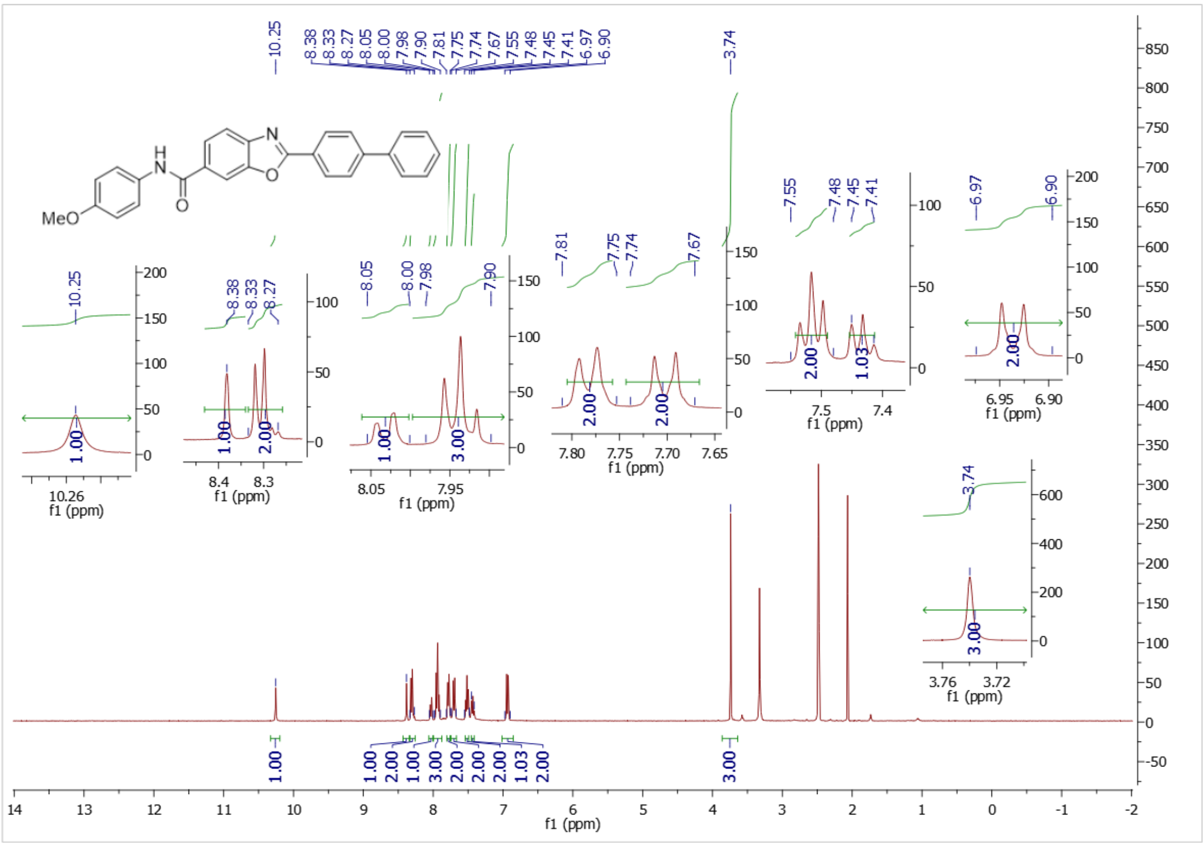

Figure S37. ^{1}H NMR spectrum of compound **19**

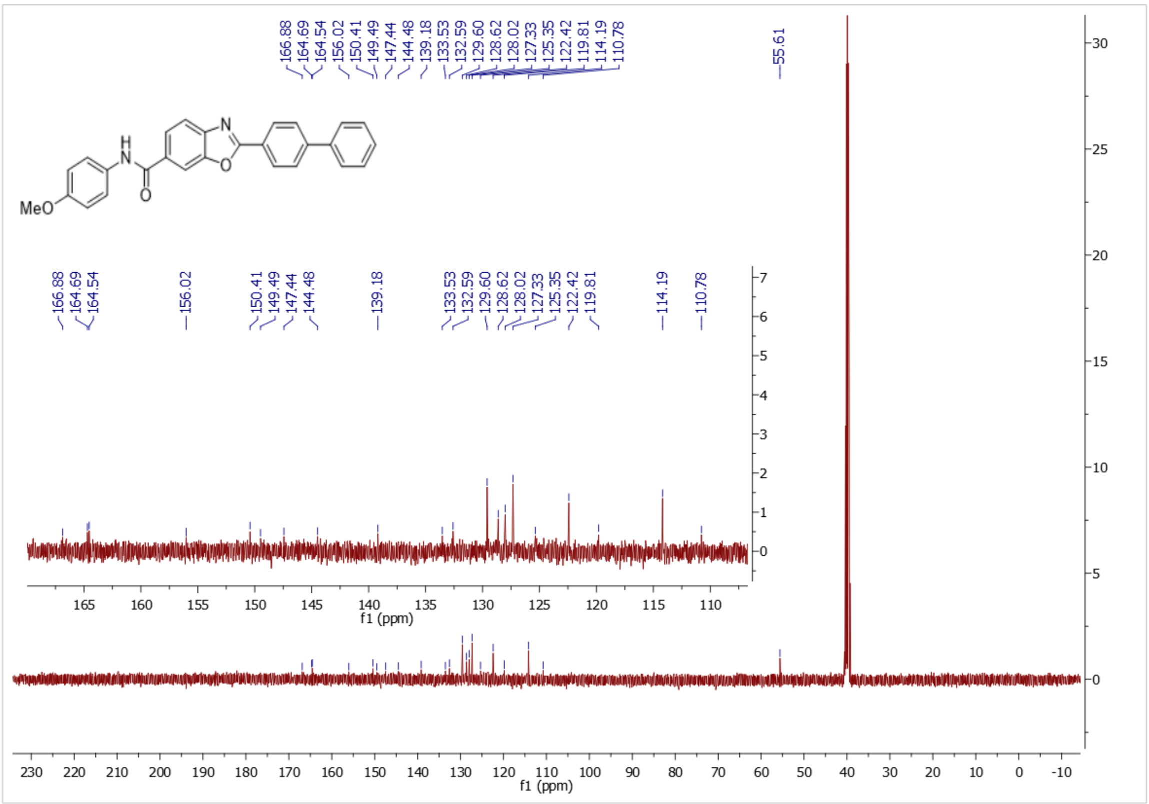

Figure S38. ^{13}C NMR spectrum of compound **19**

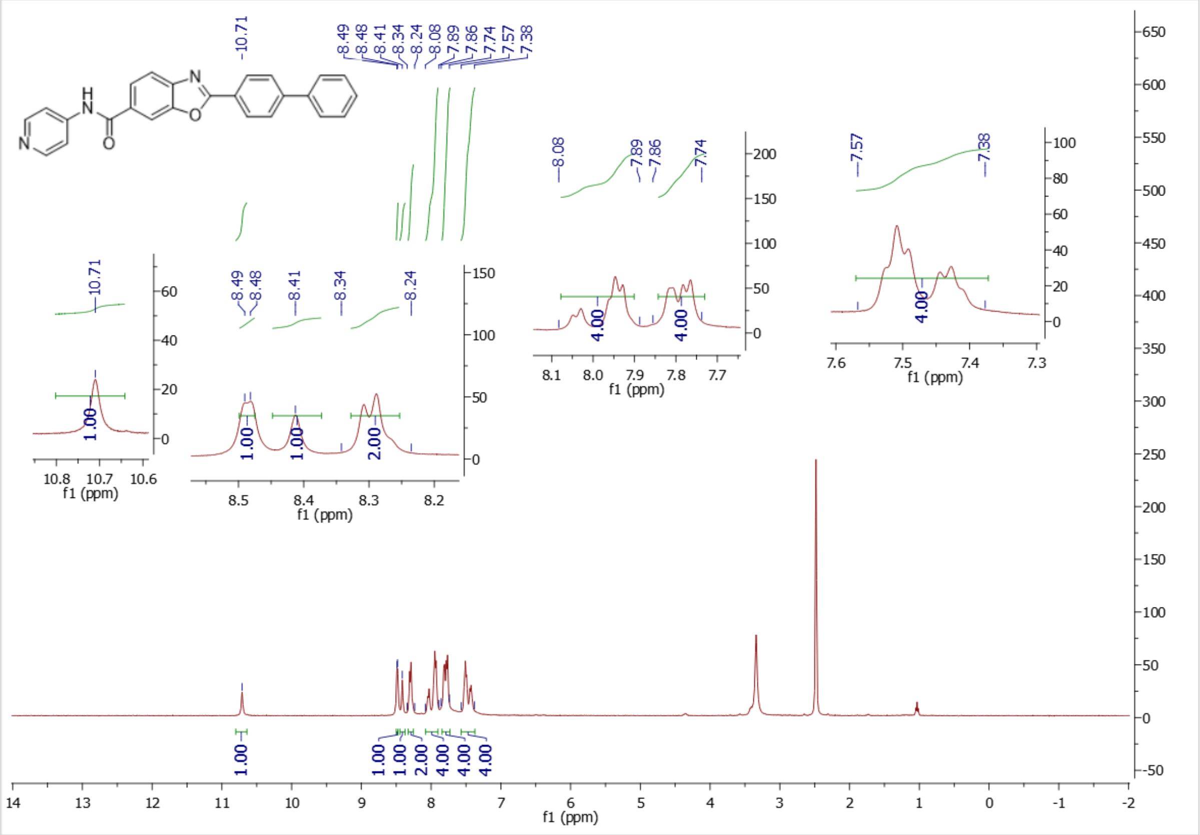

Figure S39. ^{1}H NMR spectrum of compound **20**

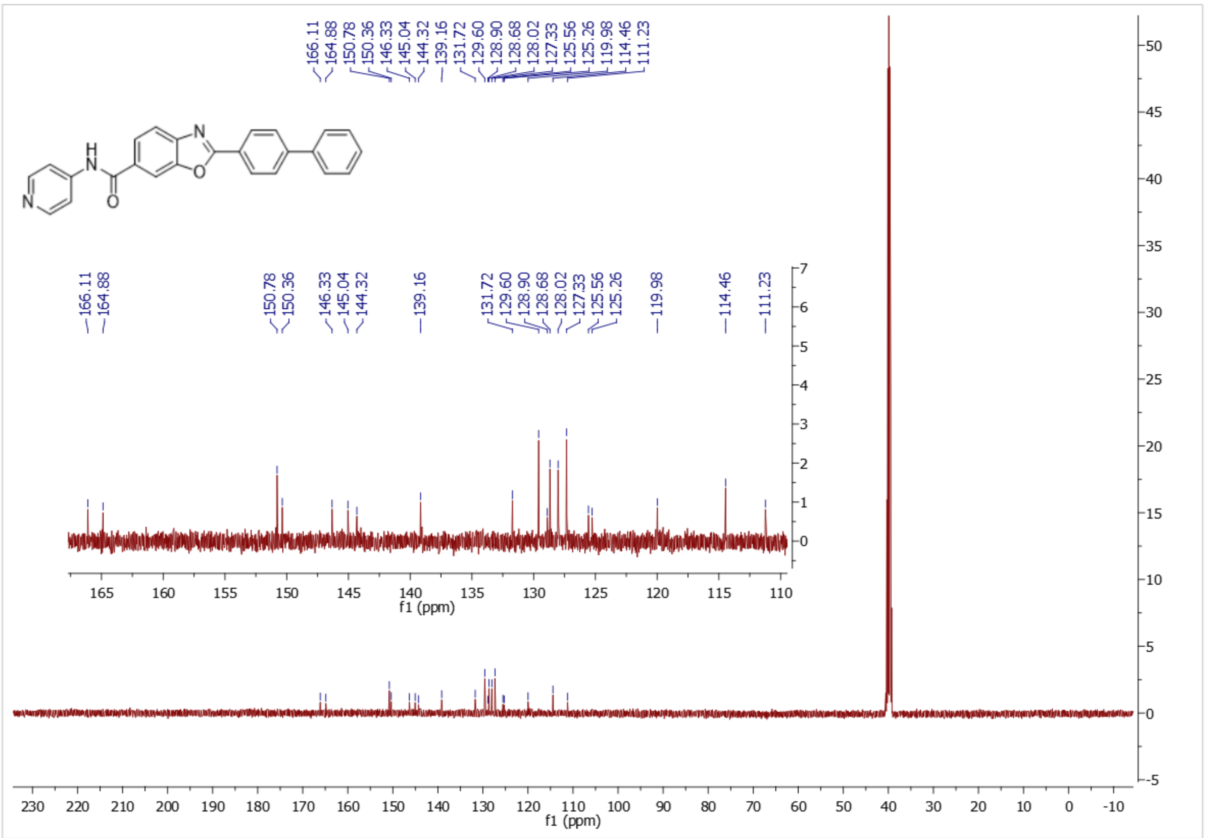

Figure S40. ^{13}C NMR spectrum of compound **20**

5. HRMS spectrum copies of compounds **1-20**

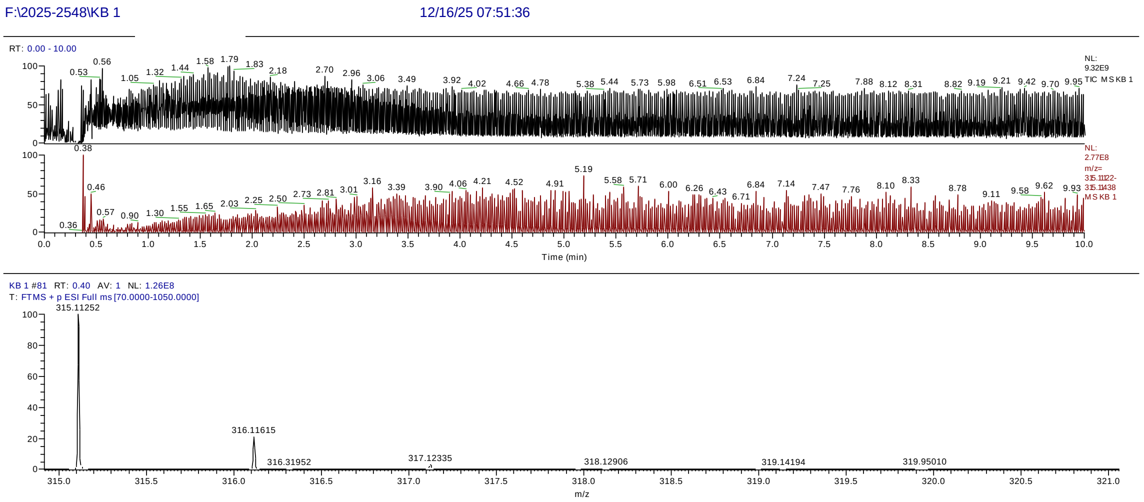

Figure S41: HRMS [M+H] spectrum of compound **1**

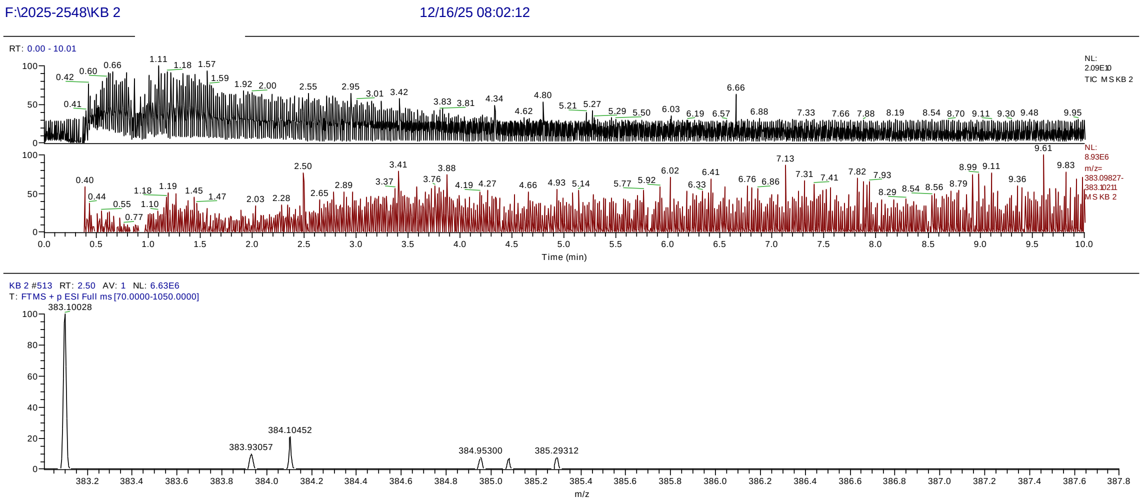

Figure S42: HRMS [M+H] spectrum of compound **2**

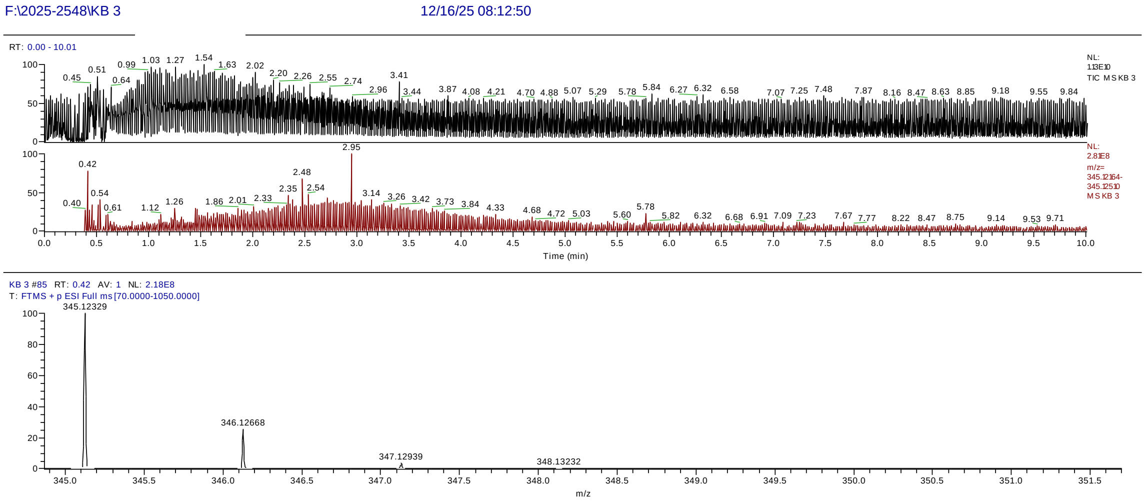

Figure S43: HRMS [M+H] spectrum of compound **3**

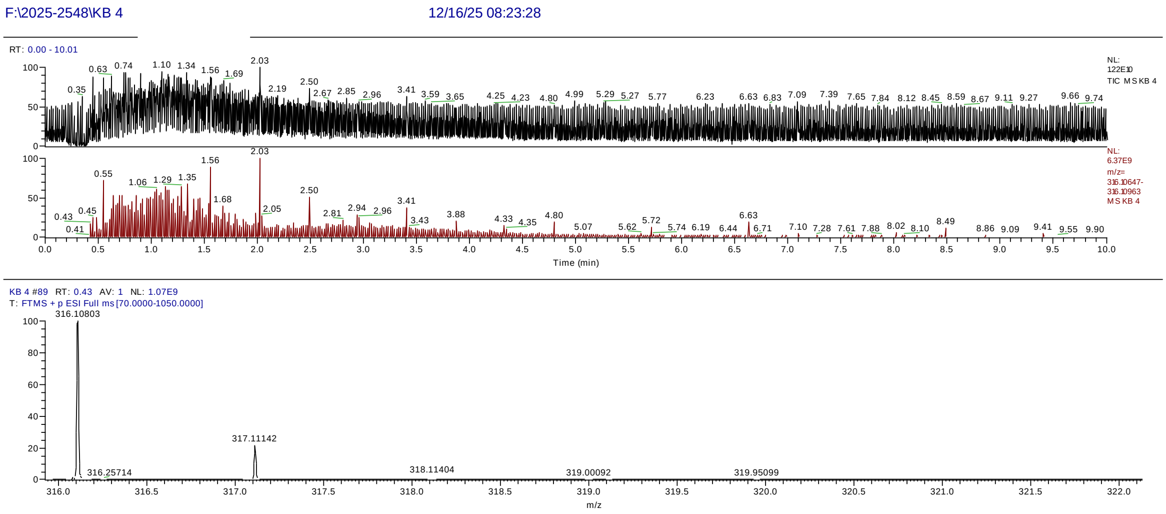

Figure S44: HRMS [M+H] spectrum of compound **4**

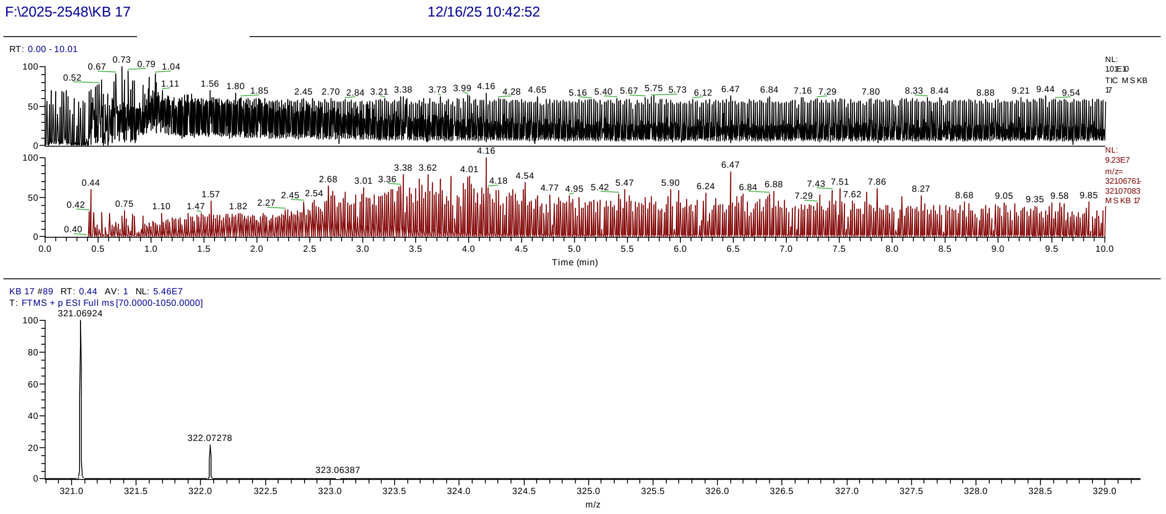

Figure S45: HRMS [M+H] spectrum of compound **5**

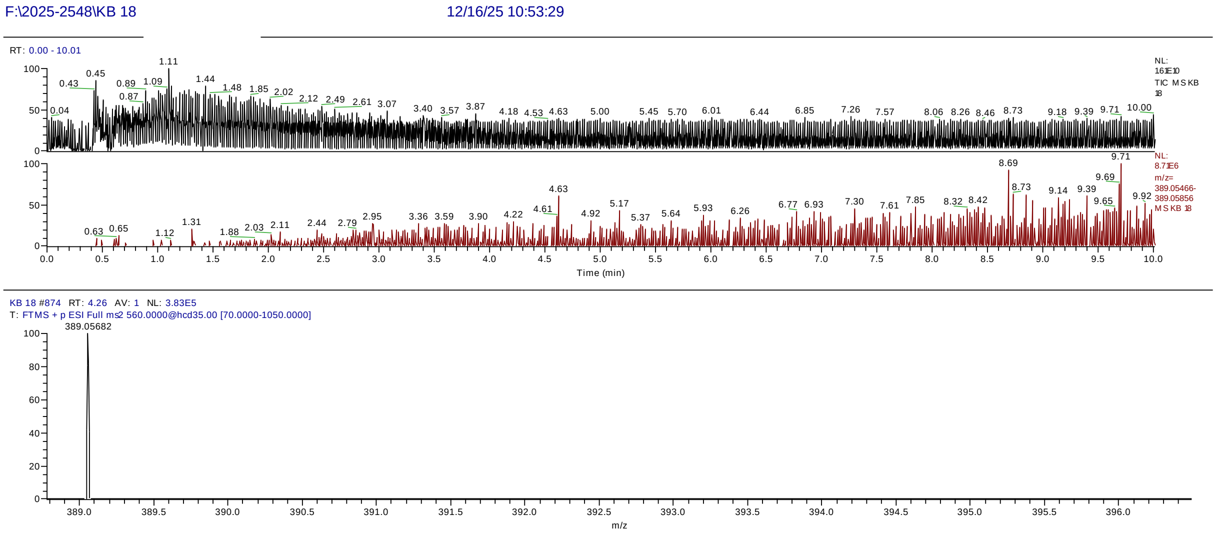

Figure S46: HRMS [M+H] spectrum of compound **6**

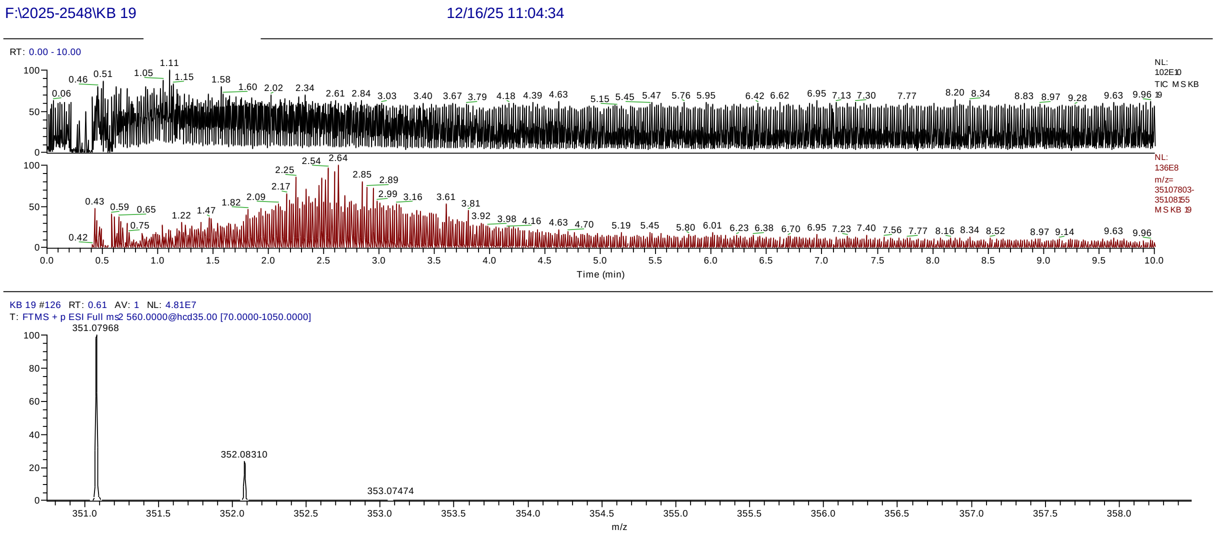

Figure S47: HRMS [M+H] spectrum of compound **7**

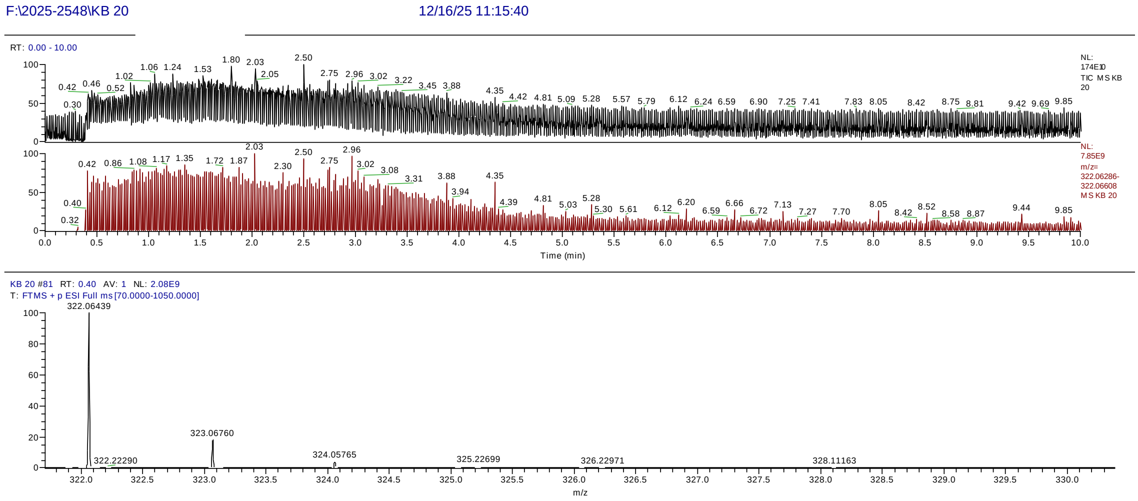

Figure S48: HRMS [M+H] spectrum of compound **8**

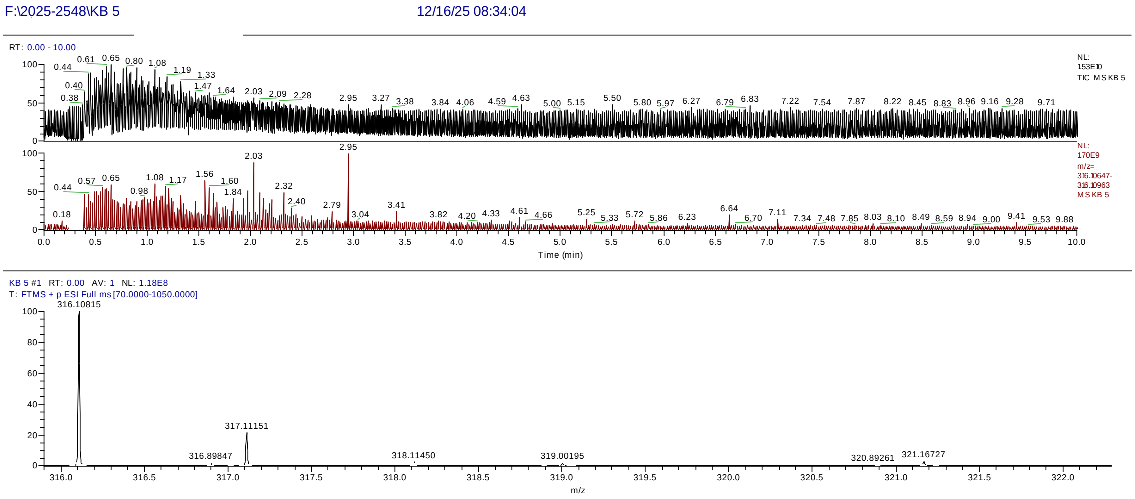

Figure S49: HRMS [M+H] spectrum of compound **9**

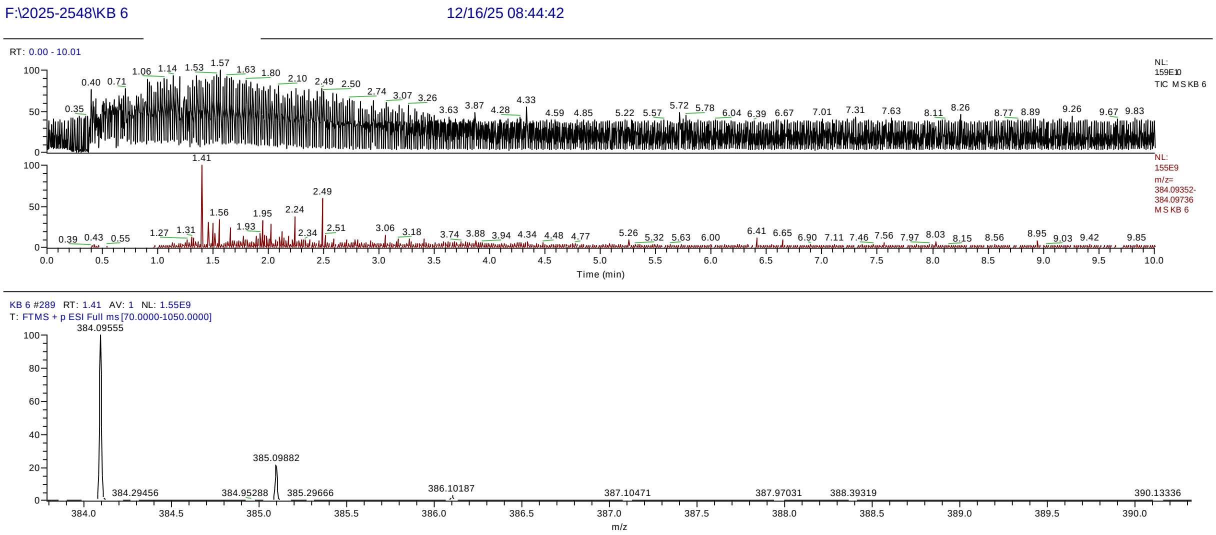

Figure S50: HRMS [M+H] spectrum of compound **10**

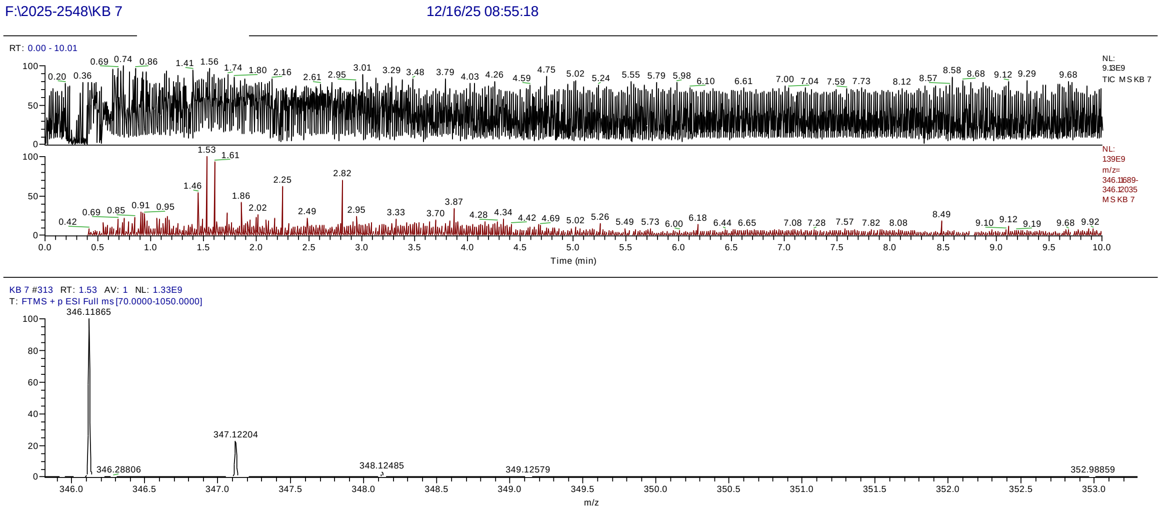

Figure S51: HRMS [M+H] spectrum of compound **11**

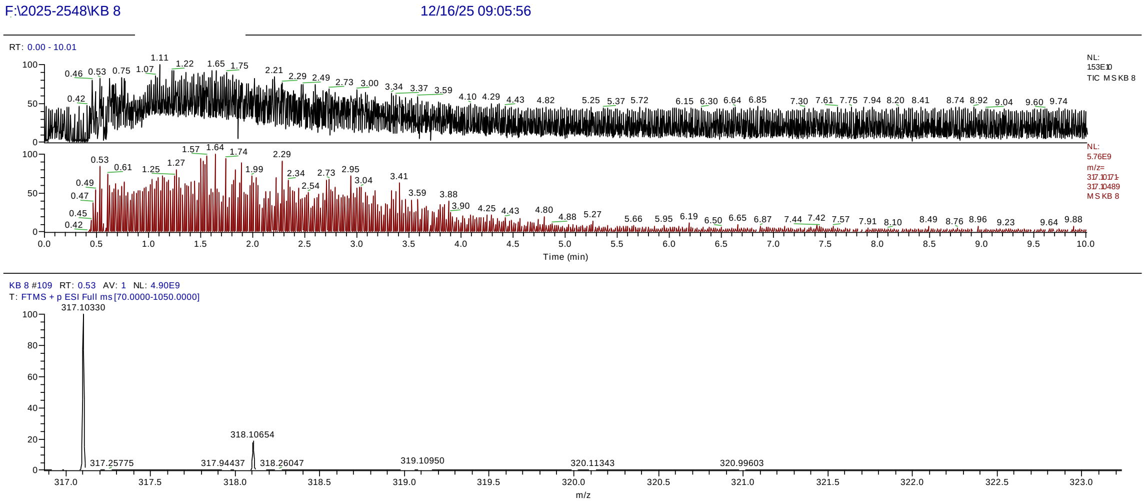

Figure S52: HRMS [M+H] spectrum of compound **12**

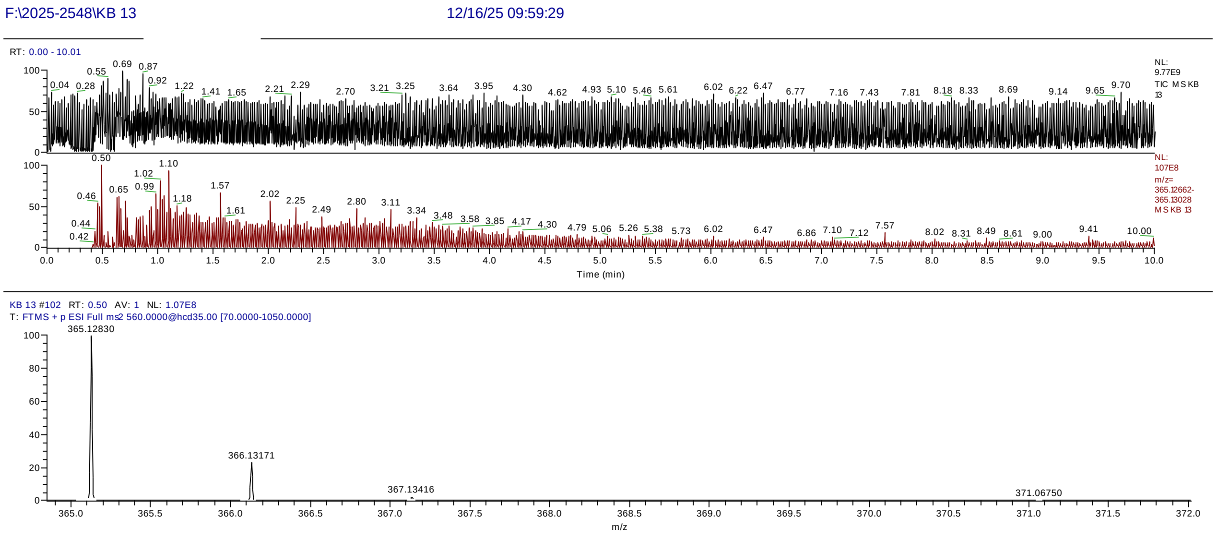

Figure S53: HRMS [M+H] spectrum of compound **13**

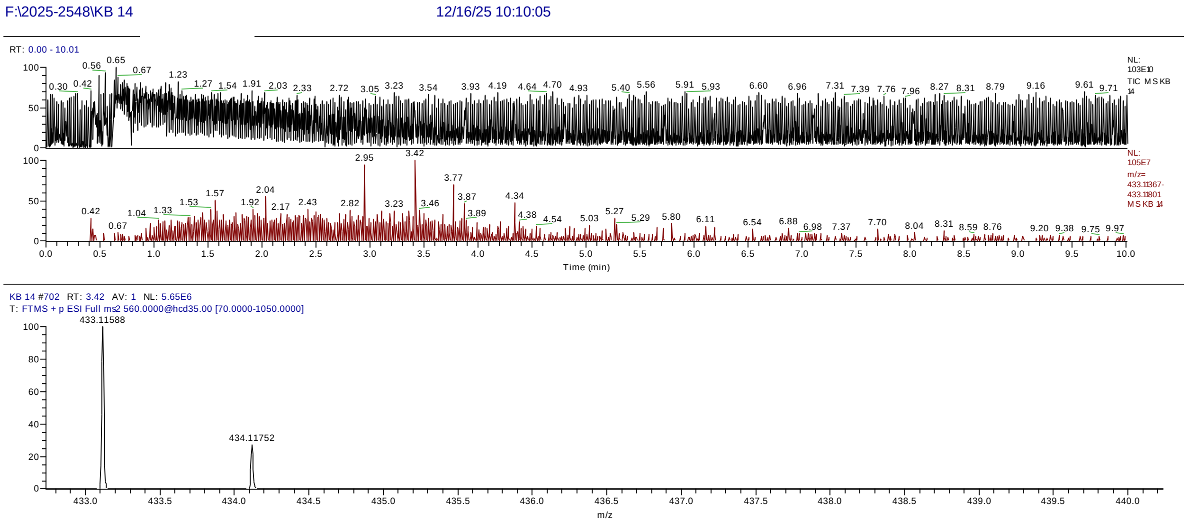

Figure S54: HRMS [M+H] spectrum of compound **14**

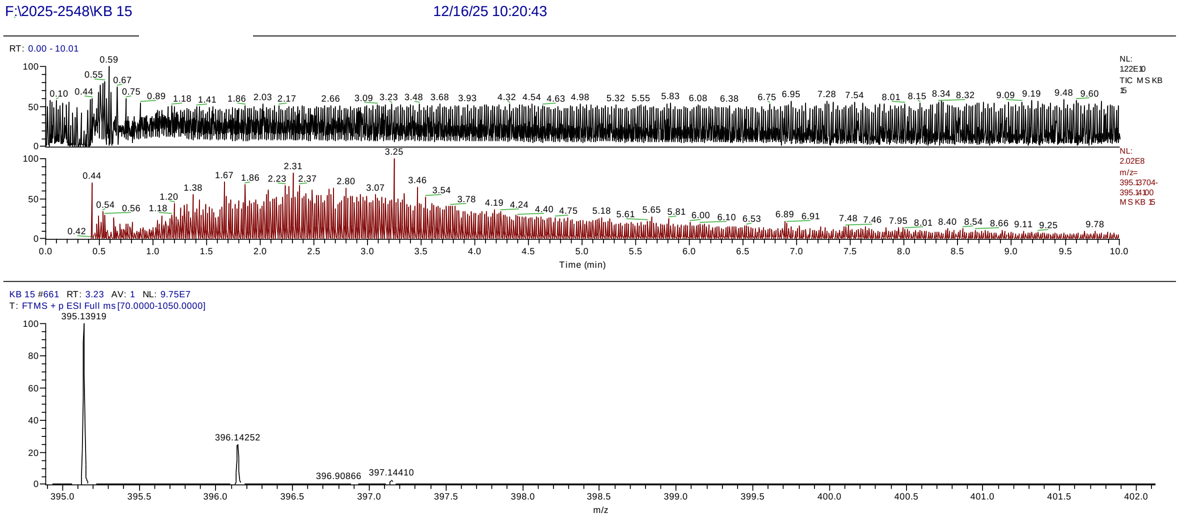

Figure S55: HRMS [M+H] spectrum of compound **15**

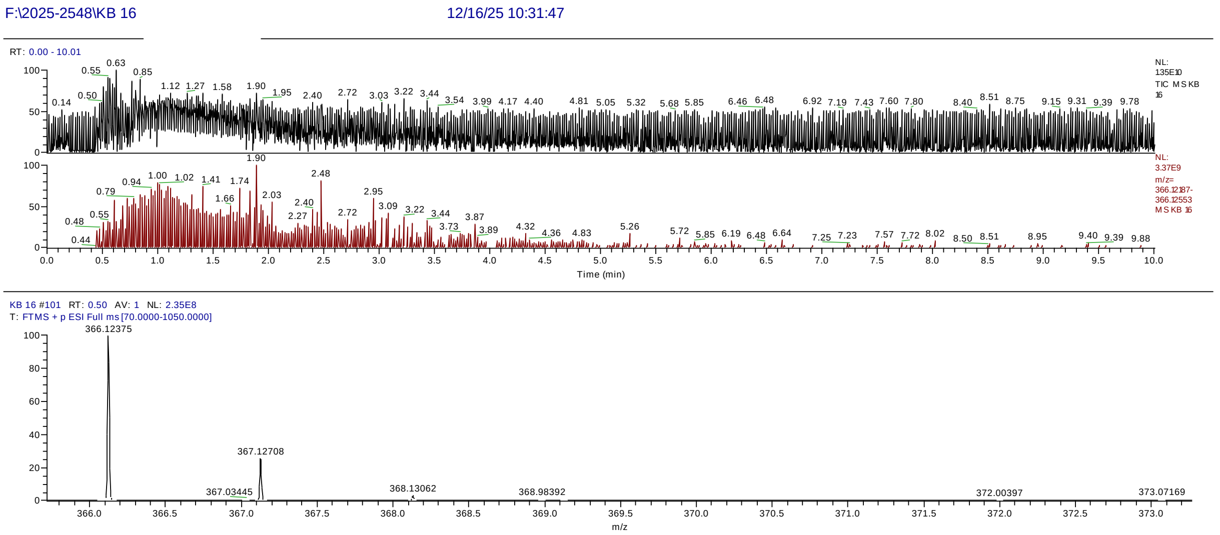

Figure S56: HRMS [M+H] spectrum of compound **16**

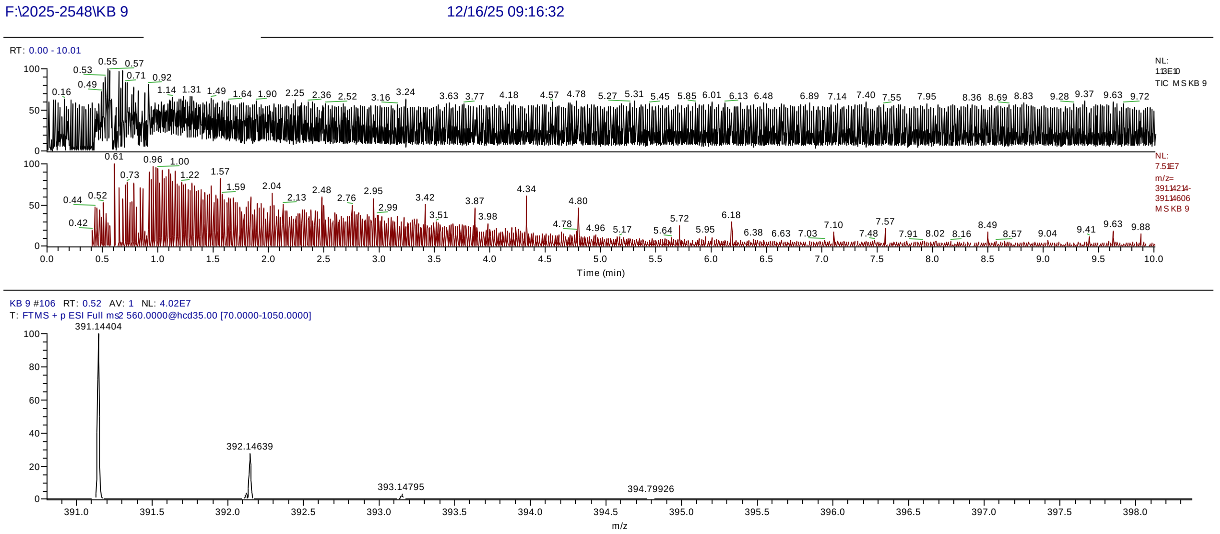

Figure S57: HRMS [M+H] spectrum of compound **17**

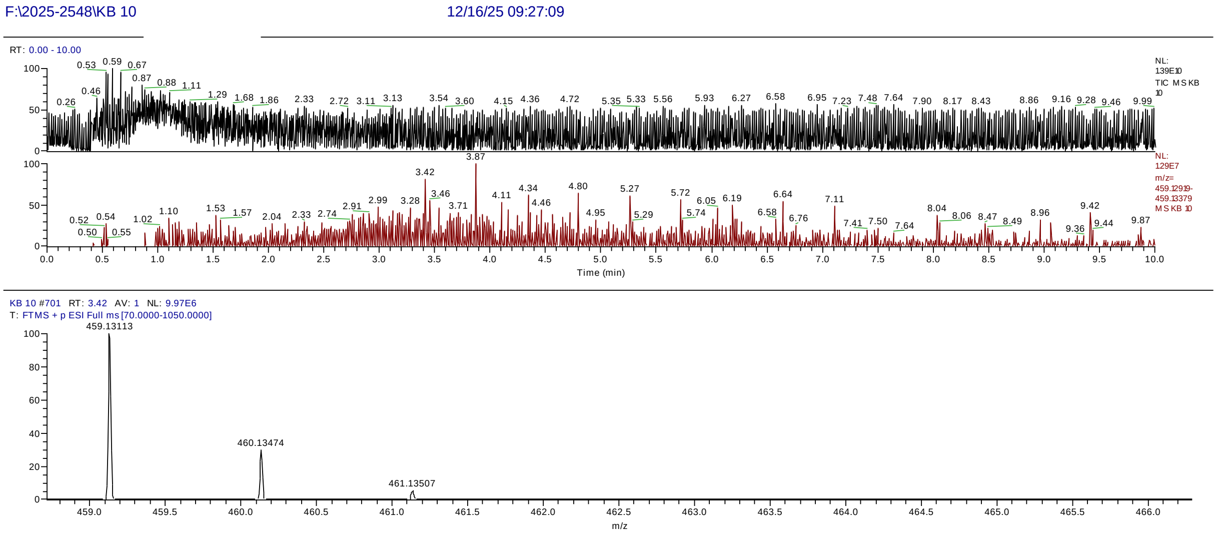

Figure S58: HRMS [M+H] spectrum of compound **18**

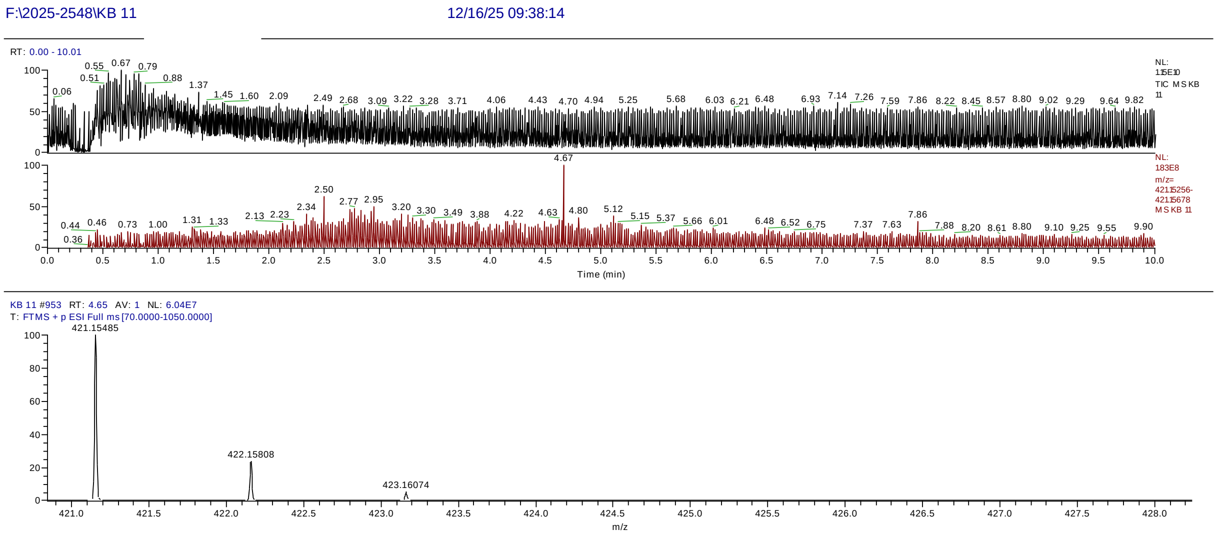

Figure S59: HRMS [M+H] spectrum of compound **19**

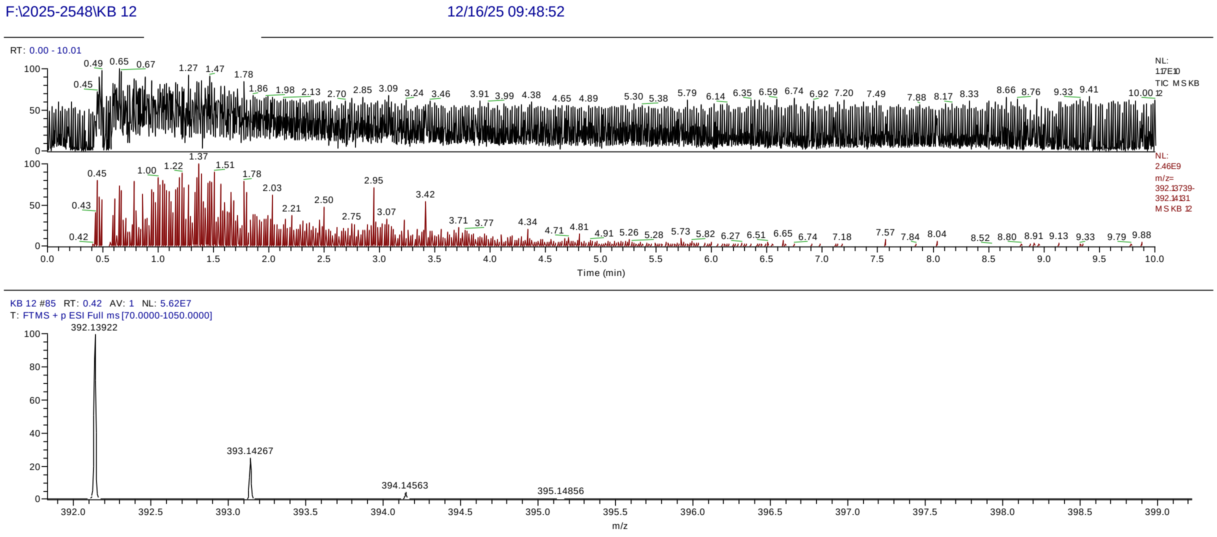

Figure S60: HRMS [M+H] spectrum of compound **20**

Compounds 1

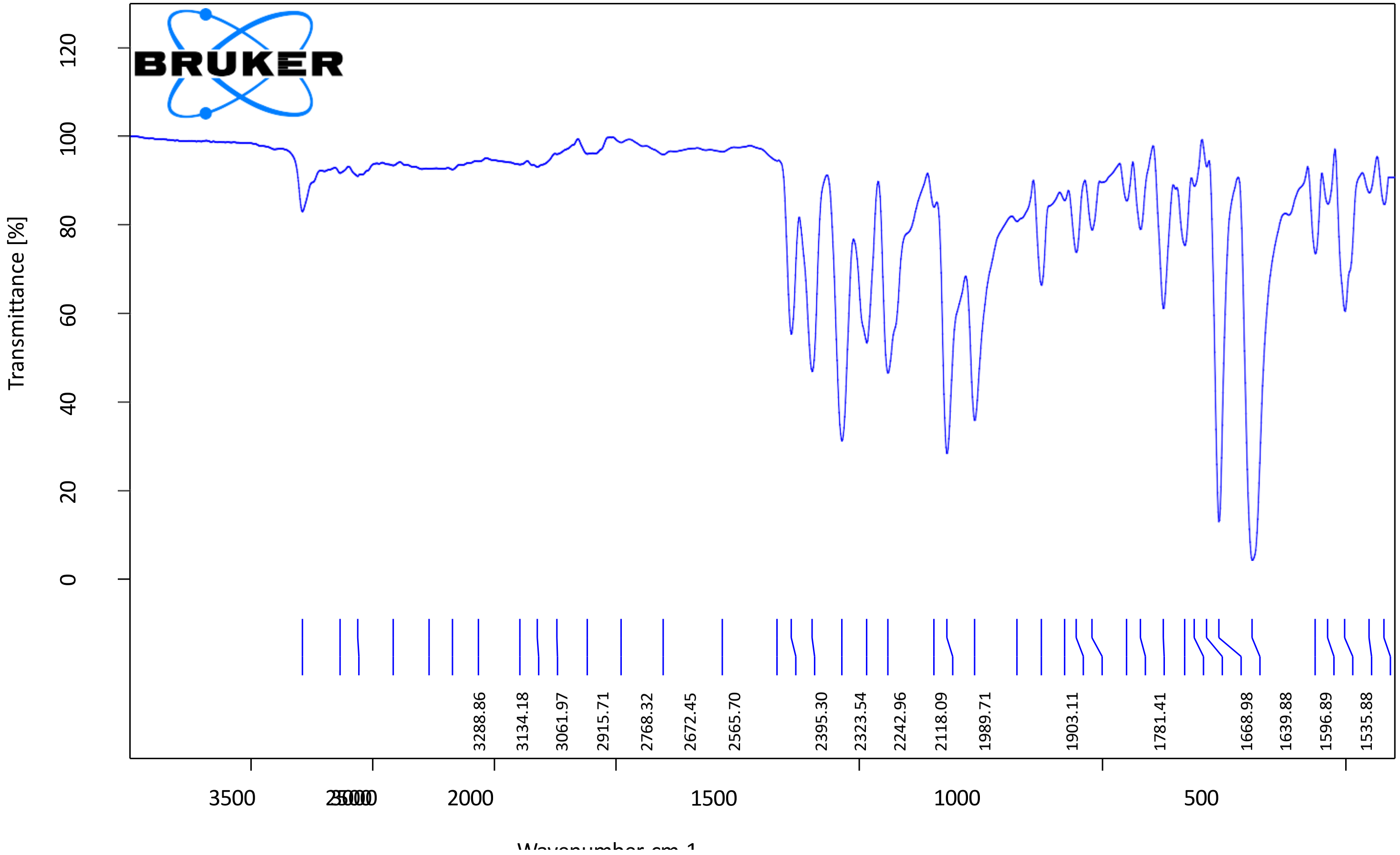

C:\Users\Hp\Documents\Bruker\OPUS_7.5.18\DATA\MEAS\No.10	Instrument type and / or accessory	22.12.2025	

Compounds 2

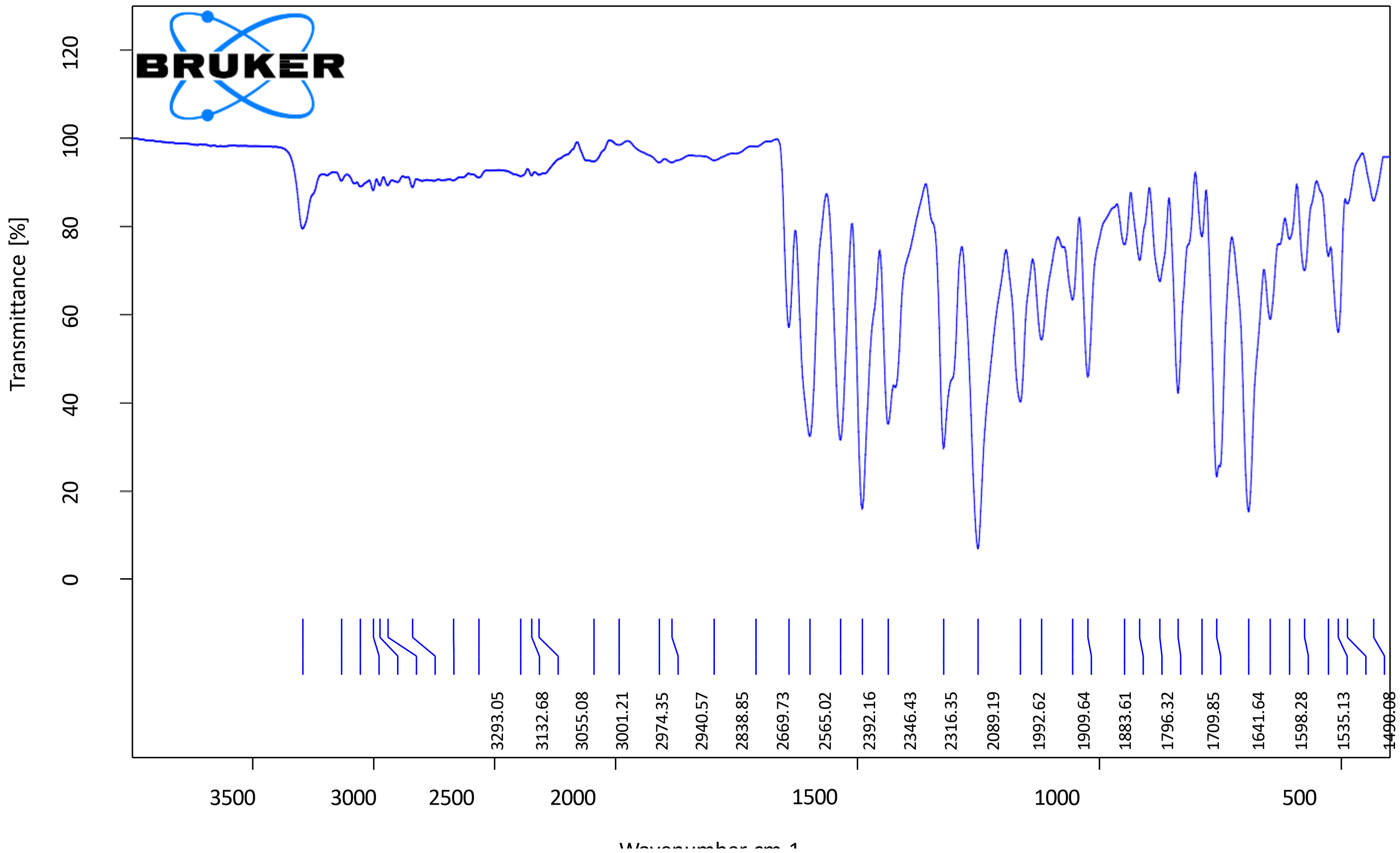

C:\Users\Hp\Documents\Bruker\OPUS_7.5.18\DATA\MEAS\No 2.0 No 2 Instrument type and / or accessory	22.12.2025

Compounds 3

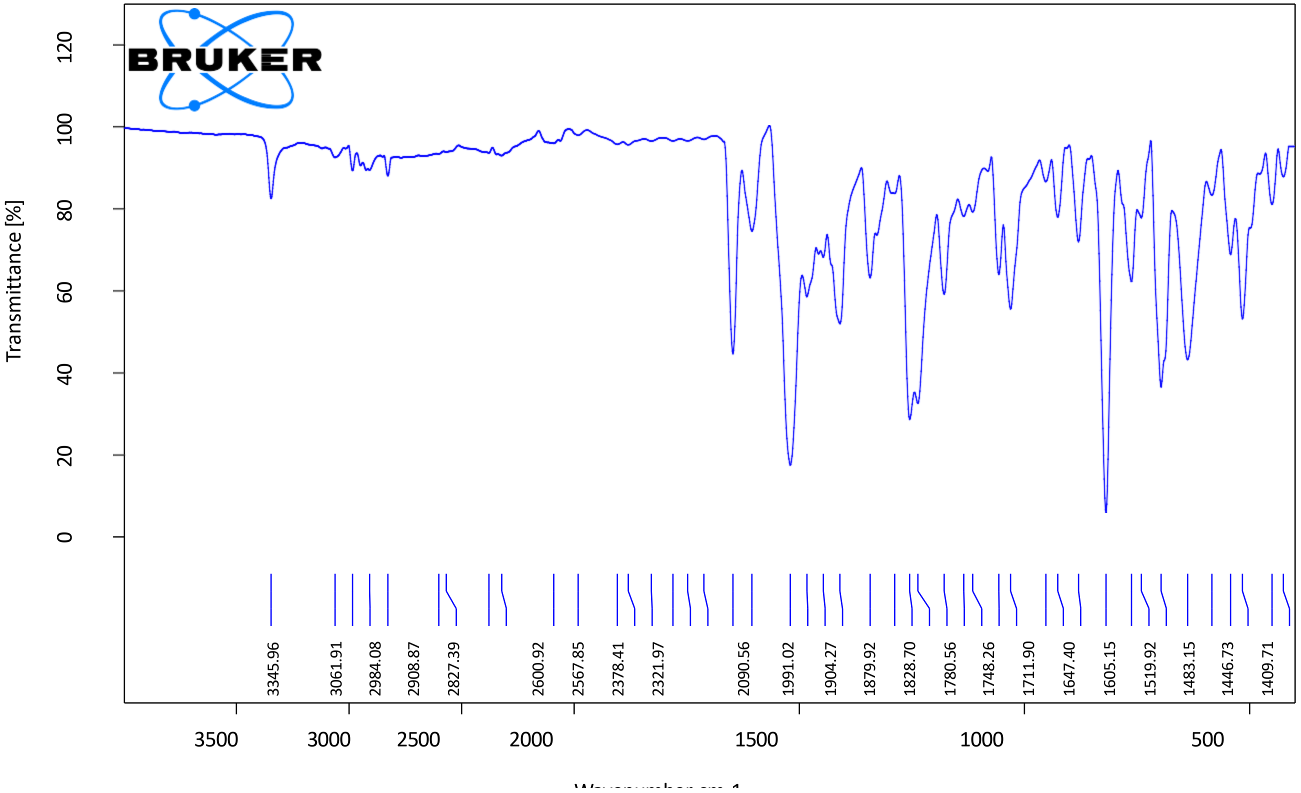

C:\Users\Hp\Documents\Bruker\OPUS_7.5.18\DATA\MEAS\No 3.0	No 3	Instrument type and / or accessory	22.12.2025

Compounds 4

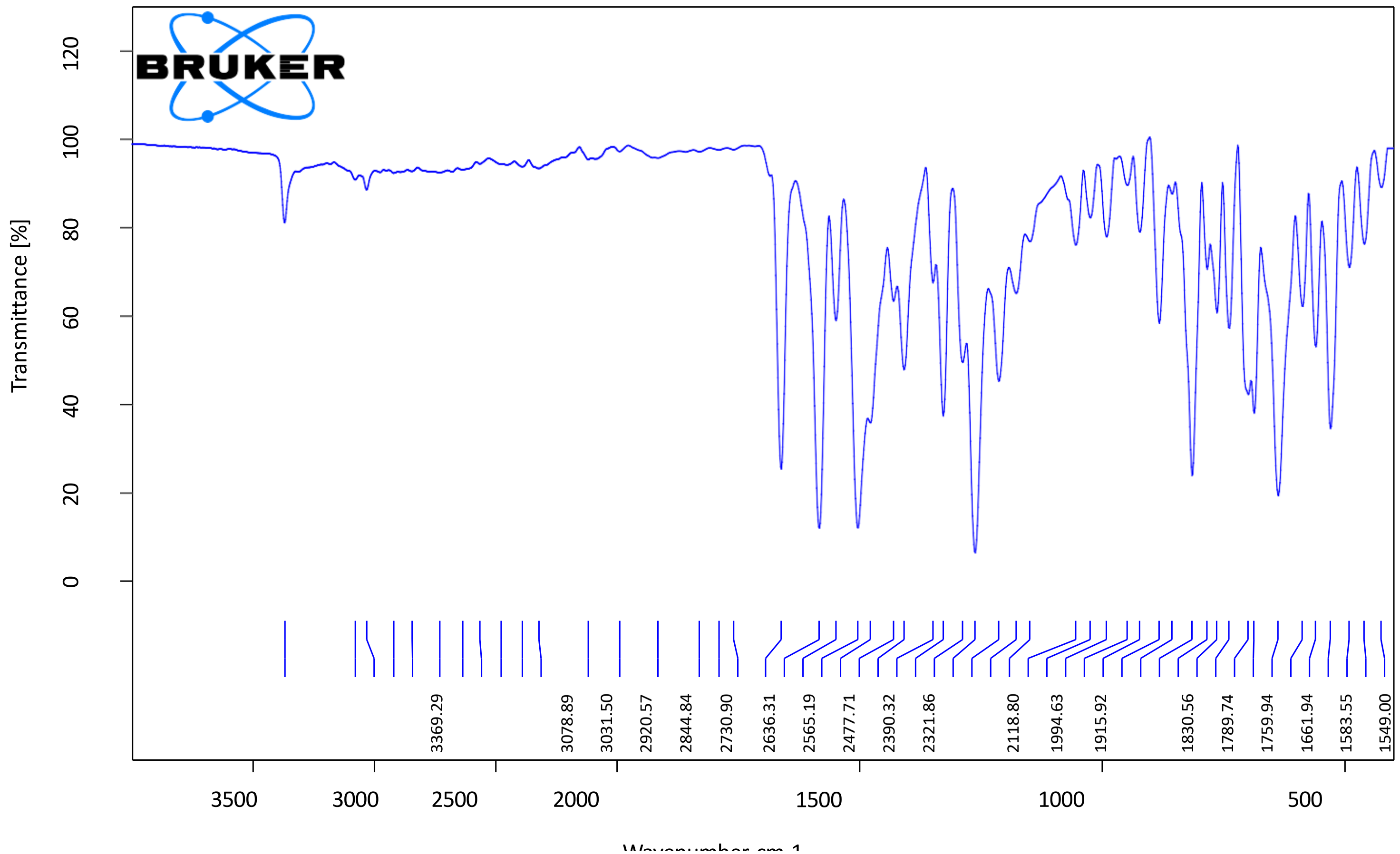

C:\Users\Hp\Documents\Bruker\OPUS_7.5.18\DATA\MEAS\KB-4.0	KB-4	Instrument type and / or accessory	22.12.2025

Compounds 5

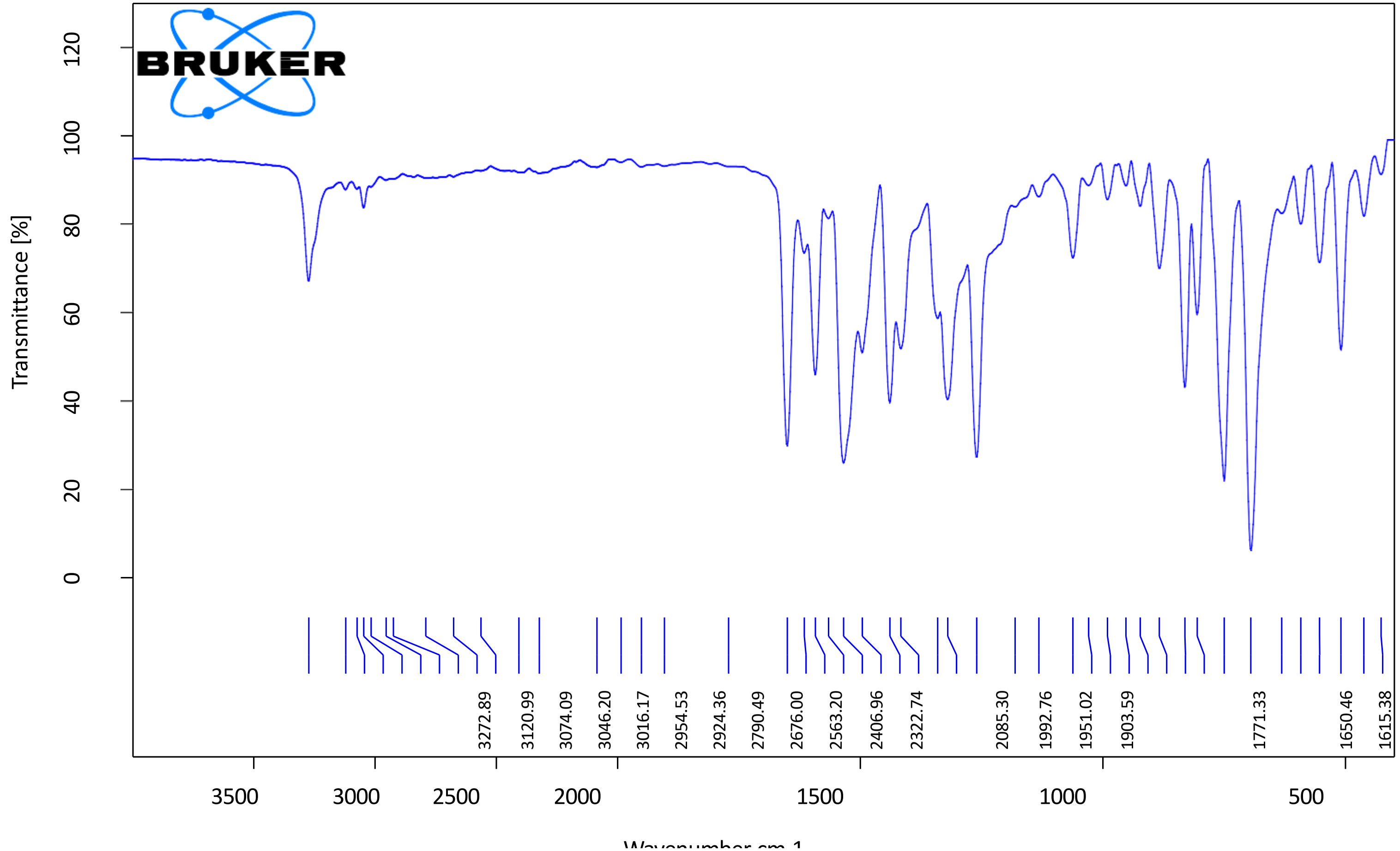

C:\Users\Hp\Documents\Bruker\OPUS_7.5.18\DATA\MEAS\KB-5.0	KB-5	Instrument type and / or accessory	22.12.2025

Compounds 6

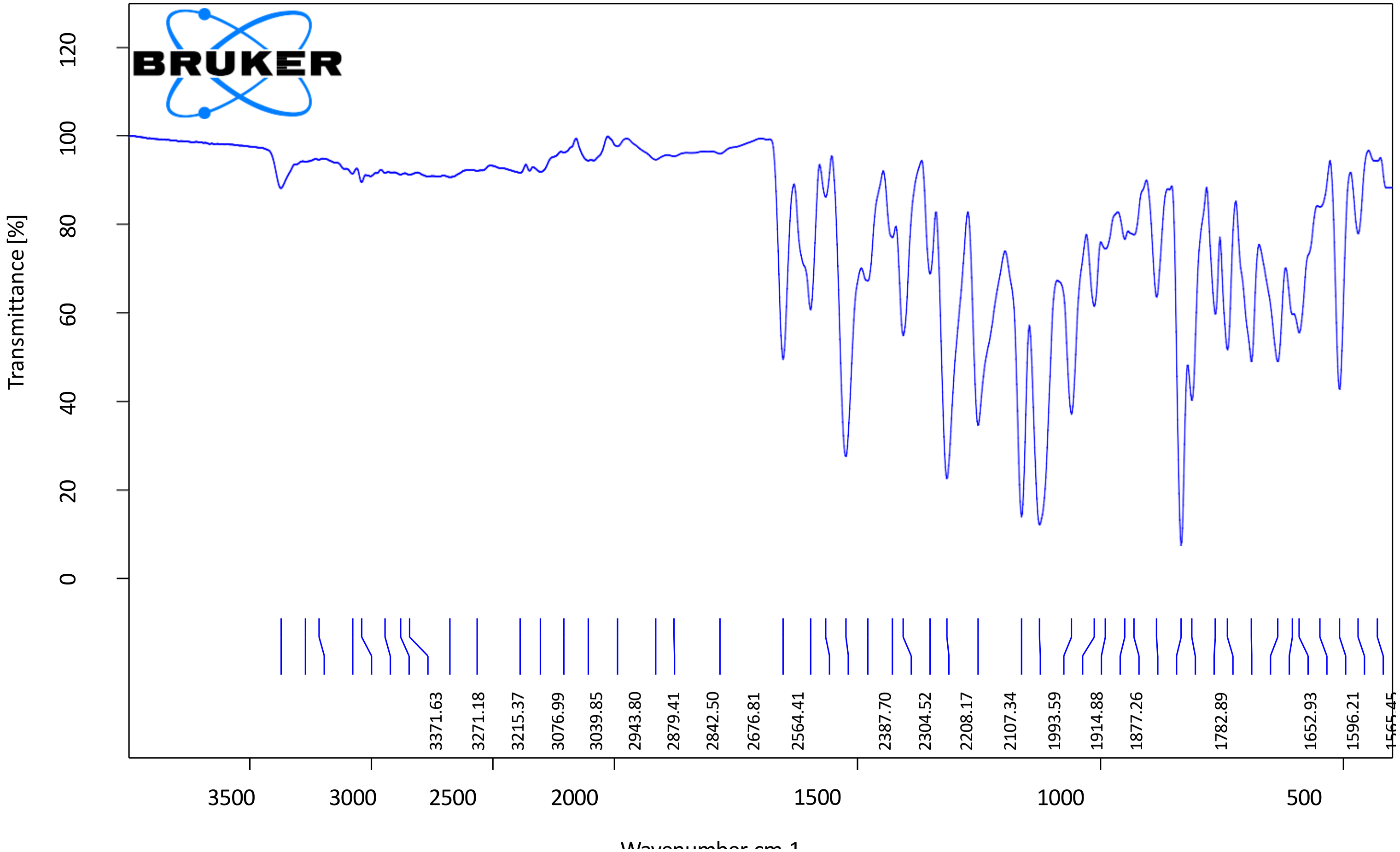

C:\Users\Hp\Documents\Bruker\OPUS_7.5.18\DATA\MEAS\No 6.0 No 6 Instrument type and / or accessory	22.12.2025

Compounds 7

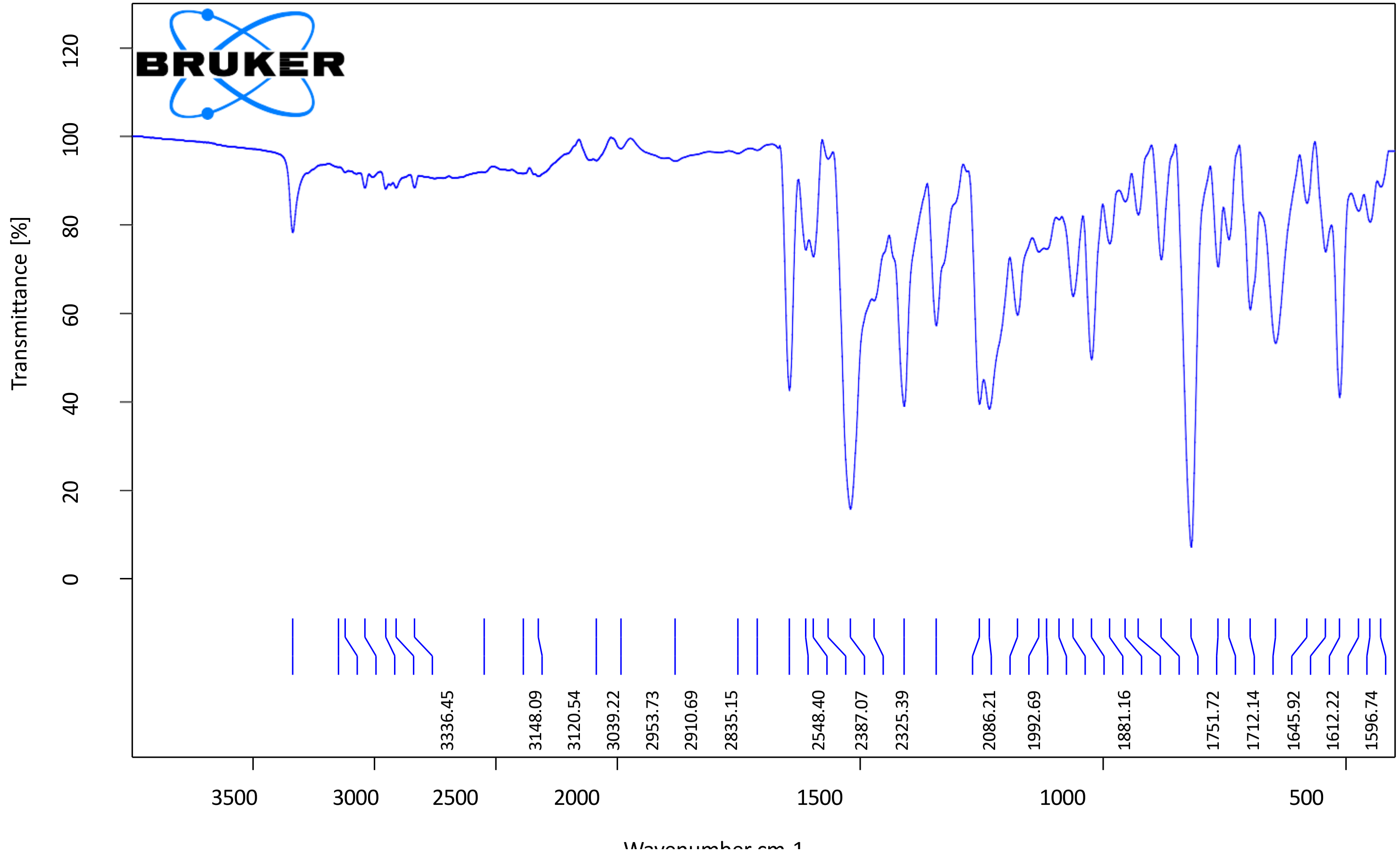

C:\Users\Hp\Documents\Bruker\OPUS_7.5.18\DATA\MEAS\No 7.0	No 7	Instrument type and / or accessory	22.12.2025

Compounds 8

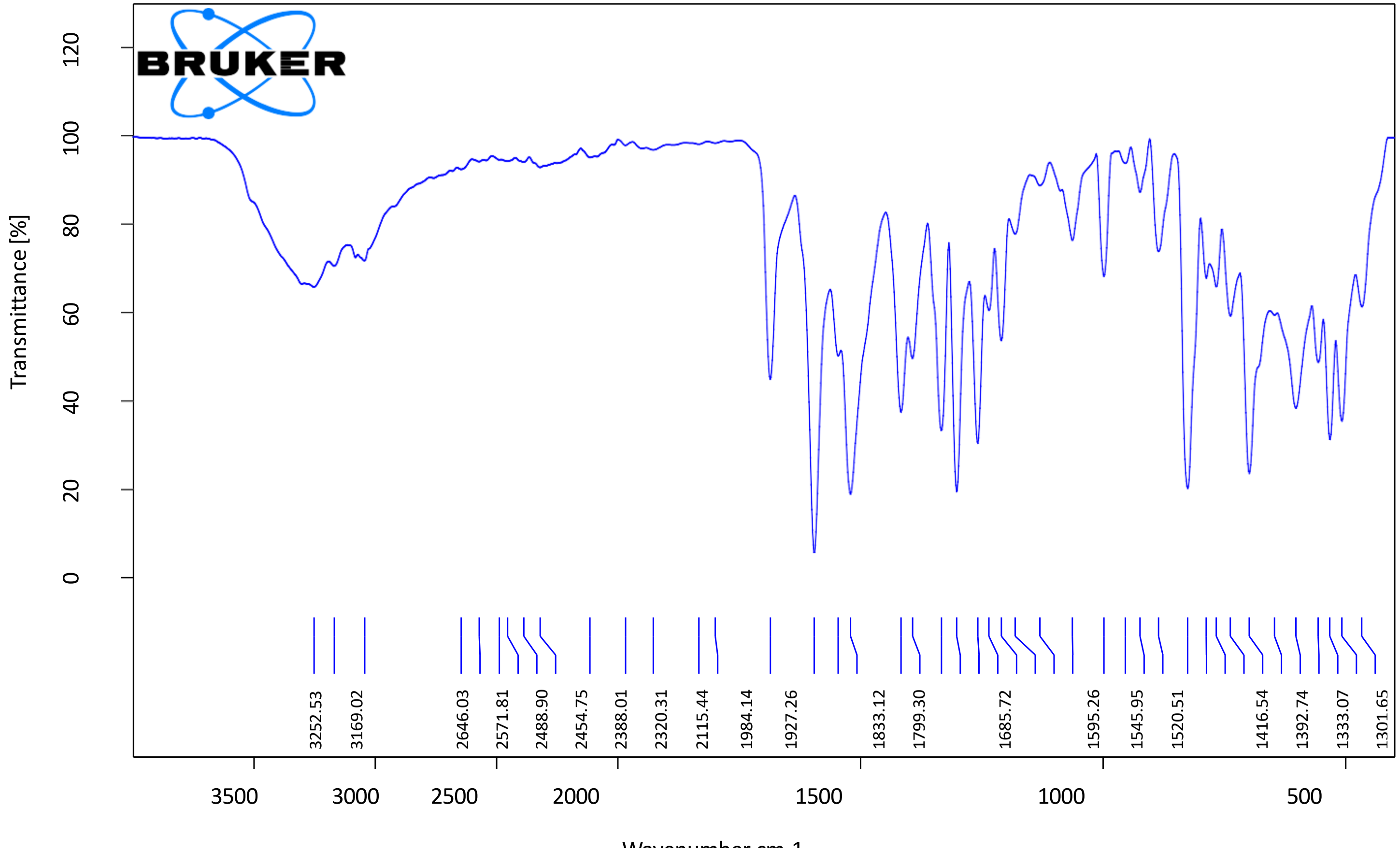

C:\Users\Hp\Documents\Bruker\OPUS_7.5.18\DATA\MEAS\No 8.0	No 8	Instrument type and / or accessory		22.12.2025

Compounds 9

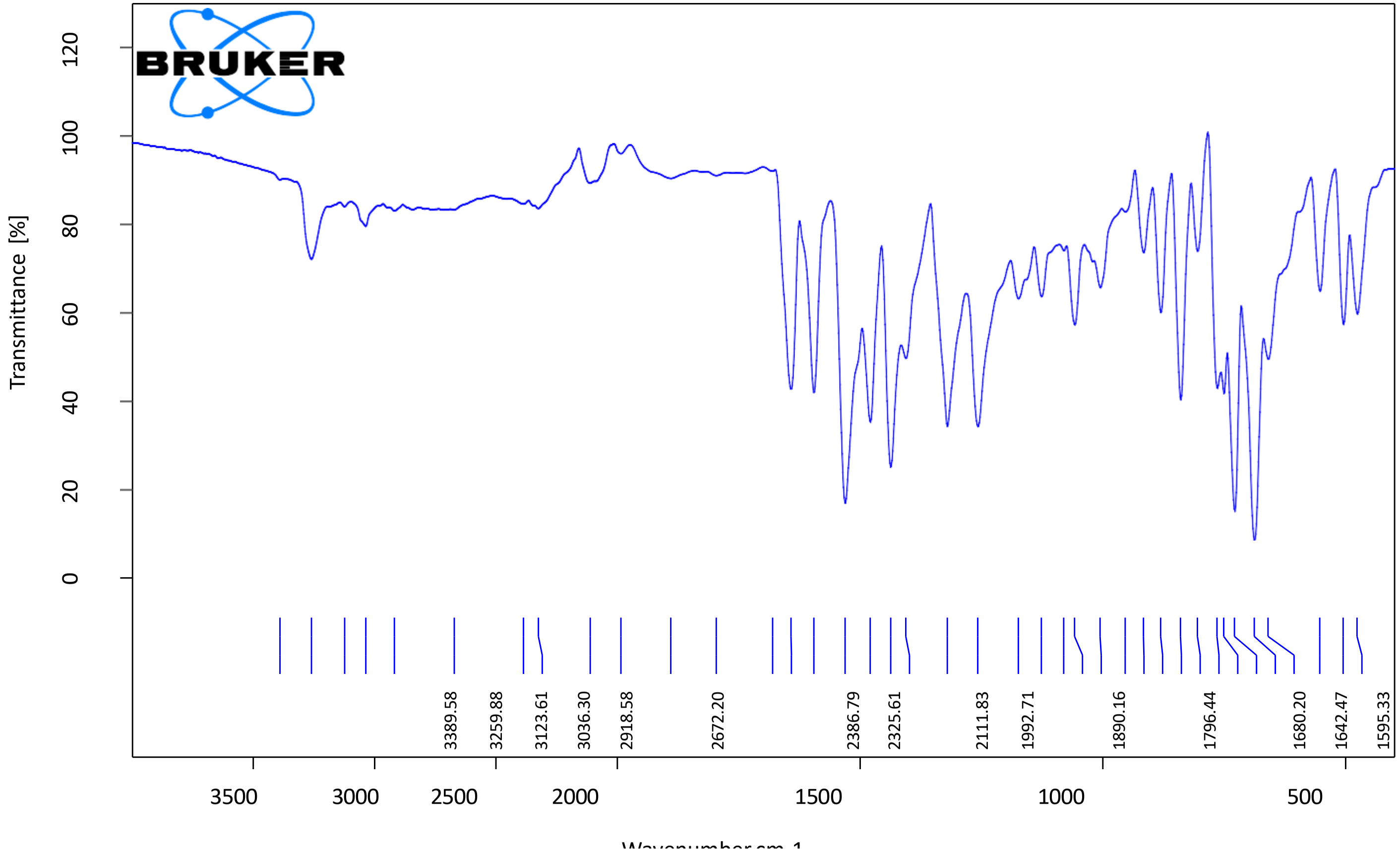

C:\Users\Hp\Documents\Bruker\OPUS_7.5.18\DATA\MEAS\KB-9.0	KB-9	Instrument type and / or accessory	22.12.2025

Compounds 10

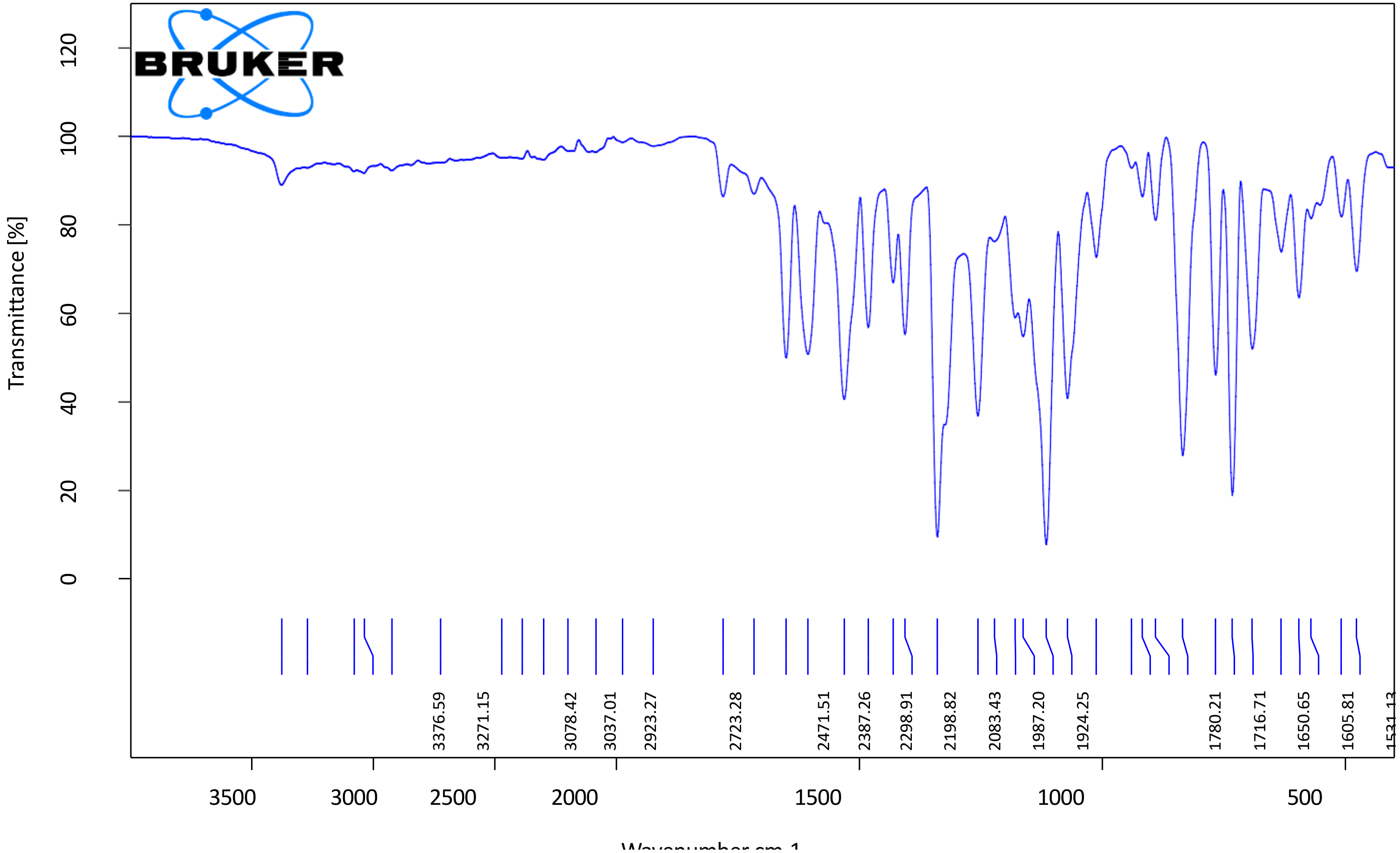

C:\Users\Hp\Documents\Bruker\OPUS_7.5.18\DATA\MEAS\No	10.0	No 10	Instrument type and / or accessory	22.12.2025

Compounds 11

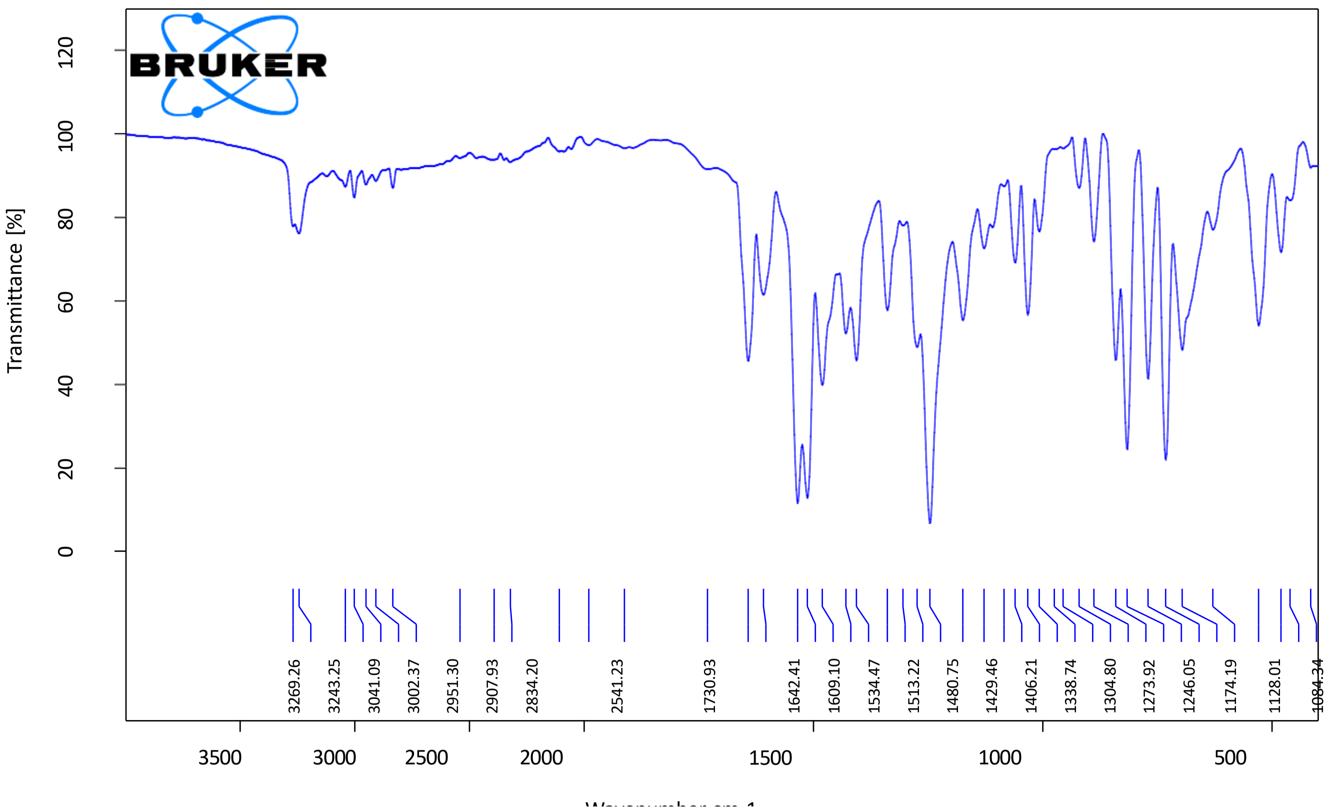

C:\Users\Hp\Documents\Bruker\OPUS_7.5.18\DATA\MEAS\KB-11.0 KB-11 Instrument type and / or accessory	22.12.2025

Compounds 12

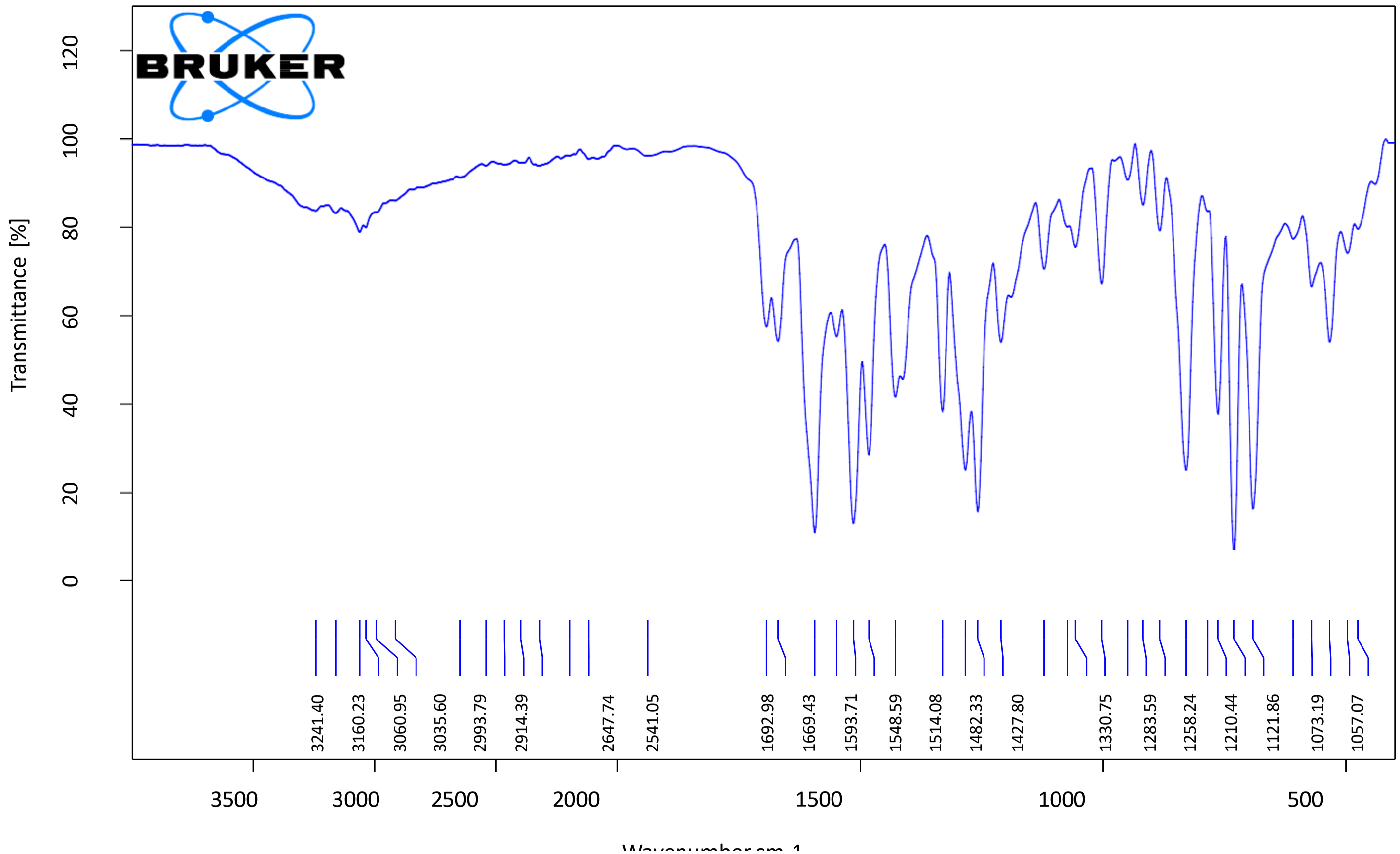

C:\Users\Hp\Documents\Bruker\OPUS_7.5.18\DATA\MEAS\No 12.0	No 12	Instrument type and / or accessory		22.12.2025

Compounds 13

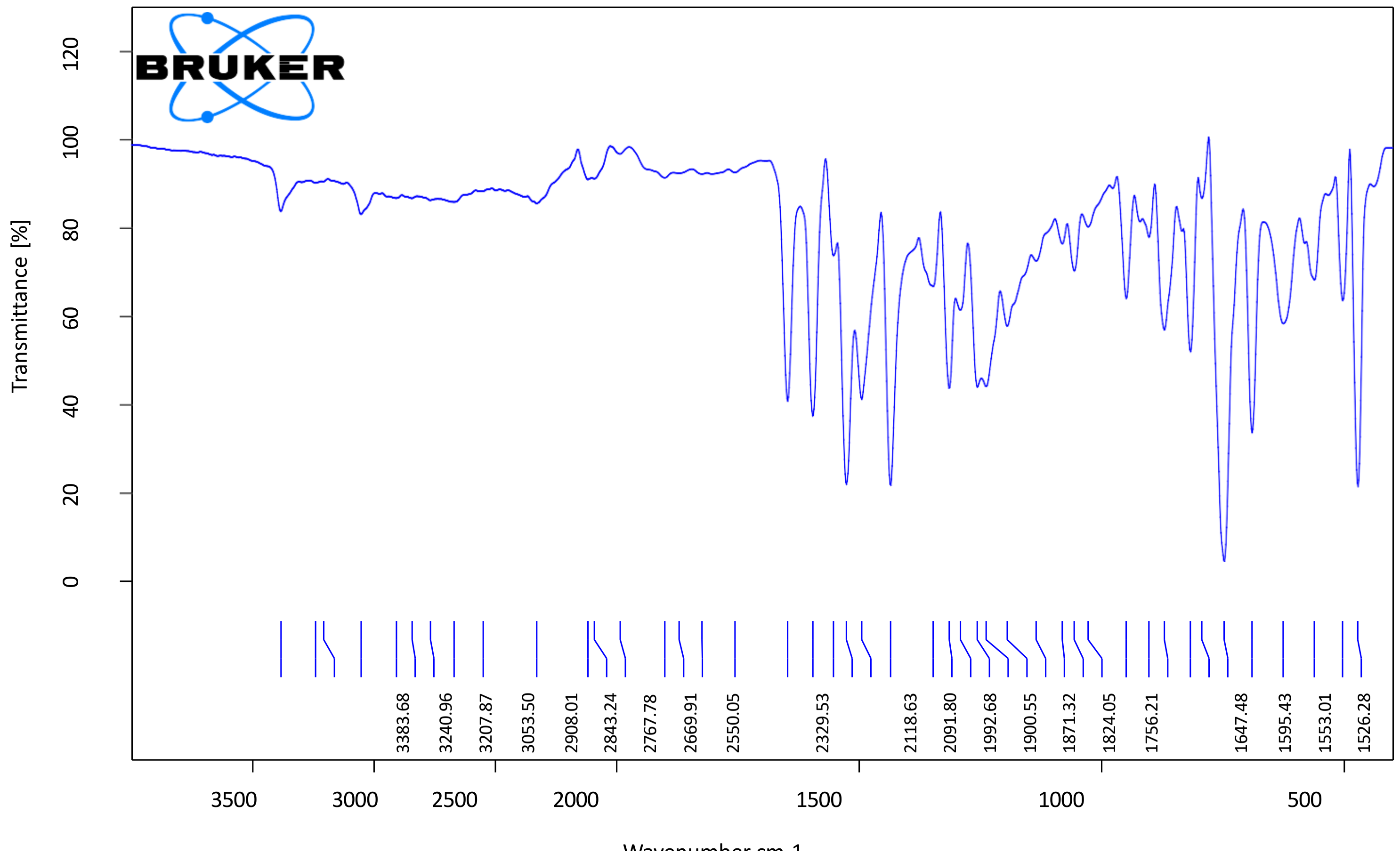

C:\Users\Hp\Documents\Bruker\OPUS_7.5.18\DATA\MEAS\KB-13.0	KB-13	Instrument type and / or accessory	22.12.2025

Compounds 14

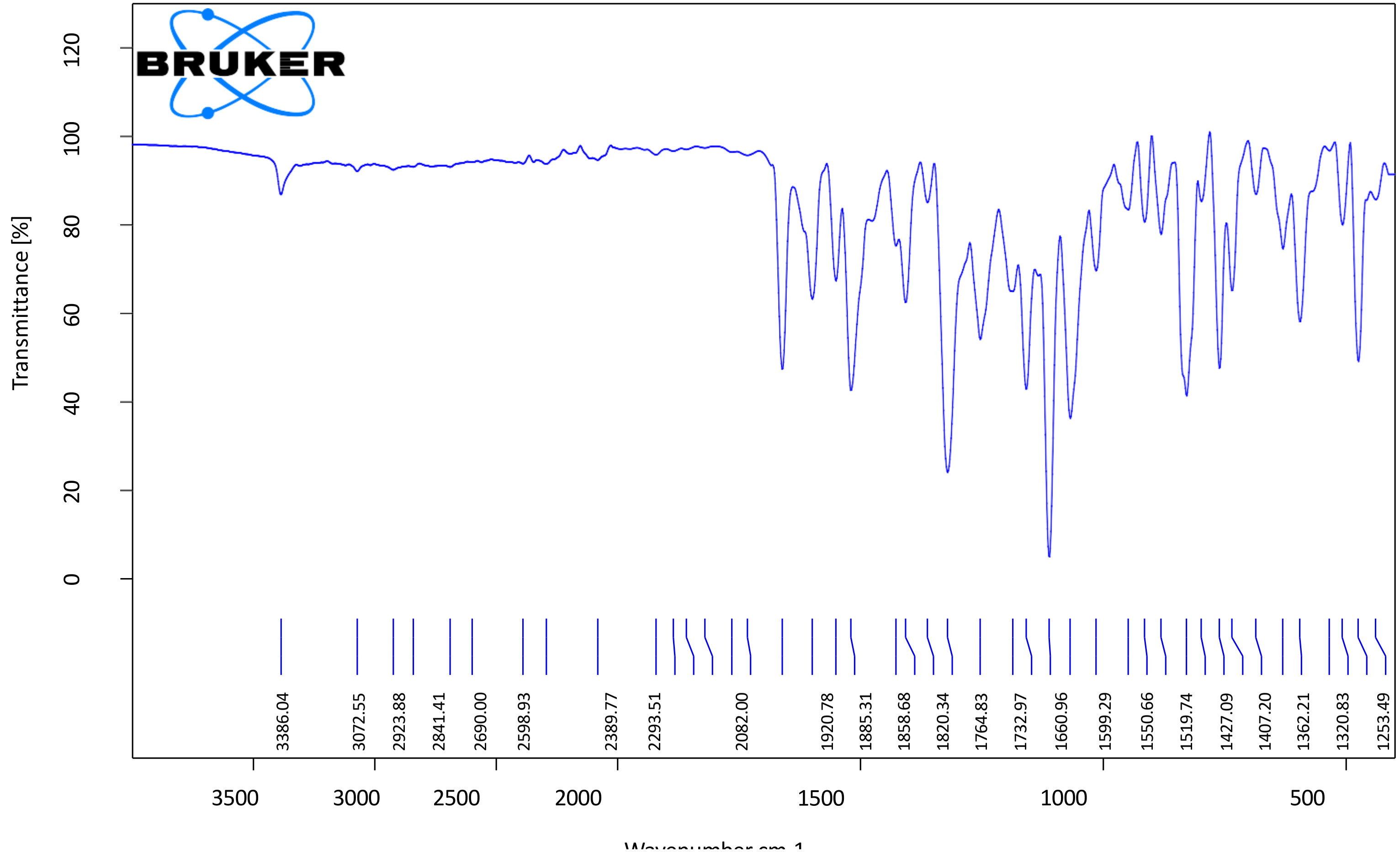

C:\Users\Hp\Documents\Bruker\OPUS_7.5.18\DATA\MEAS\No 14.0	No 14	Instrument type and / or accessory	22.12.2025

Compounds 15

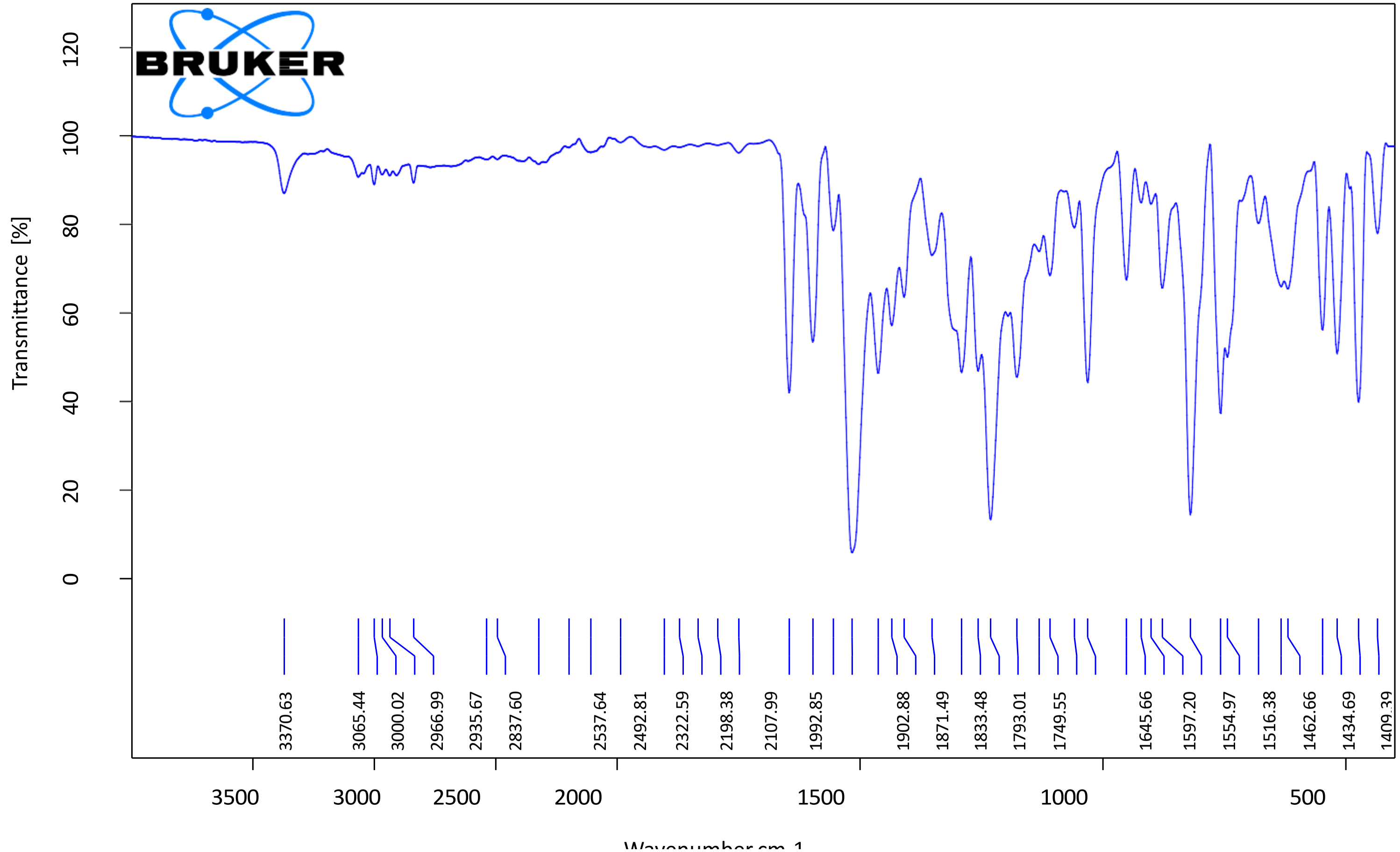

C:\Users\Hp\Documents\Bruker\OPUS_7.5.18\DATA\MEAS\K6-15.0	K6-15	Instrument type and / or accessory	22.12.2025

Compounds 16

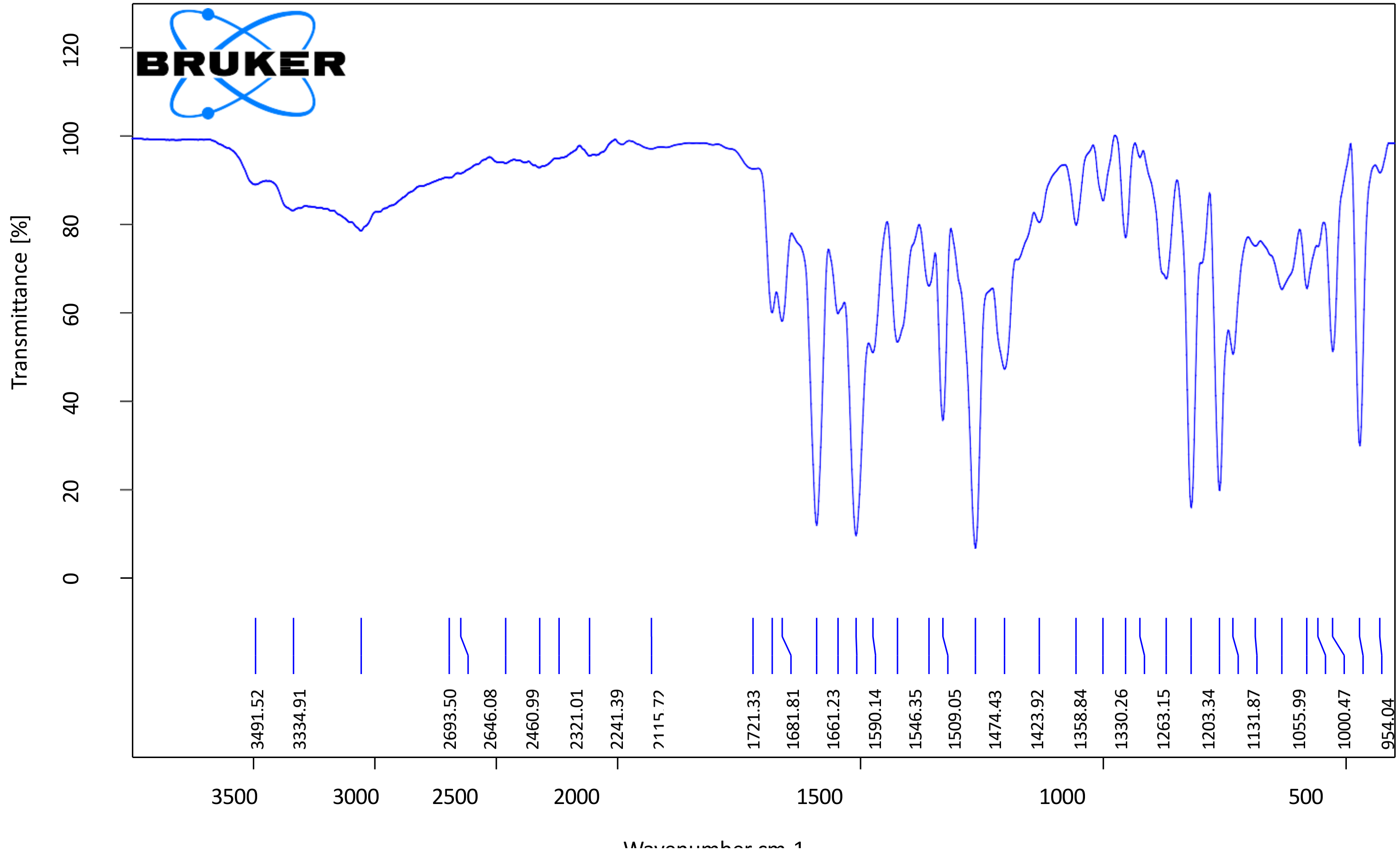

C:\Users\Hp\Documents\Bruker\OPUS_7.5.18\DATA\MEAS\K6-16.0	K6-16	Instrument type and / or accessory	22.12.2025

Compounds 17

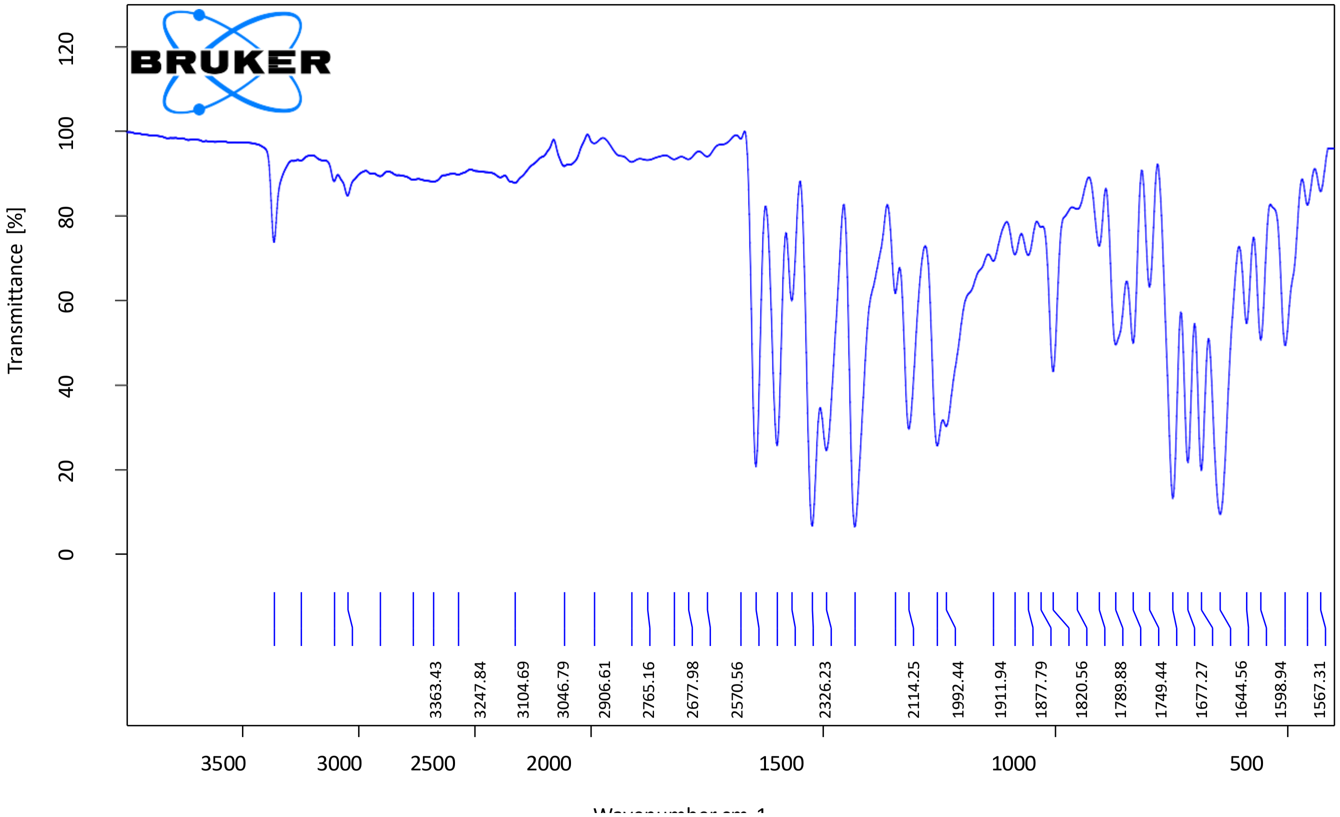

C:\Users\Hp\Documents\Bruker\OPUS_7.5.18\DATA\MEAS\17.1	17	Instrument type and / or accessory	22.12.2025

Compounds 18

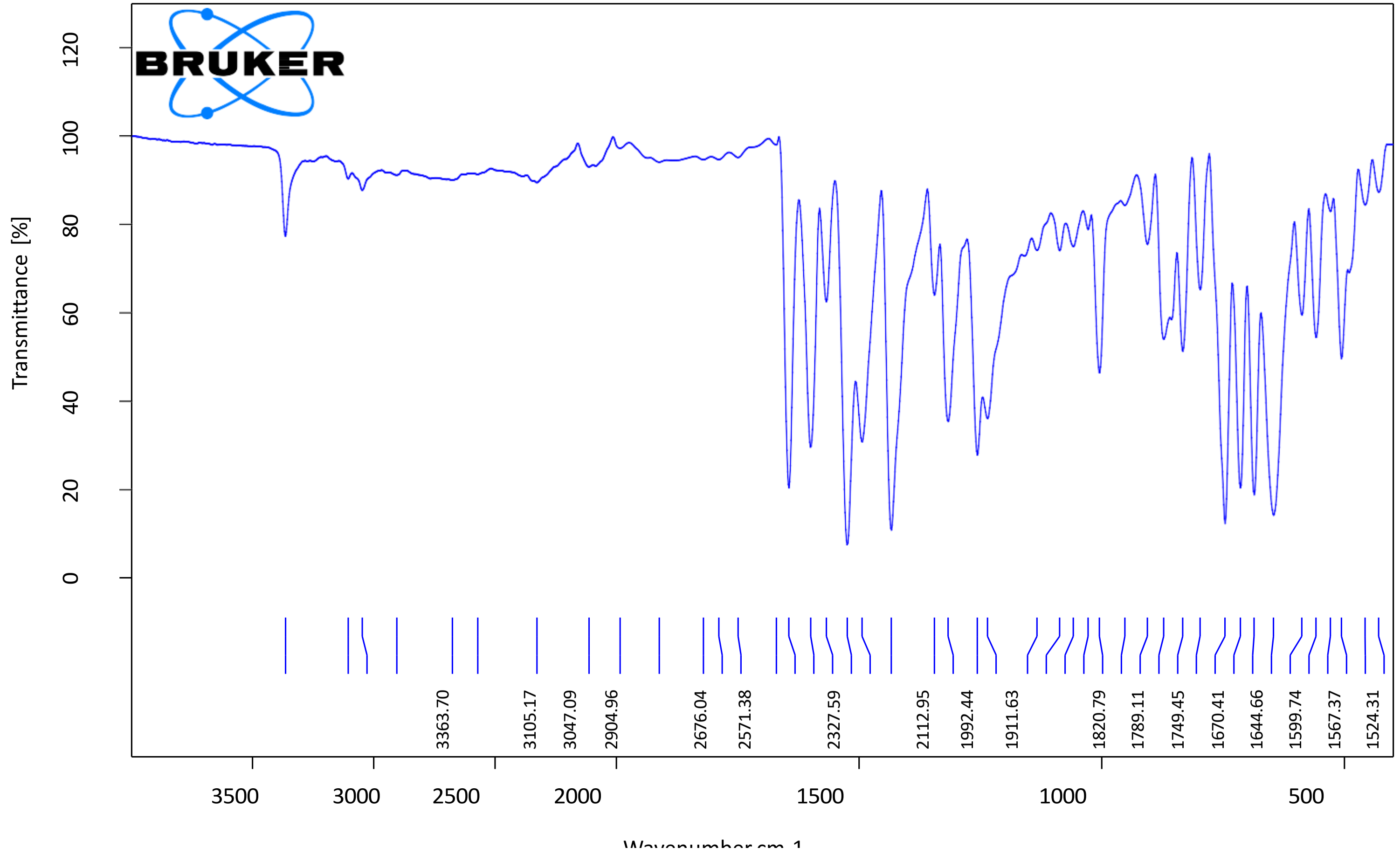

C:\Users\Hp\Documents\Bruker\OPUS_7.5.18\DATA\MEAS\No 18.0	No 18	Instrument type and / or accessory	22.12.2025

Compounds 19

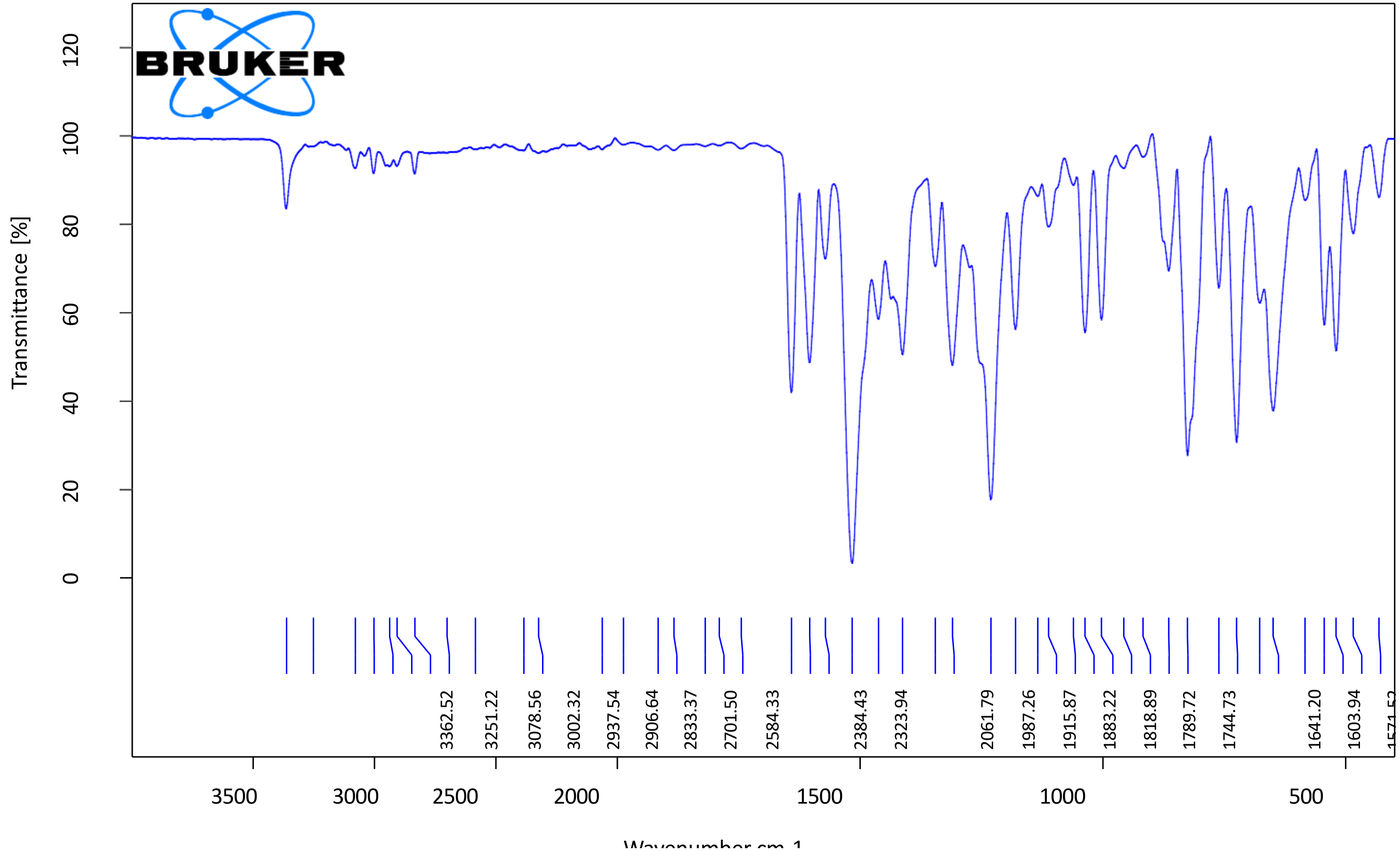

C:\Users\Hp\Documents\Bruker\OPUS_7.5.18\DATA\MEAS\19.0	19	Instrument type and / or accessory	22.12.2025

Compounds 20

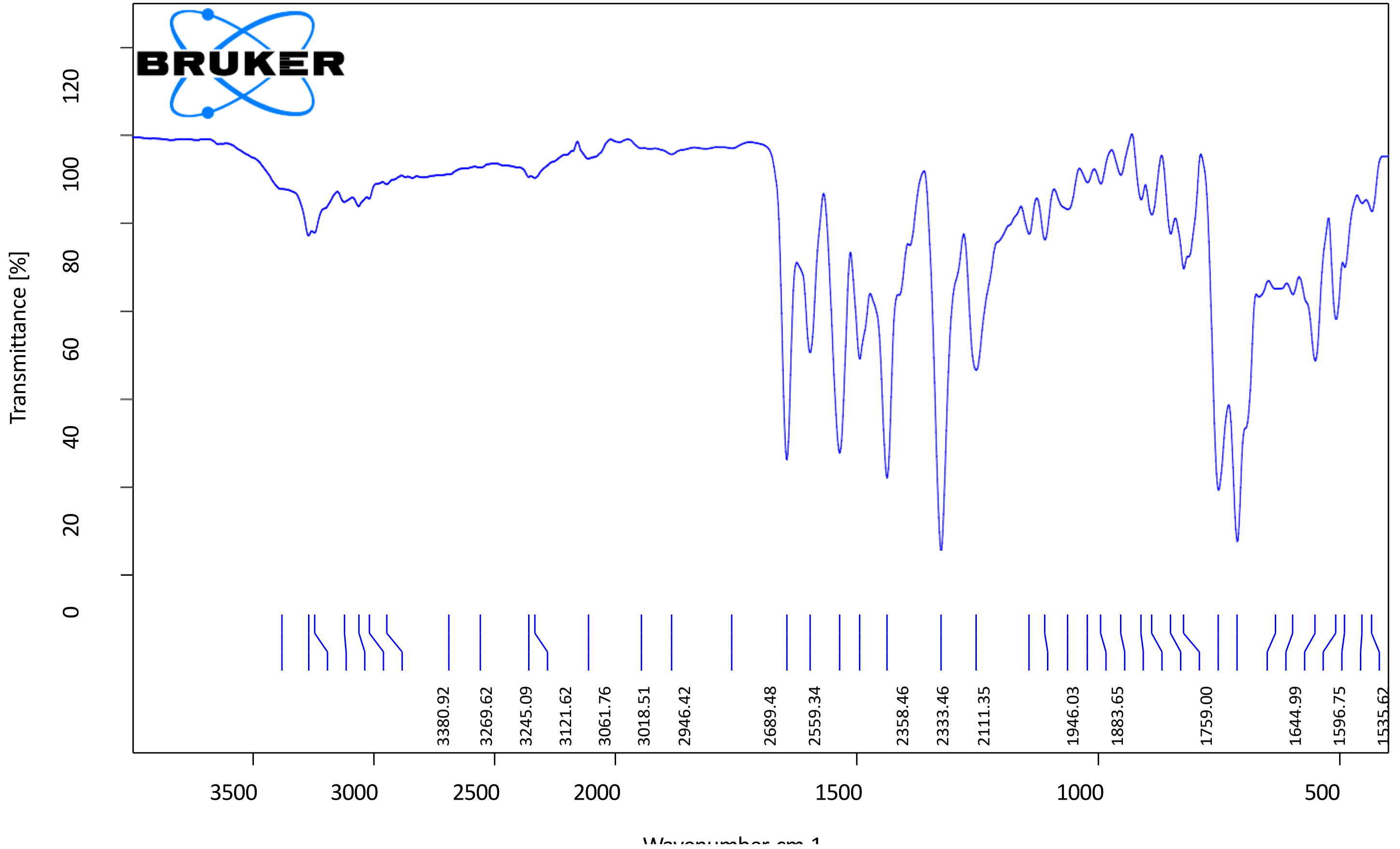

C:\Users\Hp\Documents\Bruker\OPUS_7.5.18\DATA\MEAS\20.1	20	Instrument type and / or accessory	22.12.2025

Page 1/1
